# Sustainable and biocompatible hybrid materials-based sulfated polysaccharides for biomedical applications: a review

**DOI:** 10.1039/d4ra07277d

**Published:** 2025-02-14

**Authors:** Reem S. Alfinaikh, Khalid A. Alamry, Mahmoud A. Hussein

**Affiliations:** a Chemistry Department, Faculty of Science, King Abdulaziz University P.O. Box 80203 Jeddah 21589 Saudi Arabia maabdo@kau.edu.sa mahussein74@yahoo.com kaalamri@kau.edu.sa; b Chemistry Department, Faculty of Science, Assiut University Assiut 71516 Egypt

## Abstract

Sustainable biomaterials that are both efficient and environmentally friendly are the subject of research and development efforts among scientists and academics from a variety of contemporary scientific disciplines. Due to their significant involvement in several physiological and pathological processes, sulfated polysaccharides (SPs) have garnered growing interest across various application domains, including biomedicine. Nevertheless, mechanical and thermal stability are issues for unmodified polysaccharide materials. Interactions between polymers, such as the mixing of biopolymers with synthetic or biopolymers through chemical interaction or grafting into the main chain structure of raw materials to enhance their therapeutic effects, are essential to meet the high standards of biomedical features. Another way to improve the mechanical and thermal properties is to graft appropriate fillers onto the polysaccharide backbone. The characteristics of polysaccharide bio-nanocomposites in comparison to more traditional polymers have attracted a lot of interest. With an emphasis on anti-inflammatory, anticancer, antiviral, immunoregulatory, and anticoagulant properties, this review delves into the most recent biological uses of sulfated polysaccharides. As well as thoroughly outlining the factors that impact the biological properties, such as the extraction process, molecular weight (Mw), the degree of sulfation, distribution/position, modification procedures, and the filler size, *etc.*, this review aims to: (1) provide a systematic and critical overview of the cutting-edge research on SPs and hybrid sulfated polysaccharide bio-nanocomposites; (2) identify the key factors, mechanisms, methods, and challenges impacting SPs bio-nanocomposites; (3) elucidate the current and potential biomedical applications, advantages, manufacturing challenges, and opportunities associated with SPs bio-nanocomposites; (4) offer insights into future research directions by suggesting improvements for bio-nanocomposites, including novel materials, and advanced processing techniques.

## Abstract 2

The work provides a systematic and critical overview of the cutting-edge research on SPs and hybrid sulfated polysaccharide bio-nanocomposites and identifies the key factors, mechanisms, methods, and challenges impacting SPs bio-nanocomposites.

## Graphical abstract

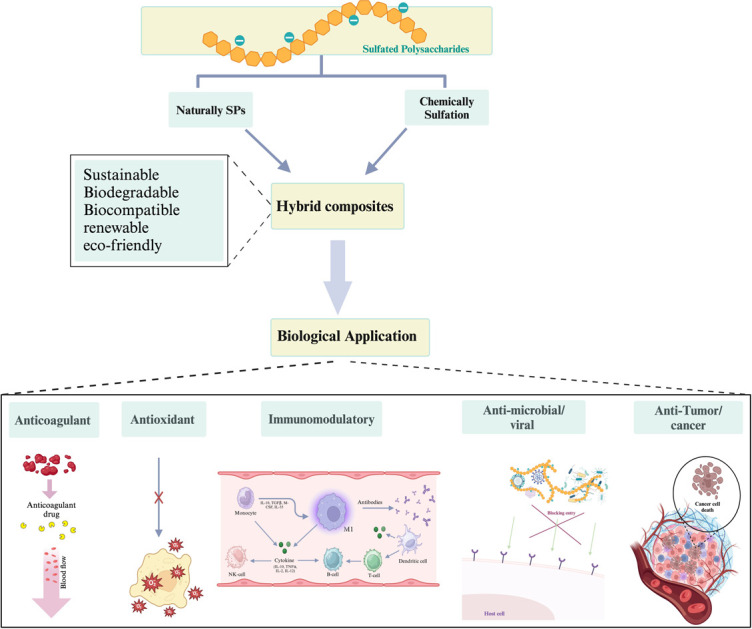

## 1. Introduction

Over the past few decades, significant research has been directed toward improving sustainable and renewable concepts to replace petroleum polymers with abundant, low-cost, biodegradable, and eco-friendly natural polymers available in nature. Thus, natural polymer materials have become new materials in various applications for renewable resources and overcome environmental issues.^1^ Polysaccharides, classified as natural polymers, are composed of many blocks of monosaccharide units linked together with glycosidic bonds.^2^ Polysaccharides can be classified based on the composition of their monomers; they can be either homogeneous (such as starch, cellulose, and glycogen) or heterogeneous, like hyaluronic acid, chondroitin sulfate, and alginate. Polysaccharides can be classified into anionic, neutral, and cationic types based on their charged groups. So far, nature has only given us one type of alkaline polysaccharide called chitosan, while the rest are mostly acidic or neutral polysaccharides.^3^ Natural polysaccharides have fewer side effects, yet their inherent physicochemical features have hindered the evaluation of their bioactivities in comparison to synthetic pharmaceuticals. As a result, researchers have modified the systems and characteristics of natural polysaccharides according to structure–function correlations, leading to the creation of more functionally efficient polysaccharides.^4^

Natural sulfated polysaccharides have attracted considerable attention because of their impressive ability, biocompatibility, biodegradability, non-toxic nature, renewable, biologically tunable, inertness nature, swelling and colloidal features, ease of modification, being present in a wide range of living species, and serving a variety of biological functions based on their chemical structure and interactions with other bioactive substances.^5–7^ Sulfated polysaccharides (SPs) are negatively charged polysaccharides that do not affect pH, in contrast to carboxylated polysaccharides and most likely found in the cell walls of marine seaweeds.^7,8^ The negative charge is caused by the cross-linking of sulfate group ions with complicated polysaccharide molecules. Sulfate groups are incorporated in the backbone of their sugar structure to withstand harsh marine conditions such as high salinity, which causes changes in their polymeric structure, resulting in SPs with high biological activity and commercial applications.^7^ The anionic characteristics of sulfated polysaccharides also facilitate the construction of biomaterial structures such as hydrogels, films, or fibers, which are advantageous for drug delivery systems, scaffold for tissue engineering, and more. In addition, sulfated polysaccharides have antiviral, anticoagulant, and anti-inflammatory properties, making them potential candidates for therapeutic uses. They may also be used in tissue engineering and regenerative medicine due to their capacity to interact with proteins and cells.^7^

Introducing chemical modifications to unsulfated polysaccharides can help overcome their drawbacks since the introduction of additional functional groups, such as sulfate groups, enhances their reactivity. Most polysaccharides possess a hydroxyl group, enhancing their stability and reducing their energy, which leads to diminished chemical reactivity. Polysaccharides undergo modifications by chemical, physical, and biological methods. The hydroxyl group in polysaccharides enables several chemical changes, including sulfonation, phosphorylation, oxidation, and carboxymethylation. These chemical modifications improve features including physicochemical qualities and biological activity.^4,9^ The presence of numerous sulfates on a single polysaccharide facilitates an open, improved solution conformation, hence reducing electrostatic repulsion among the negative charges.^8^ As well as improving the biological activities of the polysaccharides, for example, the antiviral efficacy of polysaccharides is significantly influenced by the density and arrangement of sulfate groups along their structures. Elevated sulfate density results in a higher quantity of negatively charged sulfate groups that interact with viral surface proteins.^10^

Naturally or modified sulfated polysaccharides frequently do not fulfill present scientific criteria; however, using two or more components to generate composites might meet these more stringent standards.^11^ Bio-composites, achieved by copolymer or grafted copolymerization, serve as an efficient method for improving the surface characteristics of polysaccharides.^12,13^ The primary aims of surface modification are to improve the mechanical and physicochemical characteristics of a polymer's surface relative to those of the unmodified sulfated polysaccharides individually.^13,14^ Nevertheless, green bio-composites possess some drawbacks, including a high water absorption rate, poor mechanical characteristics, weak thermal stability, and increased water absorption. Since these qualities are determined by a wide variety of parameters, the severity of these drawbacks differs between bio-composite types.^15^ The thermal stability of biopolymeric material is associated with the biopolymer's capacity to preserve its characteristics and structure under high-temperature conditions.^16,17^ An important factor in a material's characteristics is how it reacts to heat; this factor affects both its morphology and the effectiveness of its therapeutic applications. Implants typically make use of biopolymers, particularly those with electrical components and temperature-sensitive characteristics necessary for tissue interface with living organisms.^18^ In pharmaceutical delivery systems, some polymers are engineered to release therapeutic chemicals in a regulated manner over time. Changes in temperature have the potential to substantially impact the rate of medication release. Polymers exhibiting inadequate thermal stability may alter their characteristics with temperature variations, resulting in uncontrolled or premature medication release. Thermally stable polymers provide the precise delivery of the medicine, preserving its therapeutic effectiveness.^19^ In order to obtain better control over drug release, it is necessary to use a biopolymer that is thermally stable, which ensures that medicines will work as expected by reducing the risk of degradation or failure caused by heat.^19,20^ The thermal stability of a medicine is intrinsically linked to the shelf life of pharmaceutical goods, rendering it very pertinent to the pharmaceutical field. The World Health Organization (WHO) advises that the chemical and thermal stability of drugs be assessed to detect any degradation products in final medical formulations.^21^ Pharmaceutical stability testing is a critical examination of the alterations in the quality of a medicinal product over time, influenced by environmental elements such as temperature, humidity, and light. Stability testing is typically advised during the development of new pharmaceuticals to determine the product's shelf life and to suggest appropriate storage conditions.^22^ Reinforcement materials, including ceramics, nanoclay, and metal oxides with high crystalline planes, demonstrate superior high-temperature stability by inducing physical and chemical crosslinking within biopolymer matrices, therefore safeguarding the material from degradation due to heat stress. Previous research demonstrated that pure bacterial cellulose (BC) may thermally degrade at temperatures as low as 190 °C, which can be elevated to 580 °C by functionalization with an inorganic nanoparticle.^16^

Grafting appropriate nanofillers to SPs (natural or modified) possesses unique features that are unattainable in bulk materials at the macro scale of sulfated polysaccharides. SPs bio-nanocomposites are composites that integrate sulfated polysaccharides with inorganic or organic nanoparticles. Nanoparticles (NPs) are essential for making advanced bio-nanocomposites because they have outstanding mechanical, thermal, electrical, optical, and chemical properties, as well as a large surface area-to-volume ratio.^23,24^ SPs bio-nanocomposites have demonstrated the benefits of incorporating nanomaterials that are lacking in conventional biopolymers. The considerable surface area-to-volume ratio of nanoparticles permits even a little quantity inside the matrix to have a large impact on the SP's physical and material properties.^6^ Moreover, a larger surface area enhances the capacity for biological activities, including the increased attachment of anti-cancer agents^25^ and increased interaction with viral surface proteins.^10^ Additionally, these materials can assume virtually limitless shapes due to precise design.^26^ Furthermore, it utilizes the unique properties of polymers and nanostructures to create multifunctional, innovative materials.^6^ In this context, SPs bio-nanocomposites play a crucial role in the advancement of therapeutic applications, including their capacity to prevent blood clots, combat inflammation, enhance the immune system, eradicate microbes, and combat tumors and cancer.^7^

Consequently, SPs bio-nanocomposites can address some issues faced by SPs while simultaneously revealing innovative biological uses. The creation of hybrid materials that combine biological functions with other desirable characteristics inside a biodegradable and biocompatible SPs matrix is a primary emphasis in contemporary biomedical research and applications. Recent advancements in biomedicine, biotechnology, pharmaceuticals, material science, and academia underscore the necessity for additional composite research, particularly concerning SPs biocomposites and SPs bio-nanocomposites, as their potential to fulfill current demands for technological progress significantly surpasses that of the raw materials. This review discusses renewable sulfated polysaccharides that can be utilized to create novel biocomposites and bio-nanocomposites with distinct, desirable characteristics.

## 2. Sulfated polysaccharides (SPs)

### 2.1. Classification and sources of sulfated polysaccharides

**Fig. 1:**
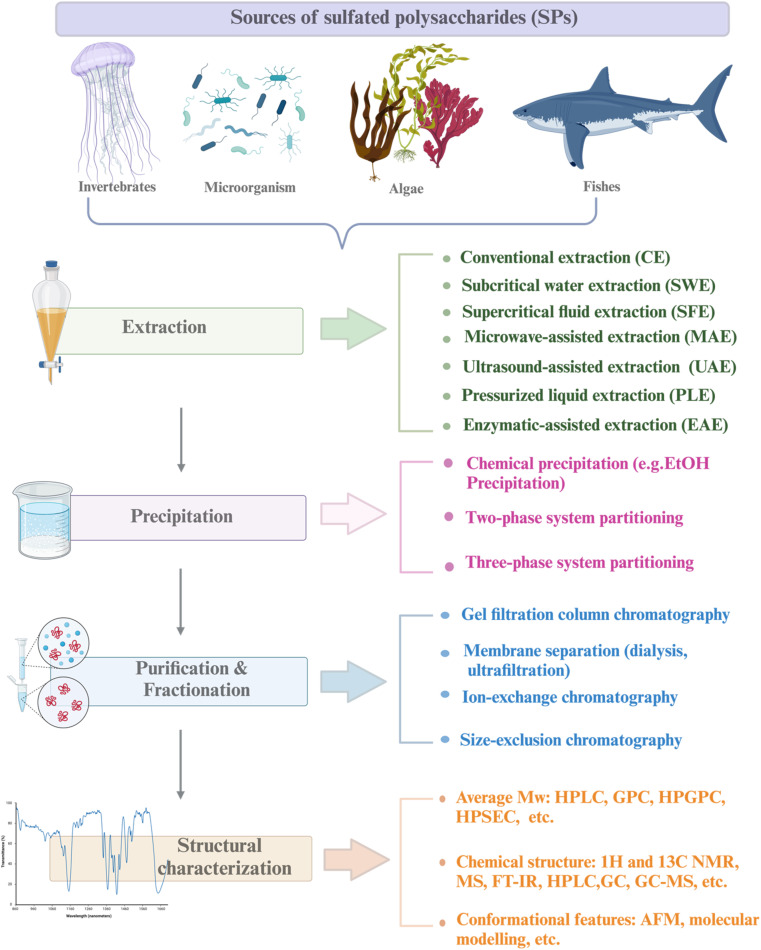
Roadmap of approaches/methods of marine sulfated polysaccharides and their sources. Created in BioRender. Alfinaikh R. (2025) https://BioRender.com/v95d222.

SPs are classified based on their sources, solubilities, and chemical composition. The chemical composition is composed of homopolysaccharides (consisting of a single unit of monosaccharide, for example, glycogen) and heteropolysaccharides (consisting of different units of monosaccharides, such as heparin). On the basis of their sources, SPs are usually categorized as animal-derived bioactive (dermatan sulfates, chondroitin sulfate, and heparin), plant-derived bioactive (sulfated galactan from the marine plant *Ruppia maritima*), microorganisms-derived bioactive (sulfated monophosphorylated mannose oligosaccharide, mushrooms, exopolysaccharides and capsular polysaccharides), and seaweed *etc.*^27,28^ Marine bioactive are the richest resource of SPs, among all of these, marine algae have the greatest number of SPs. Brown, green, and red alga are the three types of seaweed [Fig. 1] whose sulfated polysaccharide contents range from 4 to 76%, whereas green seaweed alone yields nearly 65% dry weight.^7^

#### 2.1.1 Chemical structures of polysaccharides

Among different kinds of carbohydrates, the most abundant in nature are polysaccharides, which are formed through the polymerization of monosaccharides (glycosidic bonds). These units may be cross linked or covalently linked to other molecules including peptides, proteins, amino acids, lipids, and some other chemicals.^12,29,30^ A polysaccharide will have a minimum of ten sugar units; anything less than which is classified as oligosaccharides. The chemical structure of the polysaccharides is usually regarded as (C_6_H_10_O_5_)_*n*_. The polysaccharides' characteristics reside in the repeating units by their properties, such as the configuration of the ring, the size of the rings, and the linkage type. d-Glucose, d-xylose, d-mannose, d-fructose, d-galactose, l-galactose, l-altrose, and l-arabinose are the primary components found in polysaccharides. Polysaccharides contain various monosaccharide derivatives, such as amino sugars (d-glucosamine and d-galactosamine), their derivatives (*N*-acetylneuraminic acid and *N*-acetylmuramic acid), and simple sugar acids (glucuronic and iduronic acids), among others; the confirmation d and l emphasize to differentiate between the two stereoisomers.^12^ Monosaccharides are linked with α-1,4-,β-1,4- and α-1,6-glycosidic bonds. The α and β classification depends on the position of the hydroxyl–OH on C-1 carbon and the hydroxy–OH at the terminal end of the molecule. While a sugar residue has only one anomeric carbon and can only create one glycosidic bond with hydroxyl groups on other molecules, it does have many hydroxyls, some of which can accept glycosyl substituents. Polysaccharides are unique in their capacity to create branched structures, unlike proteins and nucleic acids, which exist solely as linear polymers.^29^ The glycosidic linkage occurs through the anomeric carbon atom connecting the glycosidic bond donor and acceptor. It leads to the formation of chains that are either linear in longitudinal forms, or branched chains, which is different from the proteins and the peptides that have only linear chains.^30^ In algae, cell walls consist of two parts: (i) so-called, the ‘crystalline’ phase, which is an inert structural support and acts like a skeleton, and (ii) a bulk or poorly ordered phase known as matrix which houses skeleton. Regardless of whether the algae are green or brown, the crystalline phase of all three types of macroalgae is composed of cellulose (β-(1 → 4)-d-Glcp). A direct outcome of the photosynthetic process is the storage (reserve) of polysaccharides. Algae plastids store these nutrients, which may be used whenever the cell's metabolic needs them. Brown algae include β-(1 → 3)-d-glucans, also known as laminarin, while green and red seaweeds contain α-(1 → 4) and α-(1 → 6)-d-glucans, which are structurally like the starch found in terrestrial plants. In contrast, amorphous phase matrix polysaccharides vary greatly between algal classes; for example, brown and red algae's polysaccharides have many biological uses as drug delivery.^12^

### 2.2. Extraction, purification, and characterization of SPs

Extraction is a significant step to obtain SPs since, seaweed's bioactive, environmental conditions, extraction processes, and treatment techniques all affect the physicochemical quality of SPs' compounds. Because of differences in active growth factors and extraction circumstances, each new SPs isolated is a one-of-a-kind molecule with distinct structural properties, offering a possible novel medicine. As a result, a thorough comprehension of these factors will enable us to pinpoint the best procedures for obtaining high-quality SPs for applications.^31^ For example, in the medical industry, SPs are extracted using an enzyme-assisted extraction process to keep their biomedical qualities by safeguarding the bio-active compounds, and it is commonly utilized as a drug delivery helper. They are widely sought in food fortification because they have anti-coagulant, anti-inflammatory, anti-tumor, anti-viral, triglyceride, and cholesterol-lowering qualities.^7^ Traditionally, solid-to-liquid extraction using hot water and Soxhlet extraction are the most popular techniques for extracting SPs.^31^ In hot water extraction (HWE), the SPs are extracted by grinding the fruiting bodies and stirring them for many hours in hot water. This extraction process is straightforward to do, but it requires a lot of time, solvent, and heat.^32^ However, traditional extraction techniques currently have several drawbacks. High energy use, long procedure times, the use of great amounts and/or toxic solvents, and waste production are some of these drawbacks.^31,33^ New extraction methods could potentially lessen these negative effects and produce more sustainable and eco-friendly methods; thus, focusing on novel extraction methodologies need to be further assessed and improved in order to overcome these drawbacks.^33^ The molecular structure of SPs is dependent on the seaweed species, ages, extraction methods, and extraction conditions, including temperature, time, place of harvest, and season.^31,34,35^ Moreover, low pH enhances the selectivity of marine SPs, longer extraction times increase the extraction yield, and higher extraction temperatures permit greater solubilization of marine SPs. A low-temperature derived ulvan-type, for example, has the largest Mw (502 kDa at 35 °C and 286 kDa at 75 °C in water), maybe because high temperatures and an acidic pH prevent interchain bonding and ionic interactions. Additionally, the extraction method affected the content of sulfate groups and the purity of the extracted SPs, for instance, fucoidan heterogeneity was evaluated in response to aqueous and acidic extraction techniques. The purest fucoidan was found in hot water extracts, which also had the highest concentration of fucose (Fuc). On the other hand, the amount of uronic acid contamination was highest, and the sulfate group was decreased in acidic extracts.^34^

#### 2.2.1 General procedures of extraction SPs

The samples could undergo pretreatment to remove substances (*e.g.*, pigments or lipids) that might obstruct the SPs extraction. Firstly, entails preparing the algae, which may involve washing to remove salt and contaminants. After that, freeze-drying or grind to get a uniform powder. Then, various methods are used to extract the algae. After this step, the algae are subjected to additional processing to isolate the valuable components. Alkali treatments are frequently used for alginates and carrageenan. The next step is to add ethanol to precipitate the SPs and purify them using chromatographic, dialysis, and filtration methods.^31^

#### 2.2.2 Green-innovative extraction techniques

Since the physicochemical characteristics are determined by the method used. Scientists have placed significant efforts to innovate a green extraction method. Green extraction resulted in reduced extraction time, minimized the use of extraction solvents, preserved the bioactivities of the polysaccharides, and was energy efficient.^27^ Environmentally friendly solvents, like ionic liquids, eutectic solvents, surfactants, or solvents derived from biological sources, are an alternative method to reduce the negative effects of toxic chemicals used in the extraction process. Compared with organic solvents, environmentally friendly solvents are biodegradable, cost-effective, biopolymer dissolving, and recyclable (*e.g.*, lactic acid, betaine, and glucose).^33^ Green-innovative extraction such as microwave-assisted extraction (MAE), ultrasonic-assisted extraction (UAE), enzymatic-assisted extraction (EAE), and pressurized liquid extraction (PLE), constitute efficient alternatives [Fig. 1].^35^ The advantages and disadvantages of SPs extraction techniques are listed in Table 1.

**Table 1:** Advantages and disadvantages of SPs extraction techniques

Extraction techniques	Advantages	Disadvantages
Hot water extraction (HWE)	Easy to carry out, purity of the extracted SPs	Long extraction time
		Large volumes of solvents high temperatures
Microwave-assisted extraction (MAE)	Use of water instead of chemical solvents shorter operating time	High temperature can deteriorate thermolabile compounds
	Higher extraction efficiency	Inhomogeneous heating
	Extracted compounds possess good quality it utilizes directly fresh biomass from seaweed	
Ultrasound-assisted extraction (UAE)	Enhanced biomass digestion solvent consumption	UEA applications are still limited
	Higher purity	
	Lower energy consumption	
	Shorter operating time	
	Ability to achieve a larger yield of extracts efficient, environmentally friendly, low equipment expenses and maintenance, possibility to scale-up to industrial production, reduced number of process steps	
Pressurized liquid extraction (PLE)	Ability to obtain larger yield of extracts utilizing aqueous-based solvent	High temperature can deteriorate thermolabile compounds
	It has high extraction performance, less solvent usage, quick extraction time, and does not imply the use of hazardous solvents	High-pressure involved (safety issue), high-pressure power can bring depolymerization of compounds
Enzyme-assisted extraction (EAE)	Easy to carry out simple equipment	Strict temperature and PH
	Usually don't damage the SPs molecular structure	
	Ability to achieve a larger yield of compounds utilizing water	
	It is inexpensive, highly efficient, possibility to scale up, avoids the use of any harmful chemicals or organic solvents and it has a shorter extraction time	
	It preserves the structural integrity of the target compounds extracted that exert important bioactivities	

##### 2.2.2.1 Microwave-assisted extraction (MAE)

Microwave techniques are non-contact heat sources that generate heat energy *via* ionic conduction between a solvent and dissolved ions based on the use of electromagnetic radiation on a sample at wavelengths (1 mm to 1 m) and frequencies (0.3–300 GHz). Basically, uniform heating of the samples created increased pressure, causing the intracellular fluids to evaporate. Consequently, releasing polysaccharide molecules from cell walls into the solvent. MAE has been widely used in various fields such as chemistry, biology, and materials science due to its advantages of rapid heating, energy efficiency, reduced extraction duration, high extraction rate, good product quality, low cost, and easy operation. In addition, it is also a more environmentally friendly option due to its reduced solvent usage. Moreover, due to the high extraction rate, the yield of carrageenan from *Solieria chordalis* has been increased by 20%, showing that MAE is an efficient technique to extract SPs. However, the use of MAE can also cause some side effects, such as sample decomposition or degradation or non-uniform heating, if not properly controlled.^31–33^ Microwave-assisted extraction of fucoidan from the brown seaweed *F. vesiculosus* by Rodriguez. Extraction at 120 pressures for 1 minute with 1 g per 25 mL water demonstrated to be the best condition for maximal fucoidan recovery. It was determined that pressure, extraction time, and alga/water ratio all influenced SPs yield.^36^

##### 2.2.2.2 Ultrasonic-assisted extraction (UAE)

Ultrasound techniques propagate on samples as compression and rarefaction waves based on the use of ultrasonic waves above the audible frequency range (>20 kHz) and below microwave frequencies (≤10 MHz). The great amount of energy released by an ultrasonic wave as it travels through a solvent causes shock waves to form bubbles and zones of high and low pressure, increasing the surface area of contact between the liquid and solid phases. Asymmetrical bubbles are created in solid–liquid suspensions, which draw vapor from the solvent and expand and collapse, causing the breakdown of cell walls. UAE promotes cell wall disruption, mass transfer, improved penetration, an immiscible phase, and decreased particle size, thus optimizing yield and extraction efficiency. This leads to both higher compound quality and, since more molecules are extracted into the organic layer, quantity. It also decreases processing time and power consumption. All in all, these improvements will result in a cheaper and eco-friendly method for extracting and scaling industrial production. The UAE is more efficient compared to the conventional procedures in terms of carrageenan yield and purity.^31–33^ Carrageenan and alginates are water soluble functional polysaccharides of red seaweed and brown seaweed, respectively that were extracted with the aid of (UAE) making them highly biocompatible. As compared to the average extraction process where only 27% of the dry weight (DW) of seaweeds was extracted within two hours, thereby efficiently recovering SPs that represented up to 55% of the DW in a very short period that lasted between 15 and 30 min. The molar mass distribution and chemical properties of alginates and carrageenan were not affected by the UAE extraction. This indicates that UAE is a more efficient and time-saving method for the extraction of SPs from seaweed. Furthermore, the UAE mitigates the environmental implications of traditional extraction methods, which need substantial energy and produce significant waste.^33^ In the UAE, ultrasonic intensity, frequency, pressure, solvent viscosity, and liquid–solid ratio may influence its efficiency. Similarly, it also requires the process of extraction depending on conditions such as piston speed and time required for extraction. It is deemed essential to manage these effectively to ensure outcomes align with expectations while minimizing waste. Furthermore, the characteristics and quality of the solvent are critical factors influencing extraction methods.^32,33^

##### 2.2.2.3 Enzyme-assisted extraction (EAE)

The enzymatic extraction technique involves the use of digestive enzymes as catalysts to break the cell walls of the seaweeds, resulting in better release and more efficient extraction of bioactive content. The most used enzymatic treatments are cellulase, papain, trypsin, pectinase, glucosidase, gluconase, carbohydrases (*e.g.*, Viscozyme), and proteases (*e.g.*, Alcalaseare). These enzymes contribute to breaking down the physiochemical linkages between proteins and other molecules retained by the presence of hydrogen or hydrophobic interactions in the cells that were used to enhance the extraction yield. In addition, EAE has a multitude of advantages, namely nontoxicity, shorter extraction time, simplicity of operation, eco-friendliness, high efficiency, good product quality, low energy consumption, and high bioactivity because of the nature of enzymes. Moreover, the EAE extraction of SPs does not affect the chemical structure or the molar mass distribution. However, the high price of some types of enzymes limited the use of EAE extraction in the industry. In EAE, pH, substrate/enzyme ratio, relatively strict temperature, and type of solvent are some of the critical factors that need to be optimized for efficient extraction. Therefore, it is important to find the optimal conditions for each specific sample and enzyme combination.^31–33^

##### 2.2.2.4 Pressurized liquid extraction (PLE)

Pressurized liquid technique approach, the solvent is kept in a liquid state by maintaining temperatures above its boiling point. Most of the solvents are water or other solvents, either by themselves or as co-solvents in combination with other solvents like acids, deep eutectic solvents, or ionic liquids. The ideal conditions for PLE technique are 35 to 200 bar of pressure and 50 to 200 °C of temperature.^31–33^ PLE extraction provides significant advantages, such as reduced solvent consumption, improved separation efficiency, and lowered energy usage. However, the use of high temperatures may result in adverse responses such as detrimental reactions or material degradation. Therefore, precise temperature control is crucial during the PLE extraction.^31^

#### 2.2.3 General procedures of purification SPs

Purification techniques for crude extract SPs are an important step in enriching the desired compounds. Different techniques, such as physicochemical (precipitation, ultracentrifugation), membrane separation (dialysis, ultrafiltration), and chromatographic (gel permeation chromatography (GPC), ion-exchange chromatography (IEC), and size-exclusion chromatography (SEC)). Due to the presence of sulfate ions, SPs are negatively charged molecules, making anion-exchange chromatography an excellent method for removing neutral compounds. Size-exclusion chromatography also enables measurements of the total and molecular mass distributions. These techniques can be used individually or in combination to purify a wide range of biomolecules and eliminate proteins, monosaccharides, oligosaccharides, and other compounds from the crude extract SPs, including ethanol/salt precipitation.^27,35^

## 3. Sources of natural sulfated polysaccharides

### 3.1. Marine seaweed-glycans SPs

More than 70% of our planet is covered by various oceanic environments. Within the ecosystem of marine organisms, algae dominate the ultimate standard, comprising over 80% of the world's biomass.^3,37^ Algae are highly valued for their renewable nature, adaptability, compatibility with living organisms, sustainable sourcing, abundance, ease of cultivation, and wide variety of applications. Algae contain a wide range of bioactive molecules, including proteins, amino acids, polysaccharides, fatty acids, vitamins, minerals, dietary fiber, sterols, pigments, polyphenols, and more. Marine algae comprise significant quantities of sulfated polysaccharides (SPs), which are highly valuable in the field of biomedicine due to their various health benefits, such as anti-inflammatory, anticancer, anticoagulant, antibacterial, antithrombotic, antiviral, and immunomodulatory properties.^38^ The effectiveness of sulfated polysaccharides relies on factors such as the composition of the carbohydrate backbone, molecular weight, and, most importantly, the position and degree of sulfation.^10^ The algal source, life stage, growth environment, and extraction method all have an impact on the composition, structure, and rheological properties.^12^ Having sulfate groups on the polysaccharide structure leads to several significant chemical outcomes. The sulfate groups have negative charges that allow the binding to positively charged biomolecules across a wide pH range (4–12). Additionally, the sulfate groups coordinate water molecules to enhance and sustain tissue hydration.^8^ Three commonly used marine-based sulfated polysaccharides in biomedicine are carrageenan, fucoidan, and ulvan. They are derived from red, brown, and green algae, respectively.^37^

#### 3.1.1 Red seaweed

Red seaweed, scientifically classified as Rhodophyta, is a group of marine algae that is distinguished by its red or purple pigmentation. The distinctive characteristic is due to the existence of phycoerythrin and phycocyanin, which are both red and blue pigments, respectively.^12,39^ Red seaweeds have been used in food due to their rheological properties, such as their ability to gel and thicken. In contrast, carrageenan and agars have a wide range of applications including pharmaceutical and biotechnological applications as well as biological activities. Red seaweed is rich in polysaccharides, specifically floridean starch and sulfated galactans such as carrageenan or agaran. They make up approximately 40–50% of the dry weight of such algae.^10,12^ In accordance with their stereochemical characteristics, galactan can be divided into two main groups: agaran and carrageenan. Agaran, which contain 3,6-anhydrogalactose and d-galactose residues of the l-series; carrageenan, on the other hand, contain residues from the d-series.^10^

##### 3.1.1.1 Carrageenan

**Fig. 2:**
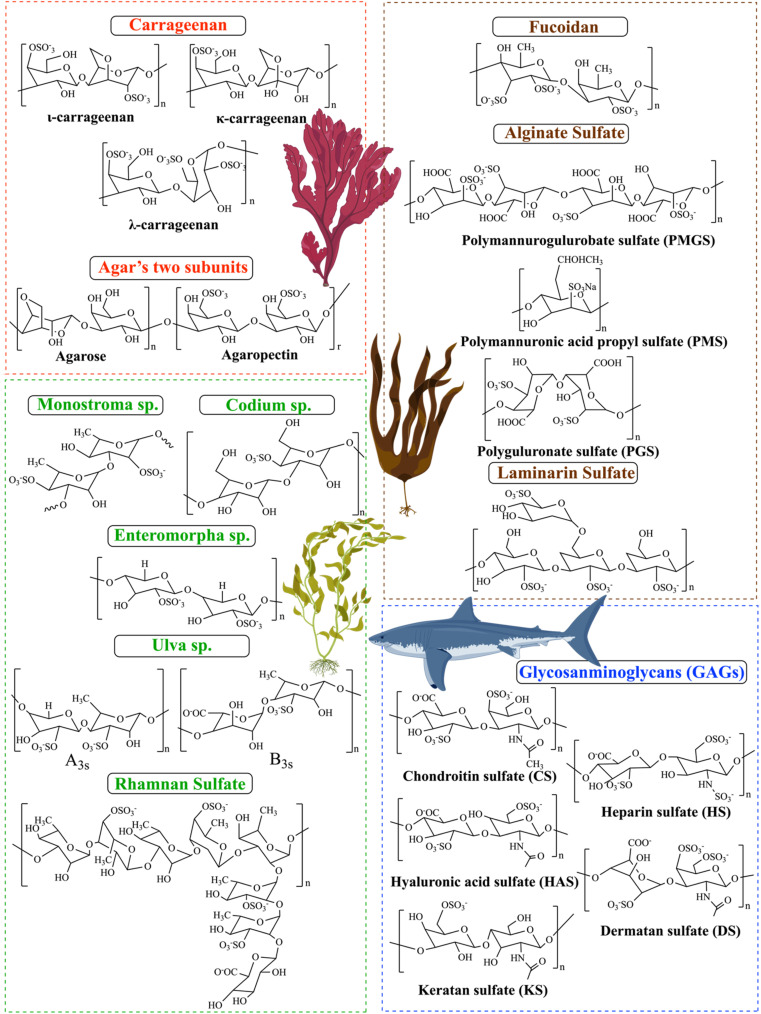
Chemical structure of various natural sulfated polysaccharides and modified SPs.

Carrageenan (CRG) has been used as a thickening, gelling, and stabilizer in food preparation. It was first introduced as a cough medication and gelatin in about 400 A. D. CRGs are a kind of linear SPs found in red seaweeds such as *Gracialaria*, *Gigartina*, *Gelidium*, *Lomentaria*, *Corallina*, *Champia*, *Solieria*, *Gyrodinium*, *Nemalion*, *Sphaerococcus*, *Boergeseniella*, *Sebdenia*, *Scinaia*, and others.^40^ CRG is a linear ester-sulfate polygalactan that is produced by red algae species extracted from the outer cell wall and internal matrix. Its structure contains approximately 15–40% ester-sulfate.^41^ CRG is water-soluble; however, the solubility of CRG can vary significantly depending on the circumstances. For instance, raising the temperature or changing the pH, medium ionic strength, or the presence of cations can greatly impact its solubility.^33^ The backbone of carrageenan consists of two alternative units, d-galactose and 3,6-anhydro-galactose, *via* α (1 → 3) and β (1 → 4) glycosidic linkages. The position and quantity of the sulfate groups, which are the ground structure, determine the activity and physicochemical properties of the carrageenan. Additionally, varying the sulfate group in quantity, distribution, and position are frequently distinguished into six categories: kappa (κ), iota (ι), lambda (λ), mu (μ), nu (ν), and theta (θ). Three of the most significant forms are κ-carrageenan, ι-carrageenan, and λ-carrageenan, as represented in [Fig. 2],^41–43^ especially since ι-CRG and κ-CRG exhibit gelling characteristics due to their ability to cross-link adjacent chains with their sulfate groups oriented outward to create organized 3D networks. Whereas, in λ-CRG, the sulfate group in the second position is oriented inward, which hinders cross-linking from forming. Gelling property is a crucial parameter that expands the range of applications by creating methods for controlling gelation and viscoelastic characteristics.^42^ Chemical cross-linking, mechanical strength, biological properties, and the sol–gel transition are all affected by variations in carrageenan's structure. Many industries rely on CRG for its distinctive properties, including the food, cosmetics, printing, textile, and medical industries. The antiviral capability of the molecule seems to be affected by the placement and density of the sulfate moieties on the backbone. This is a significant finding. That carrageenan's antiviral action depends on more than just its sulfate level is shown here. In addition, among sulfated polysaccharides, carrageenan has received the greatest amount of attention in human therapeutic studies aimed at treating various viral infections.^44^ In the field of medicine, CRG has been extensively studied, highly sulfated carrageenan functions similarly to heparin sulfate, which has been known to have coagulation-related effects. This suggests that carrageenan may have potential as an anticoagulant agent. Additionally, compared to the saline control, carrageenan treatment significantly decreased plasma cholesterol and lipid levels. The carrageenan group had a mean score of 1.88 compared to the saline control group's 3.84 (a scale of 0–5, with 0 representing no lesion formation and 5 being severe lesion formation).^41^

##### 3.1.1.2 Kappa-(κ-)carrageenan (κ-CRG)

κ-CRG has one sulfate for every repeating unit of a disaccharide [Fig. 2] compared to ι-CRG, the former is more effective at creating hydrogels, which contributes to its high hydrogel-forming efficiency and makes it a popular ingredient in various industries.^45^ κ-CRG hydrogels in combination with stem cells and growth factors (GFs) have emerged as a promising strategy to approach cartilage regeneration. According to Rocha *et al.*, cells and the transforming growth factor-b1 (TGF-b1) were both enclosed in hydrogels made of κ-CRG. The hASCs' ability to differentiate into cartilage was improved by the addition of TGF-b1 to the hydrogel made of κ-carrageenan due to their thixotropic gelling and thermoreversible characteristics of κ-CRG under physiological conditions. These results suggest that the injectable thermoresponsive formulation applications for this new cartilage tissue engineering (TE) are very promising.^46^ New biomaterials for bone tissue engineering were obtained by κ-CAR blended into biodegradable polyesters to create a biocompatible scaffold. The presence of κ-CAR could enhance the fiber of polyhydroxybutyrate (PHB) and polyhydroxybutyrate valerate (PHBV) and improve the mechanical properties of the scaffold, as evidenced by the study by Goonoo *et al.* Different levels of miscibility were produced by the electrospun PHB/κ-CRG and PHBV/κ-CRG fibers, which in turn affected the fiber morphology, and surface characteristics, and allowed for customized degradability. These materials showed promising results *in vitro*, indicating their potential for use as scaffold materials in bone tissue engineering applications.^47^ Sun *et al.* studied the relationship between the molecular weights of κ-CRG and antioxidative activity to investigate the effect of the molecular weight κ-CRG of different molecular weights prepared by oxidative modification and evaluated against superoxide anions and hydroxyl radicals. The hydroxyl groups in the low-molecular-weight κ-CRG backbone exhibit antioxidant activity, allowing them to react with superoxide anions (highly toxic species that are generated by numerous biological and photochemical reactions) and hydroxyl radicals through hydrogen bonds. The hydroxyl groups take the place of the depleted sulfate groups during the mechanism of degradation, and increasing the quantity of hydroxyl groups in the modified products makes it a great candidate for the potential of modified-κ-CRG as an antioxidant and suggests that it might be a natural source of antioxidants.^48^

##### 3.1.1.3 Iota-(ι-)carrageenan (ι-CRG)

ι-CRG has two sulfate groups for every repeating unit of a disaccharide [Fig. 2].^45^ The nasal spray version of iota-carrageenan has already been shown to be secure and efficient against viral upper respiratory infections such as coronavirus, the common cold, human rhinovirus, and influenza A H1N1. The primary mechanism by which iota-carrageenan inhibits antiviral activity is through its interaction with viral particle surfaces, preventing viral particles from entering cells and trapping viral particles released from infected cells. Varese *et al.* studied the comparison of the effectiveness of sodium chloride and iota-carrageenan against SARS-CoV-2. Iota-carrageenan significantly reduces SARS-CoV-2 production in a dose-dependent manner. According to the results, 394 individuals were given either iota-carrageenan or placebo at random. Subjects who received the iota-carrageenan nasal spray (2 of 196 [1.0%]) and those who received a placebo (10 of 198 [5.0%]) experienced significantly different rates of COVID-19. The relative risk of getting sick was reduced by 79.8%, and the absolute risk was reduced by 4% when using the iota-carrageenan spray. Clinical use of the treatment successfully prevented SARS-CoV-2 infection in human respiratory epithelial cell line culture, supporting the theory that iota-carrageenan may be a promising candidate for the prevention of COVID-19.^49^ Sulfate groups can enhance the binding of different biologically active proteins, which results in anticoagulant activity. Carrageenan's sulfate content and high molecular weight can both impact its anticoagulant activity. For instance, ι-CRG has proven to have anticoagulant properties that are three times stronger than κ-CRG. Therefore, the type of carrageenan used can greatly affect its effectiveness as an anticoagulant.^50^

##### 3.1.1.4 Lambda-(λ-)carrageenan (λ-CRG)

λ-CRG has three sulfate groups for every repeating unit of a disaccharide [Fig. 2].^45^ Compared to κ-CRG/ι-CRG, λ-CRG exhibited more inhibitory behavior toward drug-resistant viruses. The antiviral activity of carrageenan is explained by its mechanism: λ-CRG binds to specific areas on the cell surface, preventing the virus from attaching to the cell and protecting it from the virus. λ-CRG also exhibits antitumor properties and has few side effects. The molecular weight has a significant impact on the inhibitory activity of carrageenan against tumor growth.^50^ The biological activities of carrageenan, which had the highest level of sulfation, included anti-tumor, anti-viral, antioxidant, anti-proliferation, and anti-viral. Additionally, carrageenan has demonstrated a successful adjuvant effect in therapeutic and preventative vaccines for cancer treatment. According to Jazzara *et al.*, carrageenan has the biological effect of decreasing the growth of MDA-MB-231 breast cancer cells and inducing apoptosis. The results indicated that carrageenan was a potentially effective agent that might be used to treat or prevent breast cancer. Furthermore, it has been found that carrageenan has immunomodulatory properties, which can enhance the immune system's response to different tumor cells. This suggests that carrageenan may have a promising role in cancer immunotherapy.^51^ Low-molecular-weight λ-CRG appears to be a more promising anticancer agent compared to high-molecular-weight λ-CRG. According to Tiasto *et al.*, λ-CRG significantly reduced cell viability. λ-CRG inhibited cell cycle progression in the S phase of FLO-1 and G1 in KYSE-30 esophageal cell lines, and significant reduction in the proteins Cyclin E, CDK2, and E2F2 followed λ-CRG treatment. Additionally, human colon RKO underwent selective apoptosis when exposed to λ-CRG. It has been demonstrated that λ-CRG can decrease Cyclin E expression. Following treatment with λ-CRG, the expression of cyclin-dependent kinase-2 was also significantly reduced. These results imply that λ-CRG could potentially be used as a colon cancer therapeutic agent.^52^

##### 3.1.1.5 Agar

The red algae of the ocean (*Gelidium* and *Gracilaria*) are the source of the water-soluble polysaccharide known as agar. The use of agar as a gelling and thickening ingredient in food dates to 300 A. D. A freeze–thaw method was created in 1958 by the Japanese. It allowed for the medicinal extraction of agar from water extracts.^40^ Agar consists of two subunits, agarose, and agaropectin [Fig. 2]. Agarose is a natural gelling polysaccharide (approximately 70% of the total), whereas agaropectin is a sulfated nongelling polysaccharide that has thickening characteristics.^53^ A monosaccharide residue in agaropectin, a derivative of agarose, is substituted to varying degrees by sulfated groups, pyruvate groups, and methoxys. The composition of the mixture determines the structure and characteristics of the agar. The chemical structure of agar is made up of two alternating disaccharides, namely 3, 6-anhydro-l-galactose, and d-galactose units linked by α (1,3) and β (1,4) glycosidic bonds.^12,54,55^ Agarose is composed of three linked β-d-galactose and four linked 3,6-anhydro-α-l-galactose with very few hydroxyls being sulfate. Agaropectin is an acid polysaccharide consisting of d-glucuronic, pyruvic acid, and sulfate ester groups conjugated to agarobiose.^55^ In addition, The agar properties are dependent on the amount and position of the sulfate groups can affect the physicochemical and biological properties of agar, such as the gelation properties.^12^ Agar is one of the most interesting polysaccharides due to its biodegradable biofilm properties.^56^ Furthermore, agar has been widely used in microbiology as a solidifying agent for culture media due to its ability to form a gel at relatively low concentrations. Additionally, agarose has potential applications in pharmaceutical, cosmetic, and medicine due to its unique gelling properties and its high mechanical strength.^41^ This material is helpful in cell culture and other microbiological experiments because of its gelation properties. Numerous studies using these materials in tissue engineering have been published recently because of their thermoreversible qualities.^12^ Agaropectin and agarose have similar backbone structures, which makes them directly linked together, which makes them resistant from broken down enzymatically by the bacterial species. Since agar has a dietary fiber property, numerous studies have looked at how agar affects cholesterol and lipids.^53^ According to Qi *et al.* agaropectin, the highest sulfated agar, could successfully extend the coagulation time *in vitro* in a dose-dependent manner. Also, *in vivo* rabbit blood was treated orally with agaropectin from *Gelidium amansii*, and the prolongation of the PT and TT shows that heparin and *Gelidium amansii* agaropectin have comparable anticoagulation mechanisms. This suggests that *Gelidium amansii* agaropectin has the potential as a natural anticoagulant.^57^ Agar is an appealing candidate for drug delivery because of its biodegradable nature. Varshosaz *et al.* studied *in vivo* the effectiveness of the designed nanospheres in the pulmonary biomembranes route for the delivery of bupropion, an atypical antidepressant drug. Drug loading effectiveness was 38.6%, and drug release effectiveness was 51% for approximately 5 hours. The nanospheres displayed strong bioadhesives. This suggests that bupropion delivery *via* nanospheres may offer promise for this delivery method's long-term efficacy and saftey.^55^

#### 3.1.2 Brown seaweed

Brown seaweeds: scientifically categorized as Phaeophyceae, are a group of marine macroalgae that have a distinct brownish, yellow-brown, or red-brown pigmentation. The distinctive feature is attributed to the presence of fucoxanthin.^10,39^ Brown seaweed cell walls are composed of sulfated polysaccharides such as laminarin, alginate, and fucoidan. The unique physical and chemical properties of these three species of brown algae make them very promising candidates for use in a wide range of biological applications. They have a wide range of possible medical applications, including the therapy of arteriosclerosis, rheumatic processes, hypertension, goitre, asthma, ulcers, menstrual disorders, syphilis, ulcerative colitis, and many more.^38^

##### 3.1.2.1 Fucoidan

Fucans are the most prevalent sulfated polysaccharides; fucoidan, which comes from Fucans is a well-known example.^28^ Fucoidan, referred to as “sulfated fucan” and “fucosan,” It is present in some marine invertebrates, such as sea urchins and sea cucumbers, as well as in brown seaweeds;^58^ however, brown algae generate a greater quantity and possess more bioactive fucoidan. The backbone mostly consists of substantial amounts of l-fucose, sulfated ester groups, and minor quantities of monosaccharides such as xylose, glucuronic acid, galactose, and mannose, but brown algae provide a higher yield and more bioactive enhanced fucoidan. Its backbone is primarily made up of large quantities of l-fucose, sulfated ester groups, and small quantities of monosaccharides like xylose, glucuronic acid, galactose, and mannose.^28,31^ Fucose constitutes around 40% w/v of the total monosaccharides in fucoidan, whereas in some species, this percentage may ascend to 80% w/v.^31^ The chemical content varies based on the species of seaweed, its heterogeneity, and the extraction procedures used. This diversity may also influence its bioactive characteristics and prospective uses.^28^ Fucoidans are categorized into two categories based on their backbone structure: Type I and Type II. Type I fucoidan, recovered from *Sargassum* and *Fucus* species, demonstrated that the linear backbone comprises successively linked α-(1 → 3) and α-(1 → 4) l-fucopyranose residues, with sulfate groups located at the O-2, O-3, and O-4 positions of fucose. Type II fucoidan found mostly in Laminariales, differs from other sulfated polysaccharides owing to its unique backbone structure, which consists of alternating α-(1 → 3) linked l-fucopyranose with sulfate groups at the O-2 and O-4 positions of fucose residues [Fig. 2].^31,34,38,58^ This unique structure provides anti-inflammatory, antioxidant, anticoagulant, antitumor, and antiviral properties. It's interesting to note that, fucoidan's growth factor (TGF)-β1-binding abilities, which are relevant to its heparin-like anticoagulant and antithrombotic agent, also, were used for cartilage tissue engineering applications, can also be used as a functional additive for creating new drug delivery systems due to its non-toxicity and biodegradability. This opens new possibilities for the use of fucoidan in the pharmaceutical industry.^37,38^ Fucoidans have been shown to have anticancer and antimetastatic effects on cells with a variety of histogenesis, including human lung, breast, hepatic, colon, prostate, and bladder cancer cells. Anisimova *et al.* used the model of capillary-like structures forming in the 3D culture of the cancer cells. After the MDA-MB-231 cells were incubated to investigate the potential of fucoidan as an anti-angiogenic agent *in vitro*. The MDA-MB-231 line of low-grade human breast cancer cells and canine multipotent mesenchymal stem cells (MSCs) were used to test the effectiveness of the investigated compounds. Data show that fucoidans and their derivatives have significantly increased anticancer activity.^59^ According to Jin *et al.*, fucoidans had two fractions in which both SJ-I and SJ-GX-3 were able to significantly reduce tau uptake in the cells, which exhibited a stronger binding affinity to tau compared to heparin. These findings suggest that fucoidans may have potential therapeutic applications for the treatment of Alzheimer's disease (AD).^60^

#### 3.1.3 Green seaweed

Green seaweed, the most dominant species of Chlorophyceae, is a form of marine macroalgae characterized by its distinctive green pigmentation.^10^ This unique characteristic arises from the existence of chlorophyll and other pigments. Characterized by the polysaccharide compositions originating from *M. lattisimum* (rhamnan sulfate) and *U. meridionalis* (ulvan). These seaweeds are rich in special hydrocolloids which are mostly composed of glucuronic and rhamnose acids, with a high concentration of sulfate groups bound to the rhamnose molecules.^42^ The green alga *Monostroma nitidum* is completely different from *Ulva* Linnaeus (sea lettuce). Despite this, *M. nitidum* and sea lettuce are classified into distinct biological groupings. *M. nitidum* has a singular layer of cell assemblies, while species of sea lettuce often exhibit two layers of cells.^39^ Although red macroalgae are the predominant source of sulfated galactans, some green algae species, such as *Codium*, also contribute significantly to the provision of these compounds.^61–63^ Galactans derived from green algae tend to be represented by a higher degree of complexity and structural heterogeneity compared to those obtained from red algae. As an example, *C. fragile* and *C. cylindricum* contain sulfated arabinogalactan and sulfated glucogalactan, respectively.^62^

##### 3.1.3.1 Ulvan

Among the several sulfated polysaccharides found in green algae, the most numerous of them is ulvan, which is found in the cell walls of *Enteromorpha*, *Gayralia*, *Codium*, *Caulerpa*, and *Monostroma*. About 8–29% of the dry weight of algal biomass is constituted by ulvans, which are structures made of disaccharide repeating moieties that include sulfated rhamnose linked to glucuronic acid, iduronic acid, or xylose.^40^ Ulvan has been demonstrated to have anticoagulant, antibacterial, antiviral, and immunomodulatory properties in both *in vitro* and *in vivo* studies. Several low-molecular-weight ulvan isoforms (ULVAN-F1, ULVAN-F2, and ULVAN-F3) isolated from *Ulva pertusa* were shown to be efficient in suppressing vesicular stomatitis virus infection and reproduction [Fig. 2]. However, the antiviral efficacy of ulvan is not consistently correlated with its molecular weight. SU1F1 exerts its antiviral effects mostly by decreasing DNA replication and transcription, concurrently lowering HSV protein synthesis. The ulvan-containing polysaccharide extract inhibits the adsorption and viral penetration of the Japanese encephalitis virus (JEV) into host cells. The bioactivities of ulvan may be influenced by its molecular weight. Y. Chi *et al.* investigated two variants of ulvan extracted from *Ulva pertusa*. One contained a solitary GlcA residue (1068.2 kDa), while the other was an elongated branch ulvan-F1 (38.5 kDa), with a partial composition of GlcA-Glc. The vesicular stomatitis virus infection and replication may be considerably reduced by 100 μg per mL ulvan-F0 and ulvan-F1, according to the antiviral experiment. The inhibition rates of VSV replication were 40.75% and 40.13%, respectively.^64^

##### 3.1.3.2 Rhamnan sulfate

Rhamnan sulfate is generally composed of l-rhamnose linked *via* α-1,3 carbons. The main chain of 1,3-linked α-l-rhamnose units generates over the chemical structure of rhamnan sulfate, or octa-saccharide repeating units [Fig. 2], which were extracted from the green seaweed *M. nitidum*. Approximately 25% of these units have partially sulfate groups substituted at the C-2 position on the main chain, as well as at the C-4 position of the l-rhamnose units on the main chain, and the C-3 position on the side chains.^65,66^ According to study data, the aPTT assay for *in vitro* anticoagulant activity demonstrates that at low molecular weight form of rhamnan sulfate possesses greater anticoagulant activity compare with heparin at high concentrations.^65^

### 3.2. Animal-derived SPs

#### 3.2.1 Glycosaminoglycans (GAGs)

Animal-derived polysaccharides demonstrate noteworthy impacts within the field of biomedical science.^67^ Heteropolysaccharides are present in the extracellular matrix (ECM) of higher organisms, either in free-standing form or coupled to proteins to form proteoglycans. This ability to interact with various proteins play a significant role in affecting their function and influencing important biological. Glycosaminoglycans are anionic, linear heteropolysaccharides composed of repeating disaccharide units linked together by glycosidic bonds. They possess anionic character because of the sulfate groups present in their structure. There are two groups of naturally occurring GAGs: sulfated and nonsulfated. Heparin, chondroitin sulfate (CS), keratan sulfate, dermatan sulfate (DS), and heparan sulfate (HS) are all examples of sulfated glycosaminoglycans (GAGs).^45,67,68^ Hyaluronic acid (HA) is a nonsulfated glycosaminoglycan (GAG). The repeating units contain uronic acid, namely either d-glucuronic acid or l-iduronic acid, as well as an amino sugar, either galactosamine or glucosamine; yet, sulfated hyaluronic acid may be synthesized by the chemical modification at various oxygen and nitrogen positions in their composition. They vary according to the chain length, the connection with proteins, the degree of sulfation, and the ratio of uronic acid, whether they contain hexose, hexosamine, or hexuronic acid in their structure. CS and DS are classified as galactosamino-glycans due to their inclusion of galactosamine. HS and heparin contain glucosamine and are hence classified as glucosaminoglycans.^45^ Among them, HA and CS are significant materials that can be derived from various components (*e.g.*, cartilage, bones, skin, head, heart, and fins) of numerous marine organisms (*e.g.*, whales, sharks, rays, salmon) and have applications in diverse fields including biomedical, cosmetic, food, and pharmaceutical sectors.^69,70^ Dermatan sulfates are stereoisomers of chondroitin sulfate found in the skin, blood vessels, tendons, and lungs. Keratan sulfates consist of repeated disaccharide units of galactose and *N*-acetylglucosamine linked by β 1,4 and β 1,3 connections and are often located in osseous cartilage and the cornea.^5,28^

##### 3.2.1.1 Heparin and heparan sulfate

Heparin, a well-defined sulphated polysaccharide discovered in 1916, was first used clinically nearly twenty years after its discovery.^8^ Heparin is a naturally occurring glycosaminoglycan with a linear structure that is highly sulfated. It is made up of repeating monomer units of sulfonated hexuronic acid (1 → 4) d-glucosamine. The remaining portion of uronic acid in heparin is composed of either α-l-iduronic acid (IdoA) or β-d-glucuronic acid (GlcA) [Fig. 2]. As a well-established pharmaceutical, heparin plays a role in a wide range of physiological and pathological activities, such as angiogenesis, inflammation, cell adhesion, proliferation, and anticoagulation. Multiple studies have shown that heparin can regulate various biological processes by interacting with the basic amino acid groups of proteins. This includes binding with growth factors, building a complex to stabilize them, and extending their functional lifespan. Heparin's primary role is to act as an anticoagulant, achieved by interacting with the serine protease inhibitor antithrombin III. Antithrombin is the main inhibitor of blood clotting proteinases. When antithrombin binds to soluble heparin or heparan sulfate in the vascular wall, it quickly inhibits thrombin and other activated coagulation factors especially Xa and Ixa.^45,67,68^

##### 3.2.1.2 Chondroitin sulfate (CS)

The main sulfated glycosaminoglycan (GAG) formed from the amino sugar galactosamine is chondroitin sulfate (CS).^37,71^ CS is abundantly found in various tissues such as human and animal cartilage, tendon, ligament, cornea, and vascular walls. However, cartilage is the primary source of CS. Chondroitin sulfate is a linear polysaccharide consisting of repeated disaccharide units of *N*-acetyl-d-galactosamine and d-glucuronic acid. These units are connected by β 1, 4 and β 1, 3 linkages [Fig. 2].^28,45,69^ In general, sulfation occurs at either the C-4 or C-6 position on the galactosamine molecule, and at the C-2 position on the glucuronic acid molecule.^37^ Furthermore, CS can be categorized into many groups, including A, B, C, D, and E, based on the location of the sulfate group replacement.^69,70^ CS displays remarkable physicochemical and biological properties. CS can form electrostatic contacts with positively charged groups for drug delivery, due to its negative surface charge. The negatively charged surface of CS makes it harder for plasma components to bind to it. This means that medicine stays in the bloodstream longer, which increases its biological half-life.^3^ The capacity to absorb large amounts of water is another way in which CS improves tissue hydration. On top of that, CS is involved in many important biological processes and has anti-inflammatory characteristics.^70^

## 4. Modification of polysaccharides

**Scheme 1:**
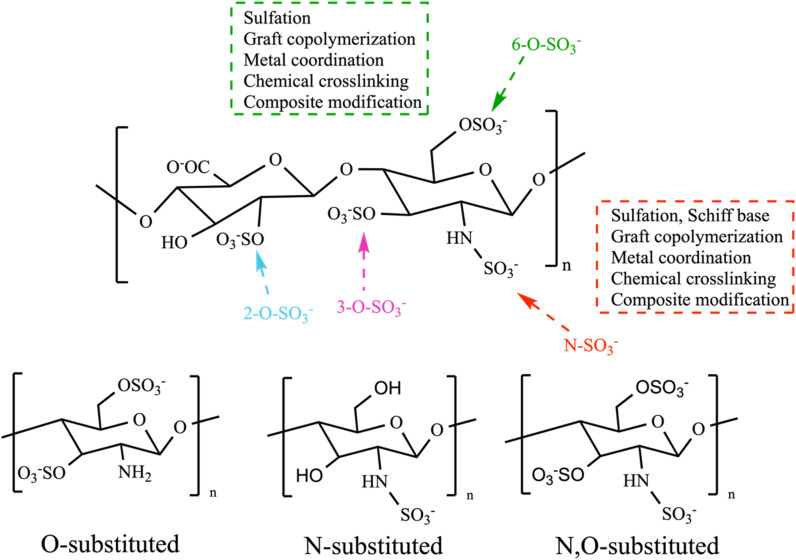
Overall modification positions and substitution positions.

As the prevalence of diseases such as cancer, heart disease, and COVID-19 continues to rise, there is a growing demand for the creation of effective medications to fight them. Polysaccharides have become highly valued in the treatment of these diseases due to their numerous advantages, such as their biocompatibility, abundance, sustainability and most importantly their biological properties. Furthermore, their non-toxic nature may help reduce the lingering side effects often associated with synthetic drugs.^72^ Polysaccharides derived from natural sources possess inherent limitations, such as the limited solubility of chitosan and the excessive hydrophilicity of cellulose. These drawbacks might hinder the overall utilization of polysaccharides in various biomedical domains. In addition, certain natural polysaccharides possess limited biological activity.^12^ Therefore, it was necessary to improve polysaccharides to meet the demand for the development of medical care. The modification methods and conditions have a significant impact on the molecular weight, linkages of monosaccharides, conformation, solubility, and types, degrees, and positions of the substituent groups of SPs. As a result, these factors play a crucial role in determining the physicochemical and biological properties of SPs.^31,72,73^ Certain polysaccharides undergo additional modifications through the introduction of new functional groups, resulting in the inheritance of unique characteristics. There are several ways to modify polysaccharides, such as physical, chemical, and biological methods, or combination of these techniques. Physical methods involve using heat, microwave radiation, ultrasonic waves, high-pressure techniques, and other similar approaches. Biological processes use microorganisms or enzymes to break down polysaccharides through catalysis, which is a highly efficient and eco-friendly technique. However, the application of this type of modification is currently restricted to the degradation of specific types of SPs. Currently, chemical modification is the predominant technique employed to introduce novel biological activities by altering functional groups and enhancing mechanical and chemical properties, biocompatibility, solubility, control of biodegradability, and manufacturing capabilities.^29,31^ Numerous native polysaccharides have recently undergone modifications through common methods to create novel derivatives of polysaccharides. These modifications include sulfation, acetylation, phosphorylation, carboxymethylation, amination, benzylation, *C*-glycosylation, hydroxypropylation, selenylation, etherification, esterification, oxidation, graft polymerization, and more.^12,31,72^ Chemical modifications such as grafting, cross-linking, complexation, covalent coupling, and composite formation offer additional possibilities for designing advanced materials.^30^ The hydroxyl groups (–OH) are the most extensively studied and chemically altered functional groups in polysaccharides. However, other functional groups such as amino (–NH_2_), carboxylic acid groups (–COOH), and aldehydes (–CHO) have also been utilized for chemical reactions [Scheme 1]. When introducing acidic, basic, hydrophilic, hydrophobic, or other molecules with specific properties, the structure of polysaccharides can be modified without fundamentally altering the polysaccharide backbone. Nevertheless, it will enable the implementation of advanced modifications necessary for specific applications, ultimately altering the final properties of the developed biomaterials.^70^ This review focuses on a common method of modification known as sulfation, which is used as a simplified example to clarify our understanding of the fundamental principles behind chemical modifications of polysaccharides.^12^

### 4.1. Chemically SPs

#### 4.1.1 Sulfation

Polysaccharides consist of numerous monosaccharide molecules connected by glycosidic linkages. Chemical sulfation is a process that uses a sulfate donor, typically an acid, in a polar aprotic solvent. This reaction is carried out in a one-step reaction. By controlling reaction conditions, it is possible to achieve consistent adjustment of the DS (degree of sulfate groups connecting to hydroxyl groups on SPs) while also reducing depolymerization and other unwanted side reactions. It is crucial to note that polysaccharides differ in their ability to dissolve and react, and hence, specific procedures may be necessary for modifying them chemically.^5,31^ Chemical modifications can be made to the hydroxyl groups in the chain of polysaccharides through sulfation reactions. Sulfation can be defined as the addition of sulfate groups to the carbon chains of polysaccharides. These sulfate groups typically replace the hydroxyl groups connected to specific carbon positions (C-2, C-4, or C-6) of the polysaccharide backbone by an esterification reaction.^31,61,72^ According to reports, the hydroxyl groups of monosaccharide residues at C-6 are more active, generally more accessible, and easier to substitute compared to other hydroxyl groups. This is because they experience weak steric hindrance and hydrogen bonding interactions, making them more prone to substitution with sulfation and other large functional groups. It is important to note that sulfated polysaccharides are anionic polymers that do not affect pH, unlike carboxylated polysaccharides. In fact, sulfated polysaccharides could increase the number of reactive sites on the polysaccharide chain.^8^ Extensive research is currently being conducted to synthesize sulfated polysaccharides by gradually adding sugars and to establish relationships between their structure, properties, and activities. This is due to the fascinating and varied biological effects exhibited by these compounds. Nevertheless, the process of creating sulfated polysaccharides poses significant difficulties. These challenges arise from the multitude of stereocenters, the similarity of functional groups, and the requirement to maintain the proper orientation of glycosidic linkages. Generating polymers of varying molecular weights, both tiny and large, while maintaining a restricted polydispersity, presents an extra difficulty. Thus, in terms of chemical synthesis, polymeric sulfated structures are acquired by sulfating either natural polysaccharides or polymeric analogues of polysaccharides. These post-polymerization modification methods are highly efficient and yield abundant material for biological testing and comparative studies with naturally occurring sulphated polysaccharides.^8^ The initial documented techniques for the unspecific sulfation of polysaccharides involved the use of sulfuric acid (H_2_SO_4_) or its derivatives, such as chlorosulfonic acid (ClSO_3_H) or sulfamic acid (H_3_NSO_3_). These methods are still widely utilized because they are straight forward, can be easily scaled up, and require affordable reagents. An early instance of chemically sulfated polysaccharides was obtained by breaking down and refining cellulose fibers using H_2_SO_4_, which led to the creation of cellulose sulfate esters.^5^ Various reagents can be employed for sulfation of polysaccharides, such as acid hydrolysis using HCl or H_2_SO_4_ (sulfuric acid, sulfur trioxide-pyridine, chlorosulfonic acid-pyridine, and sulfur trioxide dimethylacetamide), alkaline hydrolysis (using aqueous 0.5 M NaOH), and oxidation with Fe–H_2_O_2_.^7,12,27^ The sulfur trioxide-pyridine technique is commonly preferred due to its high efficiency and easy procedures.^12^ The primary disadvantages of employing chemical procedures are the utilization of potent acids or bases and elevated temperatures, both of which constitute harsh conditions for the reaction to take place. Under these conditions, polysaccharide degradation may occur. Linkages between uronic acid residues exhibit remarkable stability at lower pH values. Nevertheless, the connections between neutral sugars may be vulnerable to the effects of acid, leading to a rapid breakdown of sidechains into smaller molecules known as oligomers and monomers.^5,74^ Upon modification, the physical properties and biological activity of the sulfated polysaccharide undergo alterations.^61^

##### 4.1.1.1 Chloro-sulfate pyridine (ClSO_3_-Py) method

**Scheme 2:**
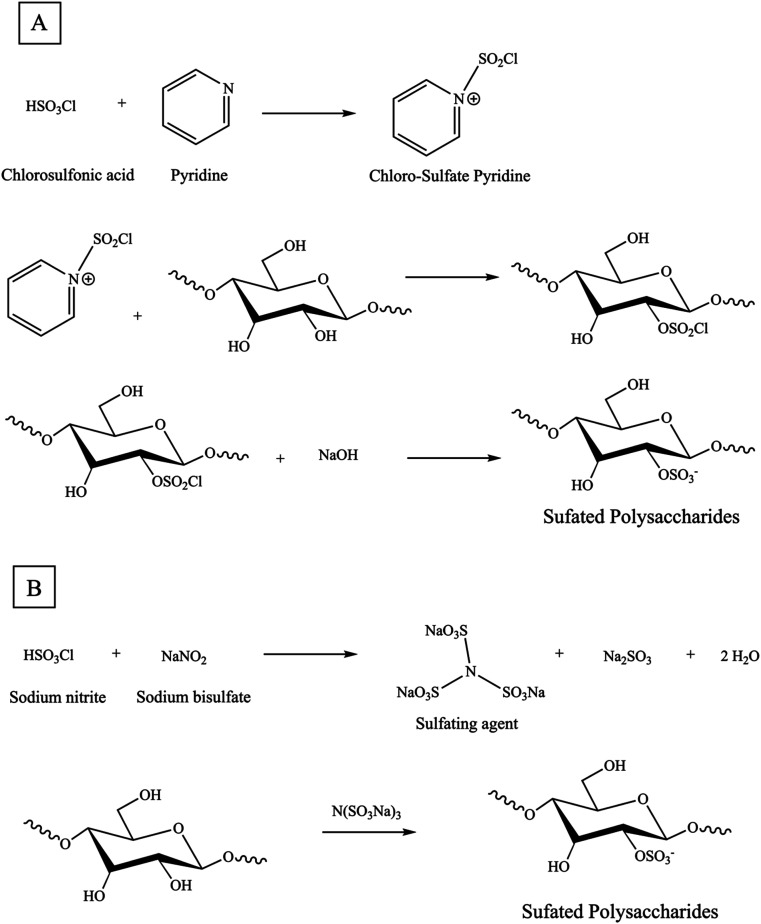
(A) Synthesis reaction mechanism of sulfation of a glucose (Glc)-based polysaccharide with chloro-sulfate pyridine method, (B) novel green sulfation method.

The chlorosulfonic acid-pyridine approach is widely employed for sulfation modification of polysaccharides. The first step of sulfation reactions involves employing chlorosulfonic acid and pyridine as a sulfation reagent. Chlorosulfonic acid is added dropwise to pyridine while continuously stirring in an ice bath [Scheme 2A]. Pyridine serves as a catalyst for the sulfation reaction and perhaps eliminates degradation and other undesirable side reactions that may occur when heating with strong acids alone. Additionally, the pyridine, a potent organic base, can act as a nucleophile and attack the polysaccharide, causing the H–O bond to weaken and allowing for the entry of the sulfate group. Moreover, pyridine acts as an aprotic agent to ensure a uniform reaction mixture. Regard to pyridine, dimethyl sulfoxide, formamide, and dimethyl-formamide have been reported as alternative solvents for the sulfation of polysaccharides.^5,62,72^ Furthermore, in the substitution reaction, the polysaccharide is dissolved in a precise quantity of N–N dimethylformamide or formamide. Subsequently, it is gradually introduced into the resulting adhesive white sulfation reagent.^62^ Sulfation reactions are typically conducted at a temperature of 45 °C for a period of 6 hours or at 60 °C for 15 minutes. Afterward, the reaction mixture produced during the manufacture of the sulfating agent is neutralized using NaOH.^72^ The results of this approach showed a slightly lower percentage yield of 115% w/w and degrees of substitution of 3.27% sulfur content or 0.19 DS, compared to the sulfur trioxide pyridine method.^8^ Three primary criteria that influenced the degree of substitution were the percentage of reagents, reaction time, and temperature. The variation in the DS also results in distinct biological functionalities. The orthogonal experiment and response surface method are commonly employed to identify the ideal sulfating conditions for producing highly active sulfated derivatives.^44^

##### 4.1.1.2 Sulfur trioxide pyridine (SO_3_-Py) method

The sulfur trioxide-pyridine approach is very similar to the chlorosulfonic acid-pyridine approach, with the only difference being the substitution of chlorosulfate in the sulfation reagent with the sulfur trioxide molecule. The approach has no limitations imposed by strict temperature and time considerations. Once the pH is adjusted to a neutral level, the liquid portion is subjected to dialysis and subsequently freeze-dried to obtain a solid form of sulfated polysaccharide. In comparison to the chloro-sulfate pyridine approach, the use of sulfur trioxide offers a less intense reaction process and facilitates the production of highly substituted sulfated polysaccharides, making it a potentially favorable method. Nevertheless, the high cost of the chemicals may restrict the widespread use of the sulfur trioxide pyridine approach.^61^

##### 4.1.1.3 Concentrated sulfuric acid (H_2_SO_4cons_) method

The concentrated sulfuric acid approach involves two primary types of reactions: the first one is a mixture of concentrated sulfuric acid and pyridine. While the other one, involves a mixture of concentrated sulfuric acid, *n*-butanol, and ammonium sulfate as the reaction medium. In this reaction, polysaccharide powder can be immediately added in the sulfuric acid reaction and pH is maintained at a neutral level throughout this process. Comparing to the chloro-sulfate pyridine approach, the sulfuric acid approach offers more stable reaction conditions with less strictly conditions in time and temperature. The modification using sulfuric acid approach in polysaccharide is limited compared to the previous two procedures, due to the serious dehydrating properties and highly acidic nature of sulfuric acid can lead to the carbonization of polysaccharides and the destruction of sugar chains. These factors have a significant impact on both the yield and the effectiveness of modifying sulfated polysaccharides.^44,61^

##### 4.1.1.4 Novel sulfated method

In past centuries, sulfating agents most frequently included sulfuric acid, chlorosulfonic acid, sulfuryl chloride, sulfur trioxide, and sulfamic acid. A variety of organic solvents, including pyridine, dimethyl sulfoxide, and formamide, have been applied as reaction mediums. However, these reagents can cause significant hydrolytic breakdown of the reactant and pose substantial environmental pollution issues. Thus, in contrast to conventional techniques, the novel sulfating agent N(SO_3_Na)_3_ enables the entire reaction to occur in an aqueous solution. It possesses several advantageous characteristics, including non-toxicity, affordability, minimal pollution, and, most significantly, the ability to prevent the hydrolysis or breakdown of sugar chains. In the reaction vessel, a certain quantity of sodium bisulfate was dissolved in distilled water. Next, the sodium nitrite, which had been previously dissolved in distilled water, was slowly added to the reaction vessel while stirring magnetically and maintaining a reflux temperature of 90 °C. The reaction was allowed to proceed for 1.5 hours. The sulfating agent N(SO_3_Na)_3_ was produced using this method. Next, the sulfating agent solution was modified to an appropriate pH by adding sodium hydroxide. After that, polysaccharide was introduced into the solution mentioned earlier while being stirred magnetically. The reaction was then allowed to continue for a specific duration at a predetermined temperature [Scheme 2B].^75^

#### 4.1.2 Cellulose and its sulfated derivatives

The most prevalent natural polysaccharide is cellulose (CL), which is also the principal component of plant cell walls. Because it is a plant-based substance, CL is safe for humans, animals, and the environment; it is also renewable and biodegradable. These days, every study aiming to create and implement eco-friendly goods derived from organic resources must include the green and renewable notion. The term “bio-based materials” refers to substances derived from agricultural commodities and food waste. In this way, bio-based products address environmental concerns and use renewable resources in novel ways.^27^ CL mostly comes from plants and has a flat ribbon-like shape due to its linear polysaccharide structure that is produced by β-(1,4)-linked d-glucose units. The van der Waals forces and hydrogen bonding network between and within the chains of cellulose make its disintegration difficult, which contributes to its relative stability. Supramolecular interactions sustain the coupling of individual CL chains, which gives CL fibrils their linear structure and axial rigidity. Among the many benefits of cellulose are its status as one of the safest materials in the world, excellent mechanical strength, biodegradability, and biocompatibility. Cellulose, on the other hand, is poorly soluble in both water and most organic solvents. Ionic liquids, lithium chloride, NaOH/urea, and NaOH/thiourea are among the solvent systems that may be used to modify the application of cellulose^27^ Due to its abundance, sustainability, and decreased immunogenicity, cellulose sulfate is an intriguing vehicle for growth factor administration in cartilage tissue engineering. Its potential future applications are even more expansive. The biological characteristics of these scaffolds may be fine-tuned and customized by the exact regulation of the sulfation pattern and degree made possible by the backbone sulfation of cellulose. A wider variety of biomaterials may be possible because of the many chemical alterations that are now accessible, which open the door to mechanical and pharmacological qualities that may be tuned. The decomposition and defibrillation of cellulose (Cel) fibers with H_2_SO_4_ produced cellulose sulfate (sCel) esters, which was one of the first instances of chemically sulfated polysaccharides.^5^

#### 4.1.3 Carboxymethyl cellulose sulfate (SCMC)

**Fig. 3:**
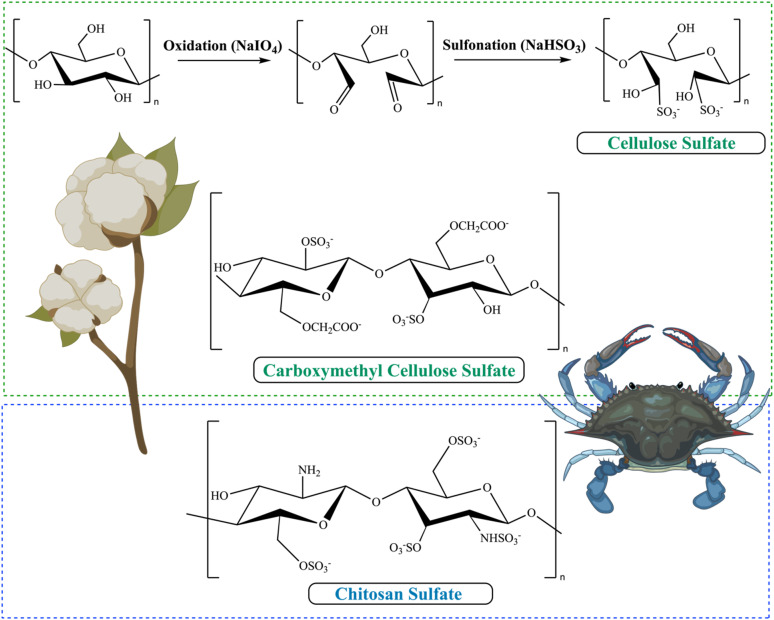
Synthesis of sulfonated cellulose, chemical structure of SCMC, CS.

Among the chemically enhanced celluloses manufactured in industrial settings, carboxymethyl cellulose (CMC) is among the most prevalent. CMC's reactive carboxyl and hydroxyl groups provide it excellent solubility and reactivity, unlike cellulose, which does not have these groups.^5^ CMC is a cellulose derivative that is constantly used in the biopolymer industry; it is water-soluble. One way it is made is by partially replacing the hydroxyl groups 2, 3, and 6 on the cellulose backbone with carboxymethyl groups. Being a cheap and plentiful natural biopolymer on Earth, cellulose with numerous hydroxyl groups might be an intriguing starting material. Additionally, CMC has biodegradability, solubility, and biocompatibility. A non-aqueous solvent medium consisting of monochloroacetic acid and soda is used to produce CMC in order to determine the degree of substitution *via* carboxymethylation. Potential applications for hydrogels based on CMC include adsorbents, wound healing, medication delivery, and enzyme immobilization. There are many potential applications for CMC/nanoparticle hydrogel, including antimicrobial action, medication delivery, wound healing, and tissue engineering. The performance of CMC hydrogel is enhanced when nanoparticles are added to it. To enhance CMC hydrogels, nanoparticles are used because of their remarkable chemical, mechanical, electrical, and optical capabilities.^27^ As a weak acid polyelectrolyte, CMC is common (p*K*_a_ ∼3.8). On top of that, it gets along well with the skin and other mucous membranes. Viscosity, building, and flocculation are the three most significant characteristics of CMC. A pure CMC sample has 23 of these characteristics: it is odorless, biodegradable, biocompatible, and white to off-white in color.^2^ Sulfated carboxymethyl cellulose (SCMC) has a significant number of hydroxyl hydrophilic groups scattered throughout its backbone. Adding additional hydrophilic functional groups, such as sulfonate, to the backbone of natural CMC may increase its water absorption capabilities. Sulfonating reactions may be used to modify CMC, resulting in sulfonated SCMC [Fig. 3].^76^ The sulfate groups that are part of SCMC are responsible for its characteristics, which include a higher charge density and hydrophilicity, as well as an improvement in the electrostatic repulsion between the membranes and negative ions.^77^

#### 4.1.4 Chitosan sulfate (ChS)

Chitosan (ChS) is a remarkable amino-polysaccharide found in nature; it is also the only polysaccharide known to have a positively charged structure.^67,78^ ChS can be produced by withdrawing the acetyl functional groups from chitin through an alkaline deacetylation process, which is carried out under alkaline circumstances using a strong base (*e.g.*, sodium hydroxide). The charge density is determined by the degree of acetylation and the pH of the solution applied.^69,78–80^ Chitin is the predominant amino-polysaccharide found in nature and is the second most abundant organic substance in the world, following cellulose.^69,81,82^ Chitin is found in a number of marine organisms, among them the exoskeletons of crustaceans (such as shrimps, crabs, and lobsters), marine invertebrates (barnacles), mollusks (such as cuttlefish and octopus), the cell walls of fungi, the cuticles of insects and arachnids, as well as green and brown algae.^69,79^ Chitin, analogous to cellulose, having hydroxyl groups on the C-2 positions of glucose, which is a building block of cellulose. In chitin, these hydroxyl groups are substituted by acetamide groups.^3,67,69,81^ Chitin possesses tough and crystalline structure, making it insoluble in water because of the existence of hydrogen bonds among its molecular chains. Deacetylating chitin to ChS results in an increase in the number of amino groups, which in turn leads to the formation of a positively charged polymer.^83^ Chitosan is an alkaline heteropolysaccharide that occurs naturally and is made of β-(1–4)-glycosidic connections between d-glucosamine and *N*-acetyl-d-glucosamine residues. It is linear and random in structure and has cationic properties. The compound consists of two monosaccharide units, in particular β-1,4-linked 2-amino-2-deoxy-β-d-glucopyranose and 2-acetylamino-2-deoxy-β-d-glucopyranose residues.^69,83–85^ Chitosan, which have been included in the list of generally recognized as safe (GRAS) materials by the U.S. Food and Drug Administration in 2001, is extensively utilized in the field of biology due to its biocompatibility, non-toxicity, and biodegradability properties.^82^ The presence of amino groups in chitosan's structure (NH_2_) is the primary factor behind its unique potential in several applications. For example, including absorption enhancement, bio-adhesion, transfection efficiency, and demonstrates significant biological activities such as antimicrobial, antitumor, anti-hypercholesterolemia, and anti-inflammatory characteristics.^83,86^ When chitosan is dissolved in an acidic environment, the amino groups in the chain structure get a proton and the polymer becomes positively charged, enabling it to interact with many types of molecules. Chitosan has been thorough investigation because of its exceptional capacity to form films, its antibacterial characteristics, its physical and mechanical properties, its biocompatibility with organisms, and its tendency to biodegradability. Furthermore, ChS has antibacterial properties because the positive charge in ChS interacts with the negatively charged cell membranes of bacteria and viruses. This interaction leads to changes in the structure and permeability of the bacterial cell envelope. Regarded as a semi-crystalline polysaccharide, it is unable to dissolve in water-based solutions with a pH over 7. However, when exposed to weakly acidic solutions with a pH of 6, the amine groups of the molecule are protonated, resulting in increased solubility.^80^ Nevertheless, the medical application of ChS is restricted by its crystalline form, which results in a high molecular weight and low solubility in aqueous media. Its cationic nature sets it apart from other biopolymers, making it an ideal choice for creating hydrogels used in drug delivery applications.^83^ Chitosan's exceptional capacity biodegradability, lack of toxicity, biocompatibility, antibacterial, ability to form films, and ability to create emulsions make it highly useful in a wide range of medical applications, particularly in healing wounds and delivering bioactive substances. Chitosan has the ability to create hydrogels by means of covalent bonds, hydrogen bonds, electrostatic contacts, and hydrophobic interactions, either individually or in combination with other substances.^85^ Chitosan can be easily modified chemically without affecting its degree of polymerization. These functional groups include a primary amine group (NH) at the C2 position, as well as primary and secondary hydroxyl groups (OH) at the C3 and C6 positions in its backbone. Glycosidic linkages and the acetamide group are included.^78^ Typically, the NH_2_–amino group exhibits more reactivity compared to the C6–OH primary hydroxyl group, primarily due to its free rotation. Similarly, the primary hydroxyl group has higher reactivity than the C3–OH secondary hydroxyl group. The chemical modification involves substitution of amino, hydroxyl, or both amino and hydroxyl groups to create derivatives of chitosan that are either N–, O–, or N, O–modified.^84^ This modification also enhances the overall properties of the polysaccharides, making it suitable for creating sustained drug release systems.^78,86^ The production of chitosan derivatives aims to enhance the present features of chitosan, such as biocompatibility and biodegradability, or to add novel functions or properties.^78^ The sulfation of chitosan involves the utilization of several sulfating reagents such as concentrated H_2_SO_4_, SO_3_, SO_3_/pyridine, SO_3_/trimethyl amine, SO_3_/SO_2_, and chlorosulfonic acid-sulfuric acid [Fig. 3]. The *O*-sulfated derivative and *N*-sulfated chitosan are both being studied in biomedical applications. It has been found that the degree of sulfate substitution affects the anticoagulant action of chitosan. This is because chitosan with a high degree of sulfate substitution has a similar anticoagulant activity to heparin, perhaps due to their structural similarities.^87^ The anticoagulant activity of sulfated chitosan arises from the interaction between –SO_4_^2−^ groups and positively charged peptide sequences. In addition, research has demonstrated that the presence of *N*-acetyl groups enhances the effectiveness of anticoagulant action as well. Chitosan sulfates have been found to exhibit several biological functions, such as preventing hardening of the arteries, protecting against oxidative damage, antiviral, inhibiting the HIV virus, antibacterial, and inhibiting the activity of enzymes.^86^ Moreover, sulfated chitosan have a strong ability to remove free radical ions, such as hydroxyl and superoxide ions.^87^

#### 4.1.5 Alginate sulfate (SALG)

Alginate (ALG), a derivative of naturally occurring alginic acid, is a linear anionic-polysaccharide derived from almost all brown seaweed cell walls, such as *kelp* species (*Macrocystis pyrifera* and *Ascophyllum nodosum*), various types of *Laminaria* species (*Laminaria hyperborea*, *Laminaria digitata*, and *Laminaria japonica*), *Sargassum* species (*Sargassum turbinaroides*), *Durvillaea antarctica*, *Lessonia nigrescens*, and *Ecklonia maxima*, and less frequently from capsules of bacteria (Gram-negative bacteria) such as *Pseudomonas* and *Azotobacter*.^33,37,67,69,82^ ALG is composed of two repeating monosaccharide blocklike patterns: 1,4-glycosidic connections between β-d-mannuronic acid (M) and α-l-guluronic acid (G) residues.^67,69,82,85^ The structural molecular chain of ALG is composed of the following three different fragments: homopolymers of G blocks (GG polymer) and M blocks (MM polymer) and heteropolymers of randomly coupled G and M blocks (GM polymer or MG polymer) [Fig. 2].^69,70^ The proportion of these two blocks and the molecular weight of polysaccharide are determined by the algae of origin; moreover, have critical effect on the physicochemical properties of alginate.^70,83^ Generally, the G unit provides rigidity to the ALG, while the M unit offers flexibility.^67,88^ Because of the anionic nature imparted to alginate by the carboxylate group on the α-l-guluronate residue, it forms an ionic hydrogel when it binds to cationic ions like calcium.^45^ The G-blocks possess exceptional stiffness and steric hindrance properties, which make them very suitable for many applications, such as cartilage regeneration in tissue engineering and cell immobilization sectors.^84^ Furthermore, alginate provides several notable benefits including non-toxicity, biocompatibility, biodegradability, bioactivity, environmental friendliness, cost-effectiveness, abundance in nature, and the ability to stabilize, and form gels.^2,6^ Alginate exhibits an outstanding characteristic in which G-blocks interact with different cations (*e.g.*, Ca^2+^, Mg^2+^, *etc.*), enabling the formation of 3D network hydrogels at suitable pH and temperature conditions. Hydrogels are used in many medicinal applications, such as drugs release, as well as providing a suitable environment for cell growth.^2,3,31,67^ This approach is the most used for creating alginate gels. Furthermore, there exist several techniques for the creation of alginate hydrogels, such as ion-interaction, covalent cross-linking, thermal gelation, and cell cross-linking.^69^ The alginate gel, which is created by the introduction of divalent cations, has several applications such as wound healing, the pharmacy industry, and the transplant of cells.^53,67^ The remaining two sections of ALG, classified as heterogeneous fragments (MG), have been transformed into the innovative marine medicine 911, which is currently undergoing Phase II clinical trials as a potential treatment for HIV. The homogeneous pieces (M blocks) of the alginate polysaccharide have been utilized to create the marine medicine polymannuronic acid propyl sulfate (PMS), which is effective against cardiovascular diseases.^88^ Nevertheless, the insufficiency of mechanical qualities, unmanageable degradation profiles, and absence of cell recognition signals have hindered its medical applications. The presence of carboxyl groups and hydroxyl groups along the alginate backbone allows for a range of modification methods to improve or customize aspects such as physicochemical, biological, mechanical, and other desired characteristics. The chemical modification methods include graft copolymerization, cross-linking with cationic polymers, and adding new groups to the alginate (such as sulfate groups). These chemical modifications led to well-suited alginate derivatives for use in tissue engineering and drug delivery systems after enhancing its biodegradability, gelling capacity, and biocompatibility properties. In addition, self-assembly methods, such as layer-by-layer assembly, can be employed to create functional multilayer alginate capsules with core–shell structures for a range of biological purposes. However, it is important to exercise caution since an excessive number of groups may compromise the gelation capacity of alginate, which is one of its most notable characteristics.^84^ The efficacy of alginate is impeded by its increased molecular weight, extending chain configuration, limited water solubility, and weakened bioavailability. Hence, it is essential to degrade alginate to obtain polysaccharides or oligosaccharides with low molecular weight, as this improves their bioavailability.^89^ Alginate is capable of undergoing modifications to produce a range of different derivatives. The most encountered derivatives are:

Polymannuronic acid (PMS) is a type of alginate with small and homogeneous units (M blocks). It is obtained from alginate polysaccharides using processes such as enzymatic or acidic degradation, PH fractionation method.^88^ ion exchange column chromatography, or gel column chromatography. PMS demonstrates a range of biological actions, such as anticancer, antioxidant, immunoregulatory, obesity-inhibiting, blood pressure-reducing, blood lipid-lowering, and blood glucose-lowering effects.^89^

Polymannuroguluronate sulfate (PMGS) is a type of alginate with a low molecular weight. The substance is distinguished by a high concentration of 1,4-linked *b*-d-mannuronate with an average of 1.02 sulfate and 1.0 carboxyl groups per sugar residue. The first medication candidate derived from marine algae to combat acquired immune deficiency syndrome (AIDS) has commenced Phase II clinical trials in China. SPMG has commenced the Phase II clinical trial in China. Therefore, it is the first marine sulfated polysaccharide that has the potential to be developed as an anti-AIDS treatment.^90^ Wang *et al.* point out that alginate-derived polysaccharide polymannuroguluronate sulfate (PMGS) exhibited anti-HPV properties both *in vitro* and *in vivo* with barely toxicity. PMGS may inhibit HPV binding and entrance by direct contact with the viral capsid L1 protein. PMGS may infiltrate HeLa cells and suppress the production of the viral oncogene proteins E6 and E7. PMGS might markedly inhibit high-risk HPV45 infection in murine dermis.^91^

Polyguluronate sulfate (PGS) is another type of alginate with a low molecular weight which is a substance derived from sulfating process of α-1,4-poly-l-guluronic (PG) acid. The sulfation reaction of polysaccharides can enhance their blood compatibility and anticoagulant activity. As well as the antiviral effects against HBV in HepG2.2.15 cells; hepatocyte damage, particularly liver injury caused by the immune system, plays a crucial role in the development of liver disorders generated by hepatitis viruses.^92^

#### 4.1.6 Laminarin sulfate

Laminarin, a water-soluble polysaccharide found in brown algae, is gaining recognition for its biological activities. It is a biodegradable and non-toxic compound stored in the cell walls of brown algae, particularly in species like *Saccharina* and *Laminaria*.^6,93,94^ Natural laminarin usually has a molecular weight of approximately 5 kDa and consists of 20–25 units of d-glucopyranose connected by linear β-(1 → 3) glycosidic linkages [Fig. 2]. The backbone of laminarin may also composed of branching β-(1 → 6) glycosidic bonds, depending on factors such as habitat, season, and location of extraction. Laminarin's biological activity was expanded further *via* the addition of sulfate groups. The chemically sulfated laminarin was generated with a 1.5 substitution degree using the chlorosulfonic acid-pyridine technique. Sulfate content is usually found at O-6, O-2, and/or O-4 of the glucosyl unit backbone, and it can range from 34% to 50%. The quantity and position of sulfates within the polysaccharide chain are believed to be key factors for enhancing biological activity, which includes tissue engineering, anti-tumor, anticoagulant, antioxidant, and anti-inflammatory activities. It is also used in the delivery of drugs and genes, and cancer treatments.^63,93,94^ Xu Q. *et al.*, examine the recuperative effects of four Laminaria polysaccharides (SLPs) with differing sulfate (–OSO_3_^−^) concentrations on injured HK-2 cells. The crystal adhesion upon damage to renal tubular epithelial cells (HK-2) is crucial in the formation of kidney stones. Additionally, the variations in the adhesion of these compromised cells to nanometer calcium oxalate monohydrate (COM) and calcium oxalate dihydrate (COD) pre- and post-recovery. The capacity of SLPs to restore injured HK-2 cells and prevent crystal adherence is proportional to their sulfate concentration.^95^ Similar results were reported where laminarin sulfate reduces the chances of kidney stone development and protects HK-2 cells from nano-COM crystal-induced apoptosis by lowering levels of oxidative and endogenous stress and the variables that contribute to it.^96,97^

#### 4.1.7 Hyaluronic acid sulfate (HAS)

In 1934, a novel polysaccharide possessing a high molecular weight was isolated from the vitreous fluid of bovine animals by Salave *et al.* It was discovered that this polysaccharide consists of an amino sugar and uronic acid. The scientist designated this novel polysaccharide as hyaluronic acid, which is derived from the terms “hyaloid” (referring to the vitreous) and “uronic acid”. Upon nearly two decades of investigation, the composition of the repeating disaccharide unit comprising this polysaccharide was determined to be infallible.^53^ Hyaluronic acid (HA) is the predominant glycosaminoglycan among the majority of mammalian species; microorganisms are also capable of synthesizing it.^12^ HA can be extracted from a variety of marine organism components, such as skin, cartilage, cranium, eyes, and fins. In general, the vitreous humor serves as the principal hyaluronic acid producer.^69,70^ Hyaluronic acid is a linear glycosaminoglycan (GAG) that is present in nature. The compound consists of *N*-acetyl-d-glucosamine and d-glucuronic acid repeat units that are alternately joined by β-1,4 and β-1,3 glycosidic linkages [Fig. 2].^3,53^ HA can be generated naturally in the body through degradation, and it occurs through two primary methods. The first method utilizes hyaluronidases, which are enzymatic catalysts responsible for hydrolyzing the β-(1,4) linkages connecting *N*-acetyl-d-glucosamine and d-glucuronic acid residues. A free radical mechanism occurs in the presence of specific reducing compounds, including cuprous ions and ascorbic acid, in the second method. Hyaluronic acid, in the form of hyaluronate, is abundantly present in humans, particularly in the skin, umbilical cord, and vitreous humor.^53^ Hyaluronic acid is the only non-sulfated glycosaminoglycan found in nature. In its sulfated form, it demonstrates superior properties relative to unsulfated HA,^98^ such as anticoagulant,^99^ anticancer,^100,101^ anti-inflammatory, and several other bioactivities, reachable by a chemical modification of hydroxyl groups. These modifications enhance its negative charge and can affect cellular signaling, interactions, and behavior. The sulfation of HA in stem cell culture can modify its interaction with cell surface receptors, influencing cellular activity. Sulfated hyaluronic acid can function as a crucial element in a more defined, feeder-free environment, supporting the undifferentiated state and pluripotency of human-induced pluripotent stem (hiPS) cells. It aids in the preservation of stem cells and could potentially improve their standardization. Sulfated HA significantly reduced degradation by hyaluronidase in comparison to non-sulfated HA. Hyaluronic acid cannot bind proteins with high affinity due to the absence of negatively charged sulfate groups. Changing the hydrophobic part makes it easier to change the hydrophilic and degradation rates. For example, sulfated HA breaks down much more slowly by hyaluronidase (HYA). Moreover, sulfation facilitates the integration of protein medicines into the hydrogel matrix through the sequestration effect. The sulfated HA markedly enhances protein sequestration, thereby substantially prolonging the availability of protein-based medicines in the hydrogels. Sulfated HA hydrogels enhance the retention of transforming growth factor (TGF) within the hydrogels, consequently promoting chondrogenesis and inhibiting the hypertrophy of encapsulated human mesenchymal stem cells (hMSCs). Furthermore, sulfated HA nanoparticles proficiently target both P-selectin+ and CD44+ cells.

### 4.2. Graft copolymerization of SPs

**Fig. 4:**
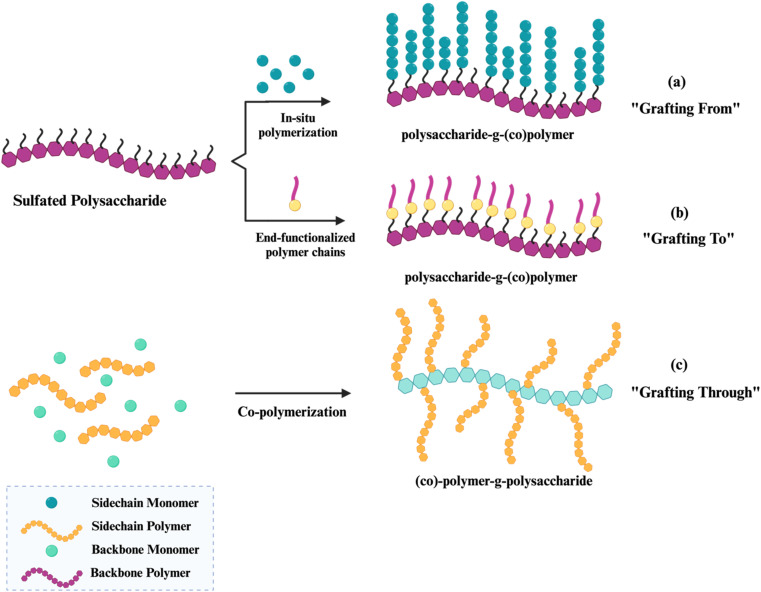
Schematic representation of modification polysaccharides of the (a) grafting from, (b) grafting to, (c) grafting through approach for graft copolymerization.

Copolymer by polymer–polymer interactions or by grafting copolymerization is a highly effective approach for modifying the surface properties of polysaccharides.^12,13^ The principal objectives of surface modification are to enhance the mechanical and physicochemical features of a polymer's surface. For instance, its rheological properties, hydrophilic ability, molecular chain, biocompatibility, thermal stability and strength, in comparison to each of these properties in these unmodified polysaccharides individually.^13,14^ Graft polymerization involves of two parts the chemical attachment of “side chains” or “graft chains” to the core backbone polymer “main chain” *via* covalent bonds. These side chains have different constitution or configurational properties compared to the main chain.^13,14,102^ The presence of active sites, in the form of functional groups or free radicals on the backbone is the fundamental principle for the synthesis of graft copolymers.^13,14^ The properties of this type of copolymer are heavily influenced by the molecular characteristics of the grafted side chains, including their molecular structure, chain length, and degree of grafting. Both the main chain and the side chain polymers can be synthesized in either a homogenous or a varied environment, depending on the solubility of the monomer and the characteristics of the solvent employed for the reaction.^13^ Grafting procedures can be primarily categorized based on the grafting medium and the type of initiating mechanisms, which can either be homogeneous or in a heterogeneous medium.^103^ There are three fundamental mechanisms for the synthesis of graft copolymers:

• The “grafting from” approach (surface-initiated (SI) polymerization) is mainly employed to achieve targeted quantitative grafting densities [Fig. 4A]. The backbone of the polymer is chemically modified to incorporate active sites that serve as initiation points for the polymerization process, allowing for the attachment of the “graft chains”.^104^ The quantity of grafted chains can be regulated by the quantity of active sites produced along the backbone, assuming that each active site contributes to the creation of one branch.^102^ The utilization of this technique yields precise regulation over the copolymer structure, ensuring a high level of control and a low Mw/Mn. Consequently, it leads to the formation of copolymers with a well-defined structure and a desirable grafting density.^104^ However, the lengths of the generated grafts may differ primarily due to kinetic and steric hindrance effects.^102^ In this method, grafting is carried out using either a single monomer or a combination of two monomers.^103^ The grafting-from approach *in situ* polymerization, this process initially starts directly from the main chain, however the possibility of its free homopolymerization cannot be ruled out. This process is often carried out in a single step; however, it does not allow for any control over the macromolecular structure.

• The “grafting to” approach involves attaching pre-polymerized chains that having functional groups to a polymer backbone that has reactive end-groups [Fig. 4B].^14,102,103^ The graft copolymers take place through the coupling reaction between the functional backbone and the end-reactive branches.^102^ Prior to attachment to the polymer backbone, the polymer chains undergo synthesis. The substrate must have functional groups that can react with the terminal functional group of the produced polymers. This technique enables the synthesis of a well-defined backbone and side chain structure in advance. Despite the occurrence of low grafting densities caused by side reactions and steric hindrance effects, new advancements in “click chemistry” have addressed some of these concerns.^104^

• The “grafting through” approach involves the simultaneous synthesis of a polymer backbone using a macro monomer, an oligomeric, or a macromer, together with the polymerization of pre-prepared side chain monomers [Fig. 4C].^103,104^ This method takes place when there is a need for branching. While the side chains can be precisely characterized and have the appropriate molar masses and molar mass distributions, the synthesis of the backbone may trigger undesirable side reactions, resulting in polymers that are not well-defined.^104^

Grafting is also classified in the sense of the monomers attached as: (i) grafting (single monomer): this occurs in a singular, uninterrupted process. (ii) Grafting (combination of two (or more) monomers): this process takes place when two monomers are utilized together simultaneously or sequentially. Grafted co-polymerization is an attractive method for introducing various functional groups into the backbone of the polysaccharides.^14^ Graft copolymerization can be introduced in different methods include chemical approaches, radiation-induced grafting, enzymatic grafting, as well as plasma-initiated grafting. The chemical approach of graft copolymerization utilizes certain chemicals as initiators to create active groups on the polymer backbone such as utilization of different redox initiator systems, such as Lewis's acids, strong bases, and metal carbonyls.^13^ Free radical polymerization is the most widely employed method for grafting copolymer (*e.g.*, polyvinyl and polycrylic polymers). Generally, the procedure involves the formation of radicals by using initiators on the polysaccharide's backbone, followed by the polymerization of vinyl monomers onto the backbone. Nevertheless, free radical polymerization suffers from a lack of control over the polymer structure. Controlled radical polymerization (CRP) technologies, such as atom transfer radical polymerization (ATRP) and reversible addition–fragmentation chain transfer polymerization (RAFT), have gained interest due to their capacity to precisely control the displacement of polymer chains.^12^

### 4.3. Hybrid materials-based SPs biocomposites

#### 4.3.1 SPs bio-nanocomposites

##### 4.3.1.1 Nanocomposites

Bio-nanocomposites consist of a combination of two or more materials, where one is an organic matrix (such as biopolymers) and the other is nanomaterials (nanofiller) that combine on a nanometer scale (1 nm = 10^−9^ m) in at least one dimension. Typically, the nanocomposite is predominantly composed of the polymer matrix, contributing to its weight and volume.^24^ The nanofillers added to the polymer matrices are present in quantities less than 10 wt%, in contrast to traditional micro-composites which comprise 50 wt% of micro fillers. The nanostructure of a material plays a crucial role in the development of new characteristics and in the precise control of the structure at the nanoscale. Numerous artificial polymers and biopolymers possess environmentally friendly characteristics and are devoid of toxicity. Nevertheless, Nanoparticles offer a pathway to enhance performance by introducing them to a higher-level performance and/or unique features. Nanoparticles (NPs) possess exceptional mechanical, electrical, optical, and physicochemical characteristics (such as a high surface-to-volume ratio) and play a crucial role in creating innovative nanocomposites.^5,31,105^ Nanocomposite materials is potentially divided into three distinct categories based on the type of matrix materials used: (1) metal matrix, (2) ceramic matrix, and (3) polymeric matrix.

Metal matrix nanocomposites (MMNC) consist of two parts, the matrix that containing metallic particles, such as aluminum, cobalt, iron, and magnesium, *etc.* MMNC can be produced through various methods, including condensing metal vapor, thermally decomposing metal compounds, electrochemically depositing metallic nanoparticles in a polymer, and partially encapsulating nanoparticles with polytetrafluoroethylene (PTFE).

Ceramic matrix nanocomposites (CMNC) consist of ceramic fibers securely embedded in a ceramic matrix, such as Al_2_O_3_/ZrO_2_ and ceramic/CNTs.

Polymer matrix nanocomposites (PMNC) is the most common method that have been used to prepare nano composites. The matrix consists of a polymer or copolymer with nanofillers dispersed throughout the polymer matrix, such as polymer/layered silicates, PS/Fe_2_O_3_, and PS/TiO_2_.

##### 4.3.1.2 Types of nanofillers

**Fig. 5:**
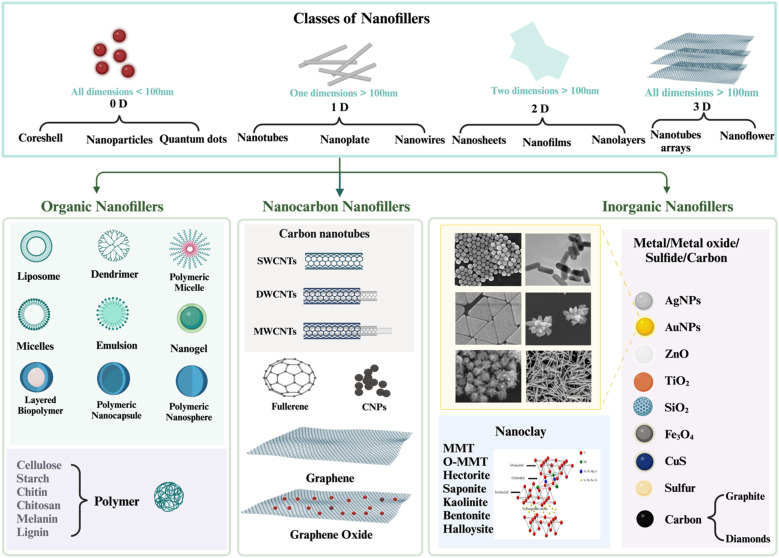
The Schematic and SEM images for different types and shapes of nanofillers. Created with https://www.BioRender.com/.

Based on dimension structures, nanofillers can be categorized into four types: dimensionless (0D) nanofillers in which all the three dimensions of the nanomaterials have no dimensions exceeding 100 nm (*e.g.* nanoparticles, quantum dots, fullerenes). One-dimensional (1D) nanofillers are typically have one dimension exceeding 100 nm (*e.g.* nanotubes, nanowires, nanorode, nanofibers, nanohorns). Two-dimensional (2D) nanofillers in which the nano materials have two dimensions exceeding 100 nm (*e.g.* nanosheets, nanofilms, nanolayers). Three-dimensional (3D) nanofillers, all the three dimensions of the nanomaterials are exceeding 100 nm (*e.g.*, arrays of nanotubes or nanowires, nanoflowers, graphite).^106^ Nanofillers can be classified as either organic or inorganic materials based on their source [Fig. 5].

Organic nanofillers: these examples encompass cellulose nanoparticles, cellulose nanowhiskers, cellulose nanofibrils, chitin nanofibrils, starch nanocrystals, and so on.^105^ Organic fillers are derived from living organisms and typically consist of carbon–hydrogen, carbon–carbon, and covalent bonds involving carbon, hydrogen, and nitrogen. There has been an increasing fascination with the utilization of ‘green’ nanomaterials, which consist of biopolymers. The motivation behind this interest arises from the necessity to mitigate nanotoxicity and tackle environmental issues.

Inorganic nanofillers: are obtained from non-living sources that experience lack in carbon–hydrogen bonds. Inorganic fillers encompass mineral or metallic fillings. These substances consist of nanoparticles made of metals or metal oxides such as silver, copper, zinc, and titanium, as well as clay minerals including clay, namely montmorillonite (MMT), nanoclay, silver nanoparticles (Ag NPs), and calcium carbonate (CaCO_3_) are often used inorganic materials to support polysaccharide-based composites. The clay layers are composed of two tetrahedral silicon atoms that are coordinated, together with an octahedral sheet made of either aluminum or magnesium hydroxide. The clay layer has a thickness of around 1 nanometer, and its size can range from a few nanometers to several micrometers or even larger. The specific dimensions rely on factors such as the method of preparation, the source of the clay, and the types of layered silicate. The selection of the most suitable nanoparticle is contingent upon the desired thermal, mechanical, and electrical characteristics.^5,31,105^

##### 4.3.1.3 Synthesis methods of hybrid SPs bio-nanocomposites

**Fig. 6:**
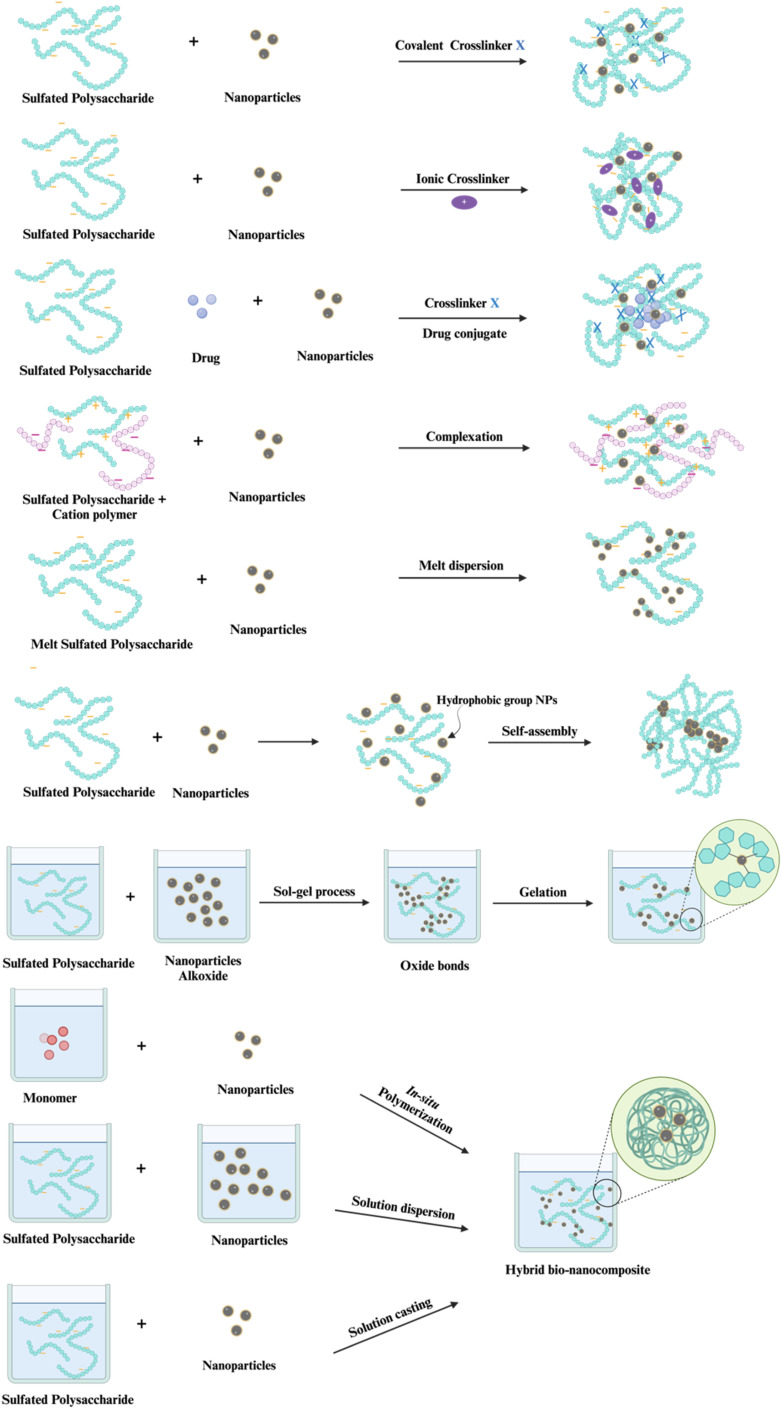
Different method of hybrid bio-nanocomposites preparation. Created with https://www.BioRender.com/.

Hybrid nanocomposites can be generated through many modifications, such as surface modification, altering the morphologies of materials, multi-functionalization, assembling, controlling the size, and adjusting the composition of components. Several effective technologies and procedures have been developed to simplify the production of nanocomposite materials [Fig. 6], including physical-chemical vapor deposition and plasma and thermal spraying.

(1) Solution casting method, sometimes referred to as solution mixing or blending method. In this technique, a homogenous solution is made by dissolve the polymer in an organic solvent (*e.g.* chloroform, dimethylformamide, acetonitrile) or water. Subsequently, nano fillers are introduced into the solution. The choice of solvent should be capable of fully dissolving both the polymer and nanofillers. Additionally, it should be able to evaporate under regulated conditions after the polymer nanocomposite has formed on the substrate surface. A thin film can be created by applying the film forming solution onto a glass plate. Polymer blend nanocomposites can have either a uniform composition throughout (homogeneous) or a non-uniform composition (heterogeneous). This method is more efficient and extensively adopted than other methods. However, the process is hindered by the challenge of identifying an appropriate solvent and subsequently eliminating it in the last stage. The solvents employed may also possess hazardous properties.^105^

(2) *In situ* polymerization method is a common technique for producing polymer nanocomposites. Nanocomposites have been synthesized using chemical reactions in a liquid medium, while physical approaches have been employed to functionalize nanoparticles as a core/shell structure. This procedure entails the physical blending of nanofiller with the chosen monomer. Subsequently, individual units of a substance insert themselves between the layers and trigger the separation of these layers. The nanofiller is evenly distributed within a liquid monomer or a solution containing monomers, allowing for the polymer to take place between the layers of the nanofiller by which avoid aggregation. This method eliminates the need for solvents.^105^ The sol–gel method (a type of *in situ* method) is highly efficient in adjusting both the size and morphology of nanoparticles. In this approach, a polymer nanocomposite is produced by dispersing a nanofiller in a liquid monomer matrix. Then, the normal polymerization process take place with or without a solvent. In this approach, monomers are used as the initial substance instead of polymers. The process primarily entails the creation of a sol; refers to a liquid phase containing solid particles that are dispersed in a colloidal suspension, followed by its gelation; the gel act as a binding agent by creating a network that holds the different phases together, and is mostly composed of hydrolysis and condensation processes. Precursors for this purpose can include metal alkoxides or other chemical and inorganic salts. This method is effective for insoluble polymers, thermally unstable polymers, and polymers that cannot be handled using melt compounding or solution methods.

(3) Solution intercalation method (solution dispersion): in this method, the nanofiller is pre-swelling in a solvent and separately dissolving the polymer using same solvent. Subsequently, the two solutions are combined, causing the polymer chains to infiltrate and replace the solvent in the nanofiller's interlayer. This technique is appropriate for incorporating polymers with low or no polarity into a layered structure and enables the creation of thin films with nanofiller layers that have polymer orientation. The utilization of this method has been extensively employed to generate intercalated nanocomposites using biopolymers that are soluble in water.^105^

(4) Melt processing method involves combining the polymer and nanofiller above the polymer's melting point under specific conditions to facilitate the incorporation or exfoliation of the nanofiller. For example, they are subjected to shear forces or maintained at the same temperature for a specific duration.^105^ The mixing can be accomplished by techniques such as injection molding or extrusion. The polymer's compatibility with existing processing equipment, its adaptability, and its environmentally friendly nature resulting from the lack of solvents. Nevertheless, the cost-effective and precise control over the distribution of fillers in the matrix is challenging due to the elevated viscosity of thermoplastic polymers.

(5) Polyelectrolyte complexes (PEC) are generated through the direct electrostatic interactions of polyelectrolytes having opposite charges in a solution. PECs offer an alternative approach for drug delivery that is biocompatible, as they utilize non-toxic covalent crosslinkers. These complexes have characteristics similar to ionic cross-linking, since they create non-permanent networks that are very responsive to variations in environmental conditions. However, in contrast to ionic cross-linking, where ions or ionic molecules undergo a reaction with the polyelectrolyte, in PEC the interaction occurs between the polyelectrolyte and polymer that have a wide variety of molecular weights. The development and stability of polyelectrolyte complexes (PECs) are primarily regulated by the extent of interaction between the polyelectrolytes. The latter refers to the charge density and distribution of each of the oppositely charged polyelectrolyte. The chemical environment is essential and includes factors such as the pH of the solution, the ionic strength, the temperature, and the duration and sequence of mixing. The secondary elements that influence the outcome are the molecular weight (Mw) of the polyelectrolytes and their level of flexibility. Ionic cross-linking enhances the strength of the established contact. Positively charged polysaccharides, specifically CS, could create polyelectrolyte complexes (PEC) with several negatively charged polymers, including alginate, dextran sulfate, chondroitin sulfate, hyaluronan, carboxymethyl cellulose, carrageenan, and heparin.^107^ Layer by layer assembly (LbL) of polyelectrolyte nanoparticles (NPs) is a recently developed method of creating nano-sized delivery systems based on polyelectrolytes. Basically, the LbL process begins by forming the first layer, by electrostatic interactions between positively charged solid supports and a negatively charged polyelectrolyte. After removing any excess polyelectrolyte, the solid support is then placed in a solution containing a different polyelectrolyte to form the second layer. This process is repeated until the desired thickness is achieved resulting in alternating terminal charges with each subsequent layer deposition and assembly a three-dimensional surface. This method has potential applications in creating nano-sized delivery systems. Since it allows for incorporating various treatments and biomaterials into LbL films through non-covalent interactions under physiological conditions, while preserving their biological properties. This enables the development of extensive biomedical applications in biosensing, tissue engineering and sophisticated nano-scale drug delivery system with a customized mechanism for drug release. In addition, LbL NPs can be utilized for the transportation of many active chemicals while having the capability to regulate the release of each enclosed ingredient. The potential to achieve sequential drug release from LbL film layers allows for precise control over the order and time of multiple drug release.^107^

(6) Polysaccharide–drug conjugate is formed by attaching the drug to the polysaccharide. This attachment changes how the medications are distributed and how long they circulate in the body. After conjugation, the enhanced drugs can be targeted and delivered directly to the tumor site, due to the enhanced permeability and retention (EPR) effect. Several phase I/II clinical trials have been conducted due to the advantages of this delivery technique. This innovative idea has been employed in the creation of polysaccharide–drug conjugates, specifically for transporting anticancer medicines that are not soluble. The following explanation will provide more specific information. The polysaccharide–drug conjugate comprises three components: the hydrophilic polymer, the pharmaceutical agent, and the biodegradable linker that connects them. Incorporating supplementary elements like as labeling agents and targeting moieties is also possible. Particularly emphasis should be placed on the selection of the spacer while designing this delivery method. For a delivery system to be successful, it is important to consider the following principles: firstly, the spacer should exhibit stability in the bloodstream in order to prolong circulation duration, but it should also be quickly broken down once it enters the cell. Furthermore, the spacer is promoting drug molecule without modifying its chemical composition. Moreover, it is essential for the polysaccharide to possess sufficient stability inside the circulation. The ultimate dimensions and configuration of the polysaccharide–drug conjugate are heavily influenced by the properties of its constituents. For instance, when a hydrophobic drug is attached, it leads to the formation of a spherical nanoparticle where the drug is physically enclosed within the particle.^107^

## 5. Other common methods

Crosslinking method: the polymeric chains are linked together by crosslinkers, resulting in the creation of a three-dimensional network, as well as it stabilizes the structures of the NPs. The characteristics of a crosslinked NPs dependable on the cross-linking density, measured by the molar ratio between the crosslinker and the polymer repeating units.^2^ Based on the nature of the cross-linking agents, there are two types of crosslinked nanoparticles.^107^ The covalent crosslinkers are compounds that possess a minimum of two reactive functional groups on both components, allowing them to form connections between polymeric chains by creating covalent bridges. Dialdehydes are the most often employed covalent crosslinkers for polysaccharides.^107^ The second type is the ionic cross-linking is a straightforward alternative to covalent cross-linking for charged polysaccharides. This technique allows for the synthesis of nanoparticles through the creation of reversible ionic cross-links. Because no harsh or poisonous crosslinkers are employed in the process, these nanoparticles are commonly regarded as biocompatible. Positively charged polysaccharides have the ability to create ionic crosslinked nanoparticles when combined with negatively charged ions. The cross-linking response is influenced by various parameters, with the size of the crosslinker being particularly important. Contrary to covalently crosslinked nanoparticles, ionic crosslinked particles typically exhibit pH sensitivity, which is advantageous for drug delivery applications. Nevertheless, this sensitivity to pH can also lead to instability of the network that is crosslinked by ions.^107^

Self-assembly is the process of attaching the hydrophobic groups to a hydrophilic polysaccharide, resulting in the formation of an amphiphilic copolymer. These copolymers tend to spontaneously form nanoparticles (NPs) in water-based solutions. The NPs have hydrophobic shell acts as a stabilizing boundary between the hydrophilic core of the polysaccharides and its surrounding watery environment. Furthermore, due to the hydrophobic nature of the core, these particles have been employed for the transportation of hydrophobic drugs. For a specific purpose, several characteristics of a substance can be modified, including its dimensions, electric charge on the surface, ability to hold other substances, durability, and dispersion throughout living organisms. For instance, the size of the nanoparticles can be regulated by manipulating the dimensions of the hydrophobic component and the polymer. Scaling relations have been formulated for this specific purpose. Furthermore, the modification of the degree of substitution, the length, or the type of the hydrophobic component can be used to manipulate the surface charge of particles, hence influencing their serum stability and cellular uptake.^107^

Overall, the process of generating polysaccharide nanocomposites requires meticulous selection of techniques and parameters in order to attain the necessary dimensions, structure, and characteristics. The selection of the appropriate approach is dependent upon the required polymer matrix for specific applications *e.g.*, nanomedicine.^2^

### 5.1. Properties of sulfated polysaccharides nanocomposites

Nanocomposites possess unique features that are unattainable in bulk materials at the macro scale. The qualities of nanoscale materials are contingent upon their size, shape, and structure. As the size of the nanoparticles decreases, the surface area per unit volume increases. Due to their significant surface area to volume ratio, even a small number of nanoparticles in a biopolymer matrix can greatly impact the physical and material properties of biopolymers. Different types of nanoparticles can confer distinct characteristics to the nanocomposites. They influence various characteristics, including electrical and thermal conductivity, thermal stability, and density.^105^ SPs nanocomposites have revealed the advantages of nanomaterial additions that are absent in standard fillers and polymers. In addition, it takes advantage of the inherent traits of polymers, such as flexibility, ductility, biodegradability, biocompatibility, and processability.^6^ on the other hand, it capitalizes on the intrinsic characteristics of nanomaterials. For instance, several advantages are associated with the development of nanomaterials for use in cancer therapy, including reduced risk of toxicity and adverse effects of traditional drugs, targeted binding to specific cells, enhancement of solubility, stability, tumor accumulation, and half-life of conventional pharmaceuticals, release of the encapsulated drug *via* a stimuli-responsive mechanism, enhancement of the interaction surface for encapsulated drugs or those conjugated to biomacromolecules, mitigation of drug resistance through the targeted delivery of multiple active agents, circumvention of biological barriers, augmentation of diagnostic and imaging sensitivity, real-time evaluation of drug efficacy by integrating imaging agents with active anticancer compounds, facilitation of novel vaccine development, advancement of cancer diagnostics and imaging through the utilization of smaller devices.^108^ This integration allows for the desired properties to be achieved through the synergistic combination of these distinct characteristics.^6^ The properties of polymer nanocomposites have been significantly enhanced and elevated due to the advanced technology.^105^

### 5.2. Challenges in hybrid SPs bio-nanocomposites

An inherent obstacle to the production of bio-nanocomposites is the tendency of nanoparticles to aggregate, making it challenging to disperse them uniformly as fillers inside the polymer matrix. The dispersion becomes more challenging as the filler's surface energy differences with the matrix increase and the specific surface area of the filler. Aggregation is the process in which smaller particles at the nano scale come together to generate progressively larger ones. Another result is the low filling degree, which is influenced by various factors including particle friction, hydrogen bonding, and van der Waals forces. Nanoparticles frequently exhibit a higher attraction towards each other than towards the surrounding matrix due to van der Waals interactions, resulting in the phenomenon of agglomeration. Another disadvantage is the challenge in accurately forecasting the properties of nanocomposites. Surface modification of inorganic nanoparticles is a viable solution to address the aggregation problem. Commonly, plasticizers like glycerol, tryethylcitrate, or vegetable oils are incorporated into biopolymers nanocomposites that have a melting temperature around their decomposition temperature. These plasticizers additionally contribute to improved dispersion of the nanofiller within the biopolymer matrix, leading to enhanced mechanical characteristics.^24^ Prior to adding polysaccharide composites into the polysaccharide matrix, it is crucial to address the structural variables such as form, size, and filler loading in order to broaden their uses. Achieving a uniform distribution of nanofillers across a polymeric matrix is the initial and crucial stage in obtaining the improved characteristics of nanocomposites. The presence of hydroxyl groups on the surface of these nanofillers leads to a strong inclination for self-agglomeration. The interparticle interactions of nanofillers might lead to aggregation during the creation of the nanocomposite, resulting in the loss of the nanoscale dimension. This limits their potential utility. Various techniques are utilized in the process of creating nanocomposite films to reduce the clustering of nanofillers.^105^

#### 5.2.1 SPs hybrid hydrogels biocomposites

Hydrogels are composed of hydrated polymer networks or hydrophilic groups such as –OH, –COOH, –NH_2_, –CONH_2_, and –SO_3_H are assembled in an aqueous environment. Chemical synthesis of hydrogels begins with either a one-step or a multi-step method. The polymerization method and parallel crosslinking, which join several monomers, are always a part of the one-step process. Among the several steps involved is the creation of the polymer molecule's reactive group. Hydrogel structures, including hydrological response to stimuli, mechanical strength, biodegradability, and crosslinking density, may be controlled at the scale during design and synthesis. Because of their very porous nature, most hydrogels are made from synthetic polymers, which allow them to retain a high-water capacity. As a result, it works well with low-dose delivery systems.^27^ Xu *et al.* investigated the capacity of a novel *Arthrospira*-derived sulfated polysaccharide to enhance the behavior of neural stem cells (NSCs) inside a three-dimensional hydrogel. The incorporation of sulfated polysaccharide into the hydrogel significantly improved the proliferation and differentiation of implanted neural stem cells, while also extending their stemness. Consequently, it represents a viable choice for cell culture, surface modification, and drug delivery applications within the biomedical domain.^109^

## 6. Biological applications of SPs

Sulfated polysaccharides (SPs) possess distinctive characteristics that have been extensively studied for their potential in the development of biomaterials to meet diverse biomedical needs. Furthermore, researchers have investigated the incorporation of hybrid sulfated polysaccharides into nanocomposites and/or hydrogels for many medical applications, including the formulation of drug delivery systems, the fabrication of tissue engineering scaffolds, and regenerative medicine. These materials possess the capability for drug delivery, imaging, and other therapeutic applications.

### 6.1. Biological activities applications of SPs

Understanding the biochemical and molecular mechanisms behind the therapeutic activities of SPs is crucial for the development of successful pharmaceuticals.^110^

#### 6.1.1 Anticoagulant and antithrombotic activities

**Fig. 7:**
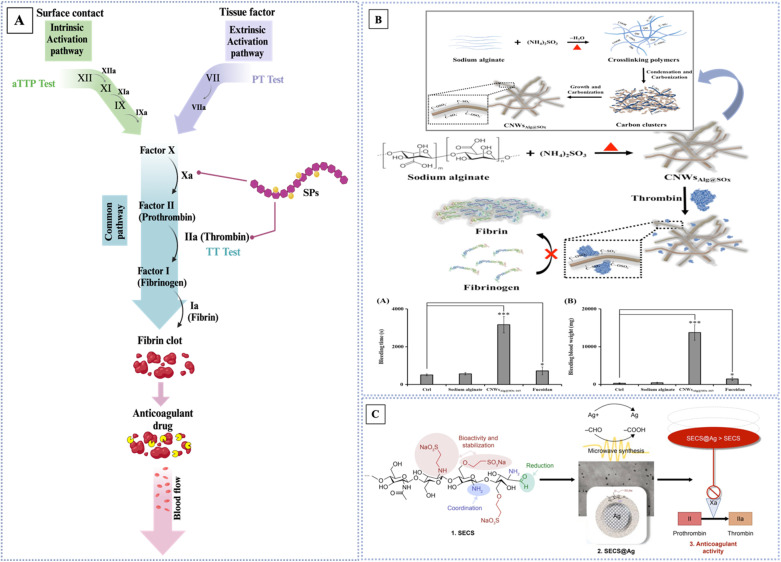
(A) Main coagulation pathways of blood coagulation systems and proposed mechanisms of SPs as anticoagulating agents. Intrinsic pathway measured by aPTT, extrinsic measured by PT. Created in BioRender. Alfinaikh R. (2024) https://app.BioRender.com/illustrations/6658d335202d9e5d919bf1d4. (B) Schematic representation of the one-pot synthesis of sulfonated/sulfated bio-carbon nanowires from sodium alginate and ammonium sulfite mixture for enzymatic inhibition of the thrombin-mediated catalytic reaction of fibrinogen to fibrin. The proposed mechanism of the formation of CNWs_Alg@SO_*x*_-165_. The effect of sodium alginate, CNWs_Alg@SO_*x*_-165_ or fucoidan on rat-tail bleeding times on the left and released blood weight on the right with the doses of the inhibitors (2.0 10^1^ mg) administered *via* intravenous (IV) injection. Error bars represent the standard deviations of experiments consisting of ten rat measurements (*p < 0.05, ***p < 0.001; *n* = 10). Reproduced from ref. 114. With permission from [Elsevier], copyright [2019]. (C) Scheme representing the workflow in the article, starting from the synthesis of sulfoethyl chitosans (SECSs), their application as capping and reducing agents for silver nanoparticles (SECS@Ag), and the assessment of the products in anticoagulant assays. Adapted from ref. 115. With permission from [Dovepress], copyright [2018].

Cardiovascular illnesses, such as arterial or venous blood clots, are identified by the World Health Organization (WHO) as the primary cause of human death around the globe.^31,111^ These conditions can lead to acute coronary syndrome or venous thromboembolisms. Coagulation is a crucial component of physiological hemostasis, which is the process of transforming blood from a liquid state to a non-flowing gel state, which is necessary to halt the flow of blood within a damaged artery wall, particularly when there are abnormal vascular conditions or exposure to nonendothelial surfaces at sites of vascular injury.^112,113^ Blood coagulation is an intricate process that categorized into three main portions, the activation of the prothrombin, the transformation of prothrombin into thrombin, and the transformation of soluble fibrinogen in the plasma into insoluble fibrin [Fig. 7A].^44^ The coagulation activity process is generalized as a “waterfall” or “cascade” due to the sequential activation of clotting factors. The factors are then assigned a Roman numeral labeling (based on the order of their discovery), with a “a” in lowercase indicating the active version.^116^ The process of blood coagulation can be delayed or halted by employing either endogenous or exogenous anticoagulants, which deactivate or impede the function of coagulation factors.^113^ The coagulation process can be categorized into two primary pathways that initiate the blood coagulation cascade, known as the extrinsic tissue factor (TF) pathway and the intrinsic contact pathway. The TF pathway is primarily responsible for initiating the process of blood clotting in normal hemostasis, while the contact pathway is typically activated when blood comes into touch with artificial surfaces. The two paths ultimately merge at the point when the last stages of coagulation are known as the common pathway [Fig. 7A].^5^ The primary distinction between the intrinsic and extrinsic pathways in blood coagulation lies in the fact that the intrinsic pathway is triggered upon the exposure to endothelial collagen, which is exclusively revealed during instances of endothelial injury.^116^ On the other hand, the second route is triggered by the release of tissue factor by endothelial cells following external injury. Traditional approaches for identifying medications having anticoagulant effects involve assessing the activated partial thromboplastin time (APTT), prothrombin time (PT), and thrombin time (TT) assays. APTT assays employ brain lipids and activators, rather than platelets, to identify VIII, IX, XI, and excitatory releasing enzymes. This approach mimics the intrinsic route and accurately reflects the impact of natural variables on coagulation time. PT assays are performed by introducing thromboplastin into plasma to assess the impact of external stimuli on coagulation time and evaluate the functionality of the TF pathways. The TT assay is a straightforward screening test for fibrin polymerization. It assesses the activity of fibrinogen during the final stage of clotting in the common pathway. This is done by determining the time takes for fibrin to develop from fibrinogen when a certain amount of thrombin is added to plasma. The increasing in TT signifies the inhibition of thrombin.^5,31,117^ One disorder of blood coagulation results in an increased risk of elevated susceptibility to clot formation. Thus, it is necessitating to provide anticoagulants drugs.^74^ Sulfated polysaccharides possess anticoagulant activity, which is a result of the formation of the SPs/protease protein complex and the subsequent non-specific polar interaction between negatively and positively charged groups in the polysaccharide and protein. The primary mechanism of the anticoagulant effect is the inhibition of thrombin through heparin cofactor II, with the effectiveness varied depending on the specific molecule.^41^ Since 1940, heparin has been the primary employed anticoagulant/antithrombotic medication for routine surgical procedures to manage bleeding. Heparin and low molecular weight heparin are types of sulfated polysaccharides. These polysaccharides have a negative charge, which allows them to interact with various protein components in the coagulation cascade. This connection enables heparin to decrease the activity of thrombin.^118,119^ Heparin primarily exerts its anticoagulant effects by binding to and activating antithrombin III (AT), which then inhibits factor FXa and thrombin at critical points in the coagulation process. The interaction between heparin and antithrombin (AT) is a notable example of a highly specific interaction between sulfated polysaccharides and proteins. This interaction is characterized by a pentasaccharide sequence that is minimally variable and is present in approximately 30% of the polysaccharide chains in unfractionated heparin. Heparin enhances the activity of the thrombin inhibitor heparin cofactor II (HCII). In contrast to antithrombin (AT), heparin cofactor II (HCII) exhibits poorer specificity for heparin and can interact with other sulfated polysaccharides. However, HCII inhibits thrombin at a slower pace and does not inhibit factor Xa. Although heparin is highly effective as an anticoagulant and antithrombotic medication, there have been and continue to be difficulties associated with its animal-derived nature. These include diseases that reduce the availability of animal resources and the potential for contamination.^5^ Nevertheless, both heparin and LMWH can induce serious side effects, including a heightened risk of bleeding. This is mostly due to their selective targeting of coagulation components in the common pathway of the coagulation cascade, which directly impacts hemostasis. Additionally, the presence of thrombocytopenia, hemorrhagic impact, and congenital or acquired antithrombin deficiency might lead to invalidation.^74,111,119^ Furthermore, the extraction of this substance from animal tissue may lead to hypotension and the presence of additional animal diseases.^119^ Furthermore, heparin has been associated with certain lethal conditions. Liu *et al.* recently published a paper indicating that thrombocytopenia caused by heparin poses a significant risk of mortality in critically ill COVID-19 patients receiving heparin treatment.^120^ Consequently, the drawbacks of heparin have resulted in a need for novel alternative sources of anticoagulants that are both safer and more efficient.^112^ Sulfated polysaccharides derived from non-animal sources have the potential to be used as anticoagulant agents. Moreover, altered SPs have the capacity to function as an anticoagulant by restraining thrombin, stimulating antithrombin III, or prolonging the timeframe of intrinsic and extrinsic pathways. Sulfated polysaccharides (SPs) are seen as effective alternatives to heparin because of their considerable structural variability, enabling them to function differently from heparin. Additionally, SPs reduce the risk of viral particle contamination. The sulfate group engages in an interaction with antithrombin III which lead to hinder the clotting process of serine proteases that have the ability to convert prothrombin into thrombin as well as, hinder the activity of thrombin (an enzyme responsible for converting fibrinogen into fibrin through the polymerization of fibrin by transglutaminase), thus initiating the process of blood clot formation.^121^ The anticoagulant activity of sulfated polysaccharides (SPs) is determined by their structural characteristics,^111^ including their molecular weight (Mw) and the degree and distribution/position of sulfate groups.^112,122^ Regardless of chemical regioselectivity, sulfation at C2 of 3,6-anhydro-α-d-Galp and C6 of β-d-Galp have a positive impact on anticoagulant activity *in vitro* as stated by de Araújo *et al.*^42^ In addition, enhancement can be achieved through oversulfation. Typically, anticoagulant activity increases gradually as the degree of sulfation of the polysaccharides increases.^5^ Greater sulfate group content correlates with increased anticoagulant activity.^123^ Moreover, modified SPs have been discovered to possess the most potent anticoagulant properties. Chen *et al.* emphasized that the skeletal characteristics of SPs are crucial for their notable *in vitro* anticoagulant activity, as demonstrated by assessments of activated partial thromboplastin time, thrombin time, and fibrinogen concentrations.^124^ Freeman *et al.* found that only heparin-binding proteins exhibited strong and specific binding to alginate-sulfate, with comparable or higher equilibrium binding constants to alginate-sulfate than to heparin.^125^ Mao *et al.* proposed that the incorporation of sulfonate/sulfate groups into the shell layer of the carbon nanowires (CNWs) enhances their anticoagulant activity by selectively blocking thrombin activity. Carbon nanowires (CNWs) produced from sodium alginate and ammonium sulfite have enhanced anticoagulant capabilities compared to conventional anticoagulants in suppressing thrombin activity, as evidenced by both *in vitro* and *in vivo* assessments. When comparing CNWs Alg@SO_*x*_-165 to the widely used sulfated polysaccharide, fucoidan, it is evident that the hybrid nanocomposite has a significantly higher anticoagulant effectiveness. This is proved through *in vitro* thromboelastography (TEG) and *in vivo* rat-tail bleeding studies. In addition, the hybrid nanocomposite demonstrate superior biocompatibility and increased potential for biological applications in comparison to anticoagulant nanoparticles based on metals [Fig. 7B].^114^ In addition, Ehmann *et al.* found that by coated gold nanoparticles into sulfated chitosan, the anticoagulant activity of the sulfated chitosan significantly improved. The sulfated chitosan-coated gold nanoparticles offer a novel approach to anticoagulant activity by leveraging high surface charge and the coordination of calcium ions (Ca^2+^) to the amine and sulfate groups of the polysaccharide shell. When the SPs nanocomposite is tested on blood samples, it considerably increases the aPTT time and PT time. In addition, SPs nanocomposite also have the potential to reduce infection risks due to their semi-synthetic nature.^126^ Similarly, Heise *et al.* found out that the introduction of sulfate groups into the chitosan backbone improves its hemocompatibility and imparts significant anticoagulant properties. The sulfated chitosan with a high degree of substitution, were found to inhibit cofactor Xa activity effectively. Additionally, SPs nanocomposite coated with silver demonstrated even higher anticoagulant activity compared to pure sulfonated chitosan due to the increased charge density and stability provided by the silver core, which prevents aggregation [Fig. 7C].^115^ Besides these, a range of SPs with antioxidant activity is listed in Table 2.

**Table 2:** List of sulfated polysaccharide and their anticoagulant activity

Type of SPs	Modification	Anticoagulant characteristics	Assays	Ref.
**Unmodified SPs**				
Brown algae (*Sargassum policystum*)	Unmodified	AT-III	PT, aPTT, TT	121
Green alga (*Cladophora oligoclada*)	Unmodified	Intrinsic coagulation pathway	PT, aPTT	122
Brown algae (*Laminaria japonica*)	Unmodified	Intrinsic coagulation pathway	aPTT, TT	123
Green algae (*Chaetomorpha aerea*)	Unmodified	HC-II, AT-III	aPTT, TT	127
Aquatic plant (*Sagittaria trifolia*)	Unmodified	AT-III	PT, aPTT, TT	128
Red alga (*Gelidiella acerosa*)	Unmodified	HC-II, AT-III	PT, aPTT	129
Lambda- and theta-carrageenan	Unmodified	HC-II, AT-III	aPTT, TT	130
Rhamnan sulfate (*Monostroma nitidum*)	Unmodified	Inhibiting tissue factor (TF)	PT, aPTT	131
Ulvan (*Ulva rigida*)	Unmodified	AT-III, Stachrom Heparin kits	PT, aPTT, TT	132
*Calocybe indica* (mushroom)	Unmodified	Extrinsic pathway of coagulation	aPTT, TT	133

**Modified SPs**				
Cellulose sulfates	Sulfated	Xa, IIa, AT-III	PT, TT	134
Sulfated glycoglucuronomannan (gum *Vochysia thyrsoidea*)	Sulfated	Xa, HC-II, AT-III	aPTT	135
Sulfonated chitosan	Sulfonated	Xa, the intrinsic pathway	PT, aPTT	136
Dermatan sulfate (starfish *Lysastrosoma anthosticta*)	Over sulfated	Xa, IIa	aPTT	137

**Hybrid SPs**				
Sulfated alginate	Sulfated-CNWs NCs	Thrombin activity	PT, aPTT, Thrombin Clotting Time (TCT), thromboelastography (TEG)	114
Sulfated chitosan	Sulfated-Au NPs	AT-III	PT, aPTT, Quartz Crystal Microbalance with Dissipation (QCM-D)	138
Sulfonated chitosan	Sulfated-Ag NPs	Xa	PT, aPTT, chromogenic determination, factor Xa activity assay	115
Fucoidan-based chitosan nanocomposites	Nanocomposites	Reduced thrombus formation	aPTT	139
Carrageenan	Resin composite-Cu NPs	Xa, IIa	aPTT	140
Heparin	Ag NPs	Blood coagulation cascade	aPTT	141
Carrageenan-based heparin-mimetic gel	Composite	Thrombin–antithrombin complex (TAT)	PT, aPTT, TT	142
Ulvan/kappa-carrageenan/carrabiose	Composite	HC-II, AT-III	aPTT	143
Chitosan-kappa-carrageenan hydrogels	Composite hydrogels	Intrinsic coagulation pathway	PT, aPTT, TT	144

#### 6.1.2 Antioxidant activities and sequestration of free radicals

**Fig. 8:**
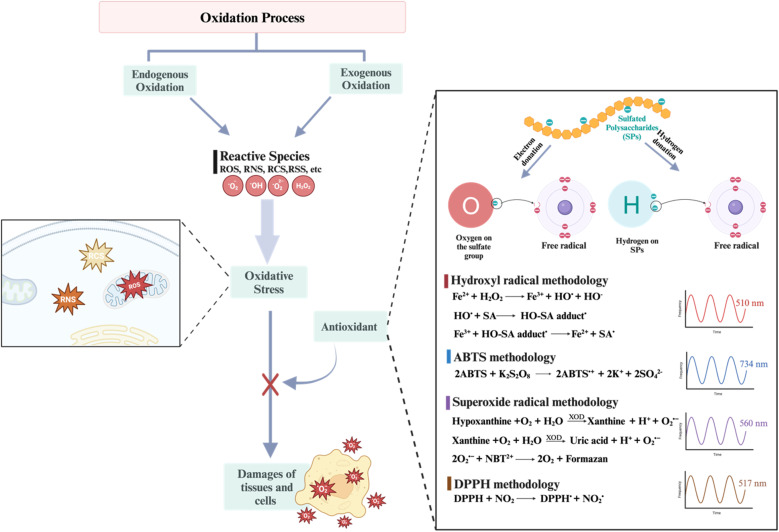
Simple roadmap of oxidation process and mechanism of the antioxidant activity of SPs. Created with https://www.BioRender.com/.

Oxidation is an ordinary physiological process that occurs in the human body. It is essential for energy production, metabolism, and the immune response, as well as many biochemical processes, including cell survival, proliferation, and cell signaling.^145^ Oxidation mechanisms involve the transfer of hydrogen, oxygen atoms, or electrons from one species to another. During this transferring process, free radical species are produced as natural byproducts, which are unstable and highly reactive chemical species as they have unpaired electrons in their outer shell. In normal conditions, free radicals control cell growth and prevent the growth of viruses and bacteria, therefore lowering the risk of infection. However, excessive free radicals have the ability to harm tissues and cells by attacking intracellular proteins, lipids, nucleic acids, and T cells, (which can result in diminished immune function), loss of membrane integrity, altered cellular processes, and mutations.^145,146^ Furthermore, it heightens the risk of developing a range of illnesses, including cancer, cardiovascular and cerebrovascular diseases, diabetes, inflammatory conditions (such as arthritis), hypertension, neurodegenerative diseases (*e.g.*, Parkinson's or Alzheimer's), premature aging, and other chronic diseases.^145^ In addition to disrupting the balance of redox homeostasis, this leads to oxidative stress.^32^ The following are the essential phases in the mechanics of the oxidation byproduct [Fig. 8]:

• The generation step: involves the formation of free radicals associated with reactive species such as ROS, RNS, RSS, RCS, and RSeS. These reactive species are produced endogenously through metabolic processes within the cells of our body or exogenously from sources like high-ionizing radiation, drugs, and environmental toxins (pollution, heavy metals, pesticides, smoke, *etc.*).^146,147^ The term reactive species refers to two classes of molecules: free radicals (with an unpaired electron in their outermost shell) and non-radicals (without an unpaired electron but still having chemical reactivity). Reactive oxygen species (ROS) are unpaired radicals such as superoxide anion (O_2_˙^−^), nitric oxide (NO˙), hydroperoxyl (HO˙), peroxyl 
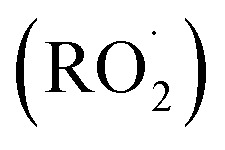
, and hydroxyl (˙OH) radicals; non-radical ROS are chemically reactive and can even be changed to radical ROS, *e.g.*, singlet oxygen (^1^O_2_), ozone (O_3_), peroxides (ROOH), hydrogen peroxide (H_2_O_2_), and hypochlorous acid (HOCl). Reactive nitrogen species (RNS) consist of nitric oxide (NO˙) and nitrogen dioxide 
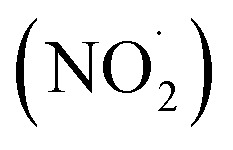
, peroxynitrite (HNO_3_) radicals, as well as other oxides of nitrogen. Reactive sulfur species (RSS), which include hydrogen sulfide (H_2_S), thiols (RSH), persulfides (RSSH), *S*-nitrosothiols (RSNO), and sulfenic acids (RSOH).^147^

• The propagation step: involves the reactivity of free radicals with other molecules in a chain reaction. As an illustration, a free radical has the ability to interact with a lipid molecule within a cell membrane, creating a lipid radical, which can then react with another lipid molecule, leading to the propagation of the chain reaction.^147^

• The oxidative stress step: refers to the state of imbalance between the production of free radicals and the body's ability to defend against these reactive species, with a bias towards the oxidants.^146,147^

An antioxidant is a compound that hinders the oxidation process by impeding reactive species and counteracting free radicals with the donation of an electron to stabilize them.^146^ The body naturally produces antioxidants, which can also be obtained from external sources such as food and nutritional supplements. In addition, there is another category of antioxidants that can be artificially manufactured.^147^ The antioxidant defense mechanisms can be categorized into two primary types: enzymatic and non-enzymatic. The enzymatic antioxidants consist of superoxide dismutase, catalase, ascorbate peroxidase, and glutathione reductase. Non-enzymatic antioxidants encompass a variety of organic molecules, including polysaccharides, polyphenols, peptides, vitamins, and other substances.^146^ The suppression of lipid peroxidation, augmentation of antioxidant enzyme activity, elimination of nitric oxide, and binding of metal ions (such as iron, cadmium, lead, mercury, copper, *etc.*) can otherwise accelerate the generation of reactive oxygen species *via* Fenton-type processes. These systems aid in preserving the structural integrity of cells and mitigating oxidative damage and inflammation.^148^ Antioxidant activity can be evaluated using various *in vitro* methods, such as DPPH (2,2-diphenyl-1-picrylhydrazyl) scavenging assays, ORAC (oxygen radical absorbance capacity), ABTS (2,2-azino-bis(3-ethylbenzothiazoline-6-sulfonic acid)), FCA (ferrous ion chelating ability), FRAP (ferric ion reducing antioxidant power), and ROS (reactive oxygen species) scavenging assays.^58,72,112,116^ These methods involve chemical assays that measure the ability of antioxidants to neutralize free radicals. Furthermore, biochemical techniques such as the oxidation of low-density lipoprotein (LDL) assay and the thiobarbituric acid reactive substances (TBARS) assay can be employed. However, the first options are favored above the second options because of their straightforwardness, efficiency, and affordability.^116^ The DPPH assay relies on the antioxidant's ability to donate a hydrogen atom to the synthetic nitrogen radical molecule DPPH.^58^ The ORAC assays quantify the capacity of antioxidants to disrupt radical chains by observing their ability to prevent oxidations generated by peroxyl radicals. Trolox is commonly employed as a reference antioxidant, and trolox equivalents (TE) are correspondingly quantified as the ORAC values of the antioxidants being tested.^58^ The FRAP assay quantifies the ability of an antioxidant to convert Fe^3+^ to Fe^2+^ in an acidic environment (pH 3.6) using 2,3,5-triphenyltetrazolium chloride (TPTZ) as a stabilizer. The antioxidant capacity is determined by measuring the absorbance at 593 nm, which is attributed to the blue hue of the ferrous (Fe^2+^) complex. The antioxidant capacity is quantified by expressing it as the value of Fe^2+^ or reference standard antioxidants, such as Trolox.^58^ Artificial sources of antioxidants have several disadvantages, such as the potential to cause cancer and harm the liver. Due to these factors, the pursuit of an alternative natural antioxidant has become a crucial objective in pharmacological research.^30^ Sulfated polysaccharides are highly favorable options because of their safety, reduced toxicity, biocompatibility, and, notably, their potent antioxidant properties that can safeguard the human body from free radicals and mitigate the issues associated with many diseases.^112^ SPs exhibited their antioxidant properties by effectively binding to metals and donating hydrogen atoms. This can be attributed to the sulfate group acting as an electrophile, which facilitates the removal of hydrogen atoms from the anomeric carbon and prevents the formation of free radicals by neutralizing them.^5,28^ Furthermore, the presence of the sulfate group promotes the water solubility of polysaccharides, improving their interaction with free radicals and increasing their ability to scavenge them. The enhanced capacity to scavenge hydroxyl radicals can be due to the strong nucleophilic nature of the SO_3_^−^ group. The polysaccharide derivatives exhibit high DPPH scavenging capabilities, which can be attributed to an enhancement in their proton donating capability. Moreover, the enhancement in FRAP characteristics of polysaccharide derivatives can be attributed to their reductive capacity, ability to hinder ongoing hydrogen abstraction, decomposition of peroxide, binding with transition metal ions to catalyze reactions, prevention of radical scavenging and chain initiation, augmentation in the electron cloud density of active hydroxyl groups, and an increase in electron-donating capability.^72^

Many factors play a key role in their ability to effectively determine the antioxidant activity of sulfated polysaccharides, including their structural characteristics, molecular weight (Mw), sulfate content, the position of sulfate groups, monosaccharide residue content and composition, the extraction methods, and the solvents used to extract the SPs.^58^ The position of sulfate groups on the polysaccharide backbone has been demonstrated as antioxidant potential of sulfated polysaccharides. For instance, sulfated polysaccharides with sulfate groups at the C-6 position of glucan units typically exhibit higher antioxidant activities than those with sulfates at other positions.^149,150^ Structure and sulfation patterns may influence the antioxidant effects of SPs. It has been reported that ι-carrageenan demonstrates higher hydroxyl radical scavenging than λ and κ-carrageenan.^112^ This indicates that not only sulfate content but also the spatial patterns of sulfate groups determine the antioxidant activity of polysaccharides.^58^ The molecular weight of polysaccharides is another important factor affecting their antioxidant activity. It was observed that the SPs showed strong antioxidant activity. The lower Mw incorporated into the system more efficiently and donated protons to scavenge free radicals more effectively compared to the high Mw SPs.^112^ It seemed that shorter chains form fewer intramolecular hydrogen bonds, and therefore the reactive groups are more accessible, contributing to the radical scavenging activity.^109^ The degree of sulfation, or sulfate content, plays a pivotal role in antioxidant activity because of its electron donation potential, which can neutralize free radicals. The density and pattern of sulfates in the polymer are also important for their antioxidant activity. Higher sulfate density and a specific pattern of distribution enhance the ability of molecules to access reactive species and neutralize free radicals.^10^ SPs have more efficient antioxidant activity when compared to non-sulfated polysaccharides, around 65 to 75%.^28^ For instance, *Sagittaria trifolia* (PST) and its sulfated derivatives were evaluated using hydroxyl and DPPH radical-scavenging assays. The results indicated that SPST-4, with a lower DS but smaller MW, demonstrated stronger antioxidant activity than SPST-3, which had a higher DS but larger MW. This indicates that other structural parameters, such as degree of branching, glycosidic linkage, and conformation, also play a role in determining the antioxidant potential of sulfated polysaccharides.^128^ In addition, sulfation modifications improve the antioxidant characteristics by increasing the sulfate content. This leads to the activation of the hydrogen atom of the anomeric carbon, resulting in an enhanced hydrogen atom-donating capacity.^151^ Solvents and extraction methods can change SPs molecular structure, which in turn influences the antioxidant properties of SPs. For example, gamma radiation has been shown to decrease the molecular weight of fucoidans extracted from seaweed, boosting their antioxidant activity. Advanced extraction methods like ultrasound and microwave-assisted extraction have been explored as ways to improve the yield and antioxidant properties of sulfated polysaccharides, as they can help preserve the integrity of the molecular structure.^149,152–154^ Furthermore, green extraction methods, like enzyme-assisted and pressurized liquid extraction, preserve metabolite integrity and activity.^155^ Deep eutectic solvents, a new type of green solvent, have demonstrated superior extraction efficiency and isolation compared to conventional hot water extraction. Overall, the solvents and extraction method determine the physicochemical properties of SPs, such as molecular weight and sulfate content, which in turn significantly influence their antioxidant activities. Careful optimization of these parameters can lead to the isolation of SPs with enhanced antioxidant activity.^149,156^ Chitosan contains both amino and hydroxyl groups, which have the capacity to interact with free radicals and demonstrate scavenging properties. However, sulfated chitosan demonstrates enhanced antioxidant activity. This confirms that the activities and mechanisms of polysaccharide derivatives depend on the method of modification and the type of polysaccharide. The superior performance of a certain derivative, in comparison to others, can be attributed to its increased solubility, smaller distribution of molecular weight, reduced intrinsic viscosity, and hyperbranched conformation.^72^ SPs have the ability to interact in a synergistic manner with other antioxidant medications or substances, hence increasing their effectiveness.^10^ Antioxidants derived from SPs not only possess scavenging abilities against these reactive radicals but also inhibit the proliferation of cancer cells while simultaneously protecting normal cells and impeding further tumor growth. Furthermore, it promotes the efficacy of chemotherapy medications and improves the quality of life for cancer patients by mitigating adverse effects.^147^ Chen *et al.* found that enhanced chemically sulfated polysaccharide might reduce the formation of kidney stones. The study demonstrates that the sulfate content not only influences the high antioxidant level but also protects the cells from harm caused by the CaO_*x*_ nanocrystal. In this investigation, the sulfur trioxide-pyridine approach was employed to sulfated *Undaria pinnatifida* polysaccharide (UPP0). The results showed an improvement in cell survival, preservation of cell morphology, and a decrease in ROS generation, Δ*Ψ*_m_ reduction, and adhesion. There was a decline in the expression of adhesion proteins (ANXA1) and the eversion of the attachment molecule PS. In addition to preventing nano-COM adherence, UPP also blocks cell death and necrosis. HK-2 cells were protected against nano-UPP1-UPP3 with –OSO_3_ concentrations of 6.03%, 20.83%, and 36.39% by means of UPPs. The antioxidant activity and capacity to control the formation of CaO_*x*_ crystals were shown to be most strongly correlated with the –OSO_3_ concentration of the UPPs, with UPP3 exhibiting the highest levels.^157^ Yim *et al.* examine the prevention of SARS-CoV-2 viral entrance by crude polysaccharides derived from seaweeds and abalone viscera *in vitro*. Bioactive polysaccharides derived from edible seaweeds and abalone viscera may be investigated as therapeutic treatments for obstructing COVID-19 entrance through additional investigations involving the purification and identification of bioactive components.^158^ The anticoagulant activity was contingent upon the regiochemistry of the sulfate groups inside the polysaccharide backbone. Through chemical changes (oxidation), dos Santos-Fidencio *et al.* engineered theta- and lambda-carrageenan. The presence of sulfate groups at C2 of β-d-galactopyranose units exhibited superior anticoagulant efficacy compared to those at C4. Moreover, partial oxidation of kappa-carrageenan, as opposed to complete oxidation, had a superior anticoagulant action.^159^ Other interested antioxidant activity studies are listed in Table 3.

**Table 3:** List of sulfated polysaccharide and their antioxidant activity

Sulfated polysaccharide	Modification	Antioxidant activity	Efficacy (*in vitro*)	Antioxidant properties	Ref.
**Unmodified SPs**					
Brown algae (*Sargassum policystum*)	Unmodified	FRAP	IC_50_ = 91.306 ppm	Sulfate group	121
λ- and θ-carrageenan	Unmodified	FRAP	0.8 mM	Lower Mw, positions and density of sulfate groups	130
HMw λ-carr		FC	Inhabitation rate: 20% GAE		
LMw λ-carr			1.5 mM inhabitation rate: 35% GAE		
HMw θ-carr			2.8 mM inhabitation rate: 60% GAE		
LMw θ-carr			1.2 mM inhabitation rate: 30% GAE		
*Calocybe indica* (mushroom)	Unmodified	DPPH	EC_50_ = 1.99–3.82 mg mL^−1^	Concentration-dependent	133
			0.78–2.78 mg mL^−1^ for reducing ability		
			4.11 mg mL^−1^ for metal chelating		
			0.56–4.18 mg mL^−1^ for lipid peroxidation		
Porphyra yezoensis (DPY-1)	Unmodified	DPPH	DPY-1: IC_50_ = 1.82 mg mL^−1^	Highest sulfate group content the higher antioxidant	148
(^−^OSO_3_H content 17.9%)			DGL-2: IC_50_ = 8.24 mg mL^−1^		
*Gracilaria lemaneiformis* (DGL-2)			DSF-3: IC_50_ = 9.68 mg mL^−1^		
(^−^OSO_3_H content 13.3%)			DUP-4: IC_50_ = 14.6 mg mL^−1^		
*Sargassum fusiform* (DSF-3)					
(^−^OSO_3_H content 8.2%)					
*Undaria pinnatifida* (DUP-4)					
(^−^OSO_3_H content 5.5%)					
Brown algae (*Sargassum horneri*)	Unmodified	DPPH	Inhabitation rate: 32.36% (at concentration 0.5 mg mL^−1^), 67.82% (at concentration 3 mg mL^−1^)	DESs-assisted extraction (green method)	156
*Caulerpa lentillifera* (sea grape algae)	Unmodified	DPPH	SGP_11_ ≃ 32.75	Low molecular weight, high sulfate content	160
SGP_11_ = 12.20			SGP_21_ ≃ 22.58		
SGP_21_ = 18.20			SGP_31_ ≃ 18.41		
SGP_31_ = 21.80		ABTS	SGP_11_ ≃ 5.39		
			SGP_21_ ≃ 1.02		
			SGP_31_ ≃ 1.28		
		DPPH	IC_50_ = 0.15 mg mL^−1^		
		ABTS	IC_50_ = 0.12 mg mL^−1^		
		NO	IC_50_ = 0.18 mg mL^−1^		
Brown algae (*Padina boryana*) *P. boryana* (PBE) enzyme assisted extract (SO_3_^−^ content 42.14%) *P. boryana* (PBP) ethanol precipitated, (SO_3_^−^ content 56.34%)	Unmodified	DPPH	PBE: IC_50_ = 4.26 ± 0.14 mg mL^−1^	Highest sulfate group content the higher antioxidant	161
			PBP: IC_50_ = 3.66 ± 0.44 mg mL^−1^		
Red algae (*Gracilaria blodgettii*)	Unmodified	DPPH	Inhabitation rate: 19.80%	Sulfate group	162
		HO	Inhabitation rate: 8.80%		
		ABTS	Inhabitation rate: 25.42% (2 mg mL^−1^)		
Brown algae (*Sargassum horneri*)	Unmodified	The superoxide radical assay, hydroxyl radical assay, ABTS, DPPH, FRAP	- 65.0% (SHP80), 64.5% (SHP60) and 35.0% (SHP30) at cons 2.5 mg mL^−1^	Lower Mw, highest sulfate group content	163
			- 47.57% (SHP80), 85.56% (SHP60) and 98.07% (SHP30) at cons 2.5 mg mL^−1^		
			- Above 94% at low cons of 1.5 mg mL^−1^		
			- 85.01% (SHP80), 73.96% (SHP60) and 71.74% (SHP30) at cons of 2.5 mg mL^−1^		
			- Absorption of 1.78 (SHP30), 1.73 (SHP60), and 0.71 (SHP80) at cons 2.5 mg mL^−1^		

**Modified SPs**					
Plant (*Cyclocarya paliurus*)	Sulfated	DPPH	IC_50_ = 36.22% at 4 mg mL^−1^ (nonsulfated)	Sulfated at the C6 position, exhibit enhanced antioxidant activity	149
			IC_50_ = 5.59 mg mL^−1^ (sulfated)		
			IC_50_ = 44.14% at 4 mg mL^−1^		
			IC_50_ = 3.14 mg mL^−1^		
Brown algae (*Enteromorpha prolifera*)	Sulfated	DPPH	IC_50_ = 0.713 mg mL^−1^	Lower mw, sulfate group	151
			93.26% (at concentration 10 mg mL^−1^)		
Sulfated *Undaria pinnatifida* (UPPs)	Sulfated	The ˙OH removal capacity of the UPPs	UPP3 (63.98%) > UPP2 (50.16%) > UPP1 (40.53%) > UPP0 (20.34%)	Highest content of –OSO_3_, Mw	157
UPP1–UPP3 with –OSO_3_ contents of 6.03%, 20.83%, and 36.39%				Inhibiting HK-2 cell, antioxidant activity, optimal cell protection ability and crystal adhesion inhibition ability	
Collagen/chitosan	Complexes	Malonic dialdehyde (MDA)	7.61 ± 1.43 nmol mL^−1^	Good antioxidant activity *in vivo* (ten-month old mice)	164
		Superoxide dismutase (SOD)	6.77 ± 0.33 × 103 Nu mL^−1^		
*Sargassum pallidum*	Sulfated	DPPH and ABTS	- 91% (SPP), 96.53% (S-SPP_1–4_), 92.04% (S-SPP_1–6_) and 89.33% (S-SPP_1–8_) at cons from 0.25 to 2 mg mL^−1^	- Moderate DS had much better DPPH radical scavenging activity	165
			- 77.6% (SPP), 78.1% (S-SPP_1–4_), 70.1% (S-SPP_1–6_) and 70.8% (S-SPP_1–8_) at cons from 0.5 to 1.0 mg mL^−1^	- A low DS had better ABTS radical scavenging activity at the low concentrations	

**Hybrid SPs**					
*Dictyota mertensii*	Ag NPs	DPPH	95.1± at 0.25 mg mL^−1^	- High antioxidant capacity	166
				- Nanoparticles enhanced the efficacy of polysaccharides as scavengers for reactive species	
Fucoidan/chitosan	Copolymer	DPPH	Up to 80%	- Exhibited highly potent antioxidant effects	167
			Fucoidan concentration was higher than 0.31 mg mL^−1^	- DPPH of CS/F NPs primarily derives from fucoidan	
	Nanoparticles (ChS/F NPs)		=Up to 90%		
κ-Carrageenan	Multilayer coating	DPPH	31.32 ± 3.13%	Good antioxidant activity	168
	Quercetin nanoparticles lecithin/chitosan (NPs)	FRAP	99.41 ± 95.39 μM		
Carrageenan (Car) and fucoidan (Fuc)	Fuc/Car co-stabilized protein-isolated (SPI)/curcumin (Cur, C) loaded nanoparticles	DPPH	(Significant decrease in SC_50_ (*p* < 0.05) and remarkable increase (*p* < 0.05) in the reducing power)	Dramatically enhancing the stability and antioxidant activity of curcumin	169

#### 6.1.3 Immunomodulating and anti-inflammatory activities

**Fig. 9:**
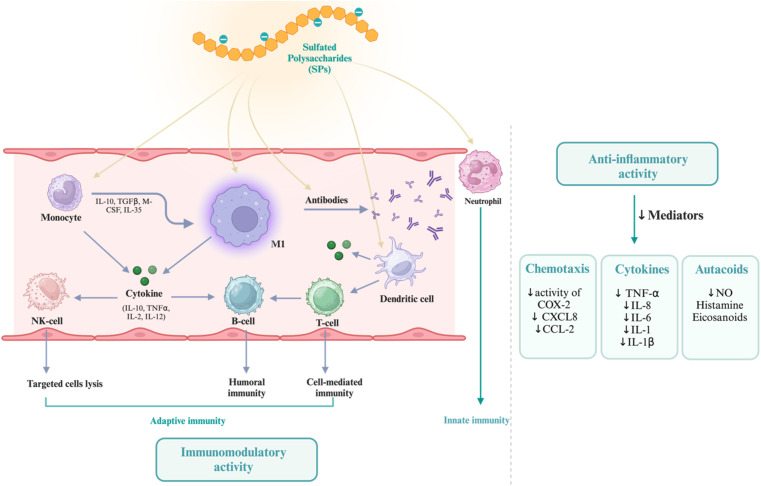
Immunomodulatory mechanism and the anti-inflammatory activity of SPs. Created in BioRender. Alfinaikh R. (2025) https://BioRender.com/r26t839.

Immunostimulation is considered an essential defense system of the body to mitigate the adverse consequences of burns and exposure to foreign surfaces and to prevent and combat infections, inflammatory illnesses, and cancer.^72,145^ Immunomodulation is a treatment approach that adjusts the levels of cytokines in the human body, either by reducing inflammation and regulating immunological responses or by enhancing a weakened immune system. A variety of cytokines control the activation, growth, multiplication, elimination of natural killer cells (NK cells), and their movement towards specific targets.^119^ Polysaccharides primarily modulate immunity through two primary mechanisms. There are two approaches to treating cancer: direct elimination of cancer cells and boosting the immune system, for example, by strengthening the functions of macrophage cells and T lymphocytes. Ultimately, polysaccharides have the potential to stimulate the production of immunological components, which in turn could improve an organism's immune function.^5,44^ When it comes to immunological responses, there are two categories: innate immunity and adaptive immunity. Cells that are engaged in innate immunity, such as dendritic cells (DCs), natural killer (NK) cells, monocytes, and macrophages, play a crucial role in innate immunity, whereas T lymphocytes and B lymphocytes are essential for adaptive immunity. Macrophages are recognized as the primary participants in the innate immune system, and chronic inflammation occurs when inflammatory stimuli persist, with macrophages being the main driving factor. Nevertheless, macrophages can also exhibit anti-inflammatory properties and play a crucial role in resolving inflammation and promoting tissue regeneration. The various functions are usually facilitated by distinct macrophage subsets, initially known as M1 (proinflammatory) and M2 (anti-inflammatory) macrophages. These subsets are regulated by communication with other cells and the presence of pro- or anti-inflammatory cytokines in the surrounding environment. ESPs can potentially function as coreceptors or antagonists in the control of the immune response by interacting with these cytokines.^5^ Sulfated polysaccharides (SPs) found in marine algae have the ability to modify the immune system, which could be useful in enhancing immune response or regulating the activity of immune cells to reduce negative consequences like inflammation.^88^ Research has demonstrated that both natural polysaccharides and chemically modified derivatives have immune-modulatory properties that could be useful in enhancing the immune response or regulating immune cell activity. These substances can influence the function of the immune system through various pathways.^5,88^ Sulfation is a widely studied modification of polysaccharides that has been found to have significant immunomodulatory effects. Numerous studies have demonstrated that sulfation enhances the immunomodulatory capabilities of these polymers. Furthermore, scientific evidence has shown that the elimination of sulfate groups from naturally occurring sulfated polysaccharides results in significantly diminished immunomodulatory action. Immunological processes are intricate, and as shown in the literature, sulfated polysaccharides may have a dual function.^5^ The stimulation can occur *via* interactions between chemicals and surface receptors, with the sulfate group being identified as having a crucial function.^34^ SPs have the ability to stimulate the activation of natural killer (NK) cells, dendritic cells (DCs), T cells, and B cells. This stimulation leads to the release of proinflammatory cytokines, which in turn can strengthen the immune system for tumor immunotherapies. Furthermore, they could suppress the production of proinflammatory cytokines, therefore demonstrating anti-inflammatory properties. An example of this is the use of fucoidan derived from *Cladosiphon okamuranus*, which has been demonstrated to enhance the activity of natural killer (NK) cells against different types of cancers. Laminarin, a polysaccharide obtained from brown algae, demonstrates substantial immunomodulatory effects. Research has demonstrated that it has the ability to enhance macrophage activity, which is essential for the immunological response.^145^ Moreover, laminarin has the ability to augment the secretion of many inflammatory mediators, including hydrogen peroxide, nitric oxide, and cytokines, suggesting its potential as an immunostimulatory agent. These characteristics indicate that laminarin may have potential in therapeutic interventions targeting the modulation of the immune system.^170^ Inflammation is a defense mechanism involved in physiological and pathologic immune system responses when infection occurs. This process is initiated by the liberation of numerous inflammatory mediators, including cytokines, chemokines, and reactive oxygen/nitrogen intermediates. Inflammation, a typical biological response, facilitates the removal of toxic substances and improves the healing of wounds.^31^ The primary objective of the inflammatory response is to direct circulating leukocytes and plasma proteins to the site of the infection or tissue injury, eliminate the causative agent, and initiate the healing process. Even though inflammation is essential for survival, it can become detrimental if it is excessively intense, fails to eliminate the underlying cause, or targets the host organism, leading to potential harm. Given its close association with the production of free radicals.^171^ Inflammatory responses involve the stimulation of macrophages by pro-inflammatory substances like lipopolysaccharide (LPS), interleukin-1β (IL-1β), interferon-γ (IFN-γ), and the nuclear factor kappa B (NF-κB). This stimulation leads to the activation of the cyclooxygenase (COX) and lipoxygenase (LOX) pathways, resulting in the production of nitric oxide (NO), tumor necrosis factor-α (TNF-α), and interleukin-6 (IL-6) primarily. The anti-inflammatory capabilities of natural compounds are investigated *in vitro* by studying these pro-inflammatory mediators. Regarding anti-inflammatory treatments, non-steroidal medications have demonstrated the ability to impede the formation of arachidonic acid and decrease prostaglandin levels. This contributes to the reduction of NO, which is linked to tissue toxicity, various inflammatory disorders, and carcinomas. A chemical that causes a decrease in nitric oxide (NO) levels has the potential to reduce inflammation.^116^ Algae contain a variety of compounds that have anti-inflammatory properties. Since SPs can effectively hinder the generation of nitric oxide—a key factor in cell inflammation—they have been investigated for their potential anti-inflammatory effects.^155^ The spatial arrangement and concentration of sulfate groups inside SPs play a crucial role in determining its effectiveness in reducing inflammation. Increased sulfation yields a greater abundance of negatively charged molecules that interact with proinflammatory molecules, while the uniform distribution of these sulfate groups ensures a reliable anti-inflammatory effect. The sugar content is a significant determinant of the anti-inflammatory effects. The sulfate pattern of each SPs substantially influences its affinity to important inflammatory proteins. The spatial distribution of sulfate groups on sugar units, including their specific location and proximity to adjacent sulfates, results in anti-inflammatory properties. The anti-inflammatory activities of SPs are significantly influenced by their molecular weight, according to previous investigations. It is hypothesized that smaller fragments interact with inflammatory mediators more effectively due to improved tissue penetration and increased bioavailability. The branching and sulfation pattern of SPs affects its binding to cell receptors, which in turn regulates signaling pathways. To achieve the best anti-inflammatory results, it is crucial to carefully evaluate the placement of sulfate groups and branching points. The shape, size, and net charge of polysaccharides are crucial determinants of their binding affinity to inflammatory mediators. Greater molecules may possess more expansive binding sites, whereas the distribution of charge influences the electrostatic interactions with inflammatory agents. SPs modulate immunological responses by exerting an influence on the synthesis of both proinflammatory and anti-inflammatory cytokines. Attaining equilibrium between these reactions is crucial in order to prevent excessive inflammation and immunological suppression. Gaining a comprehensive understanding of how SPs disrupt this pathway is crucial for maximizing their anti-inflammatory efficacy. Moreover, enhancing the structural characteristics of SPs can result in the creation of very effective anti-inflammatory substances that can be used for medicinal purposes. Additional research should be undertaken to identify these structural characteristics in order to selectively target certain inflammatory pathways and achieve the intended anti-inflammatory results while limiting potential adverse effects.^10^ Polysaccharides derived from nature have been extensively employed in nanomaterials to regulate inflammatory diseases. One of these mechanisms may explain why polysaccharides have an anti-inflammatory effect; for example, TCM polysaccharides primarily inhibit inflammation by reducing expression of chemotactic and adherence factors and by reducing the activity of important enzymes involved in inflammation. Sulfated polysaccharides derived from algae demonstrate their anti-inflammatory properties by interfering with the migration of leukocytes to sites of inflammation. Additionally, they inhibit the production of inflammatory-related mediators such as cytokines (IL-1b, IL-6, and TNF-a) and nitric oxide (NO) and reduce the infiltration of inflammatory cells.^30^ The human immune system plays a crucial and significant part in the body's immunological response to viral infections. Hence, contemporary research should prioritize the discovery of novel methods that enhance the immune response in the host by bolstering and advancing the immune system. The adaptive immune system requires immune stimulants and immune regulatory factors for activation. Investigation has demonstrated that polysaccharides present in certain foods like mushrooms, yeasts, algae, fruits, and cereals possess significant biological activity. These polysaccharides have the ability to activate immune system functions and stimulate the production of anti-inflammatory and antioxidant substances. Polysaccharides can serve as an immunostimulant, mitigating the harm inflicted by pathogens like SARS-CoV-2.^118^ Yang *et al.* suggest that the sulfated polysaccharide *Dictyosphaeria cavernosa* (DCS1), characterized by a unique structure and examined *in vivo*, may serve as a promising immunomodulatory drug. DCS1 kept the internal milieu stable, boosted the development of immune organs, and dramatically raised numbers of immune cells and immunological mediators.^172^ It is noteworthy that *Cyclocarya paliurus* polysaccharides (CPP) and the sulfation derivative (S-CPP) were shown to modulate the gut microbiota in immunosuppressed mice, hence enhancing systemic immunity. The findings indicated that CPP and S-CPP significantly mitigated and enhanced intestinal villi damage. In addition, they enhanced immunity in immunosuppressed mice by promoting the repair of the intestinal mechanical barrier and upregulating the intestinal microbiota, which in turn increased the expression levels of IL-1β and TNF-α in serum and small intestinal tissue, as well as ZO-1, Occludin, and Claudin-1 at gene and protein levels. There was theoretical support for the idea that sulfated modification could enhance CPP's protective effects on the intestinal mucosal barrier and regulate intestinal immunity, leading to S-CPP having a stronger ability to mitigate Cy damage. This could pave the way for the creation of S-CPP immune supplements.^173^ The immunomodulatory activity of U. conglobata Kjellman (UCP) was shown to be powerful in both vitro and *in vivo*, according to Cao S. *et al.* The phagocytotic capacity, macrophage activation, and lymphocyte proliferation might all be enhanced by UCP. Furthermore, UCP not only stabilized the internal environment but also boosted the levels of immune organ development, serum antibody levels, and peripheral blood cell counts.^174^ A highly branched fucoidan; containing sulfates at C-2 of fucose and galactose residues, obtained from the algae *Nizamuddinia zanardinii* has a well-defined immune-enhancing action on activations of RAW264.7 cells and human natural killer cells.^175^ Other similar studies of SPs with immunomodulating and anti-inflammatory activities are listed in Table 4 and Fig. 9.

**Table 4:** List of sulfated polysaccharide and their immunomodulating and anti-inflammatory activities

Sulfated polysaccharide	Modification	Assays	Properties	Ref.
**Unmodified SPs**				
*Dictyosphaeria cavernosa* (green seaweed)	Unmodified	BALB/c mice	Reduction of CD4^+^ T cells, improved the disorder of CD4^+^/CD8^+^ T cells and enhanced the immune response	172
*Ulva conglobata* Kjellman (green Alga)	Unmodified	RAW264.7 cells, BALB/c mice	Stimulate lymphocyte proliferation, activate macrophages, and improve the phagocytotic ability, increased the levels of peripheral blood cells and serum antibodies, promoted the growth of immune organs and maintained the stability of the internal environment	174
Fucoidan (*Nizamuddinia zanardinii*)	Unmodified	RAW264.7	- Activation of NF-κB and MAPKs signaling pathways of RAW264.7 and NK-92 cells	176
			- a highly branched polysaccharide with sulfates located at C-2 of fucose and ga- lactose residues	
Synthesis disaccharides that able to cross BBB	Unmodified	TLR4	6-Sulfate groups trigger TLR4	177
*D. antarctica* (Brown seaweed)	Unmodified	RAW 264.7 cells	Promoted the proliferation of spleen lymphocytes, increased NO production, enhanced phagocytic of macrophages, and NK cells	178
Hydrolyzed κ-carrageenan, ι-carrageenan and furcellaran	Unmodified	RAW264.7, HDF and HaCaT cell lines	Mw, nitric oxide (NO) production decreased	179
*Gelidium pacificum* Okamura (red seaweed)	Unmodified	THP-1 cells	Fully protected the THP-1 cells against LPS-stimulated cytotoxicity, eduction of NO production in LPS, anti-inflammatory effect *via* the TLR4 signaling pathway	180
*Cereus sinensis*	Unmodified	THP-1 cells	Inhibiting ROS generation and downregulating TLR-4 mRNA, MyD88 mRNA and TRAF-6 mRNA	181
*Sargassum swartzii* (brown algae)	Unmodified	RAW 264.7 cells	Inhibiting iNOS, COX-2, NO, and pro-inflammatory cytokines (TNF-α, IL-6, and IL-1β), nuclear transcription factors (p50/p65), MAPK signaling with TLR2/4 involvement	182
Fucoidan (*Padina arborescens*)	Unmodified	RAW 264.7 cells zebrafish models	Inhibits LPS-induced toxicity, cell death, and NO generation, pro-inflammatory cytokines (such as TNF-α, IL-6, and IL-1β), linked to the downregulation of iNOS, COX-2, MAPK, and the NF-κB signaling pathway	183
*Codium fragile*	Unmodified	RAW 264.7 cells zebrafish models	- Reduced the levels of inflammatory molecules: prostaglandin E2, nitric oxide (NO), interleukin-1 beta, tumor necrosis factor-alpha, and interleukin-6	184
			- *In vivo* test results: CFCE-PS effectively reduced reactive oxygen species, cell death, and NO levels	
Laminarin	Unmodified	Immunosuppressive mice	Promote NK cell cytotoxicity by increasing the levels of IL-12 and IFN-γ in serum and expressions of NKp30 and NKG2D, perforin and granzyme B	185
Laminarin	Unmodified	HDFa cells, NHEK cells	Modulates IL-6 secretions by cutaneous cells	186
Rhamnan sulfate	Unmodified	ApoE−/−mice	Beneficial effects in reducing inflammation, binding growth factors and NF-κB, enhancing endothelial barrier function and reducing atherosclerotic plaque formation	187
*Sargassum horneri* (brown seaweed)	Unmodified	RAW 264.7 cells	- Inhibited the activation of NF-κB p50 and p65 and the phosphorylation of MAPKs, including p38 and extracellular signal-regulated kinase	188
			- Down-regulated the production of inflammatory cytokines, including TNF-α and IL-1β	
Fucoidan (*Ecklonia maxima*)	Unmodified	RAW 264.7 cells	- Regulating pro-inflammatory cytokines (interleukin-6, interleukin-1β, and tumor necrosis factor α)	189
			- Strong NO inhibitory activity	
*Capsosiphon fulvescens* (green seaweed)	Unmodified	RAW264.7 cells	Producing considerable amounts of NO, PGE2 and cytokines	190
*Tremella fuciformis* (medicinal mushroom)	Unmodified	Colitic mice	- Growth of Foxp3^+^ T cells and the reduction of IgA-coated bacteria, which enhance anti-inflammatory cytokines and diminish pro-inflammatory cytokines	191
			- Enhanced metabolites and metabolic pathways associated with inflammatory alterations due to colitis	
*Gracilaria chouae* (red alga)	Unmodified	RAW264.7	- MTT assay indicated that GCP-3A promoted the proliferation of RAW264.7 cells	192
			- NO assay, GCP-3A significantly stimulated NO secretion	
*Sargassum fulvellum* (brown seaweed)	Unmodified	RAW264.7, zebrafish	*In vitro*	193
			- Decreased the levels of nitric oxide (NO), tumor necrosis factor-alpha, prostaglandin E2, interleukin-1 beta, and interleukin-6	
			- Inhibited the expression levels of cyclooxygenase-2 and inducible nitric oxide synthase	
			*In vivo*	
			- Improved the survival rate of LPS-treated zebrafish	
			- Reduced cell death, reactive oxygen species, and NO levels	
Chondroitin sulfate	Unmodified	ADMEcell, Caco-2 cell kits	Inhibited expression of TNF-α (at both low ∼5 mg mL^−1^ and high ∼15 mg mL^−1^ concentrations)	194

**Modified SPs**				
Sulfated *Cyclocarya paliurus* polysaccharides	Sulfated	ICR female mice	Increased the expression levels of IL-1β and TNF-α protection	173
Sulfated *Cyclocarya paliurus*	Sulfated	*Vitro* and *vivo*	Inhibiting the phagocytosis of macrophages, NO production, and the release of IL-6 and IL-1β increased the SOD activity and T-AOC level while decreasing the MDA level	195
		LPS-induced RAW 264.7 cells		
Sulfated yam polysaccharide	Sulfated	BALB/c mice	Increase the digestive enzyme activities of colon contents and restore the production of short-chain fatty acids (SCFAs) in mice, modulate the structure of the gut microbiota	196
*Cyclocarya paliurus*	Sulfated	RAW264.7 cells	Increasing the levels of cytokines secretion (TNF-α, IL-1β and IL-6), enhance the nitric oxide (NO) release	197
*Cyclocarya paliurus*	Sulfated	Dendritic cells (BM-DCs)	Determination of cytokine expression levels, p-JNK, p-p38MAPK and NF-κB p65 proteins, blocking TLR2/4	198
Sulfate carboxymethyl cellulose	Sulfated-copolymer (ionic conjugates with dialkylaminomethyl)	White mongrel male mice	Acute formalin inflammation and analgesic activity	199

**Hybrid SPs**				
Sulfated heterosaccharide	Poly(vinyl alcohol)hydrogel nanocomposite	RAW 264.7 cells	Encourage the early recruitment of macrophages and expedite the conclusion of the inflammatory stage	200
Sulfated *Ganoderma lucidum*	Selenium nanoparticles	Raw 264.7 cells	The generation of NO can be reduced by obstructing the NF-κB pathway and preventing the phosphorylation of JNK and p38 MAPK signal pathways	201

#### 6.1.4 Anti-microbial/viral activities

**Fig. 10:**
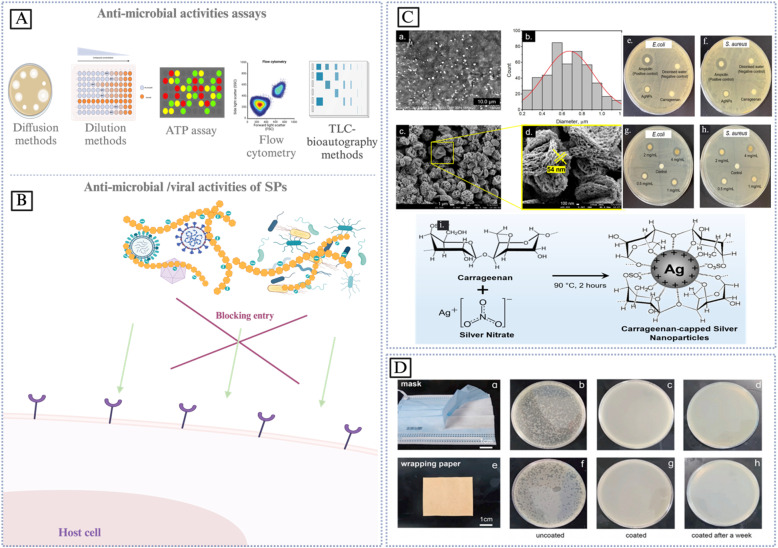
(A) Anti-microbial activity assays. (B) Direct inhabitation mechanism by inactivating microbial, virus and bacteria by SPs. Created in BioRender. Alfinaikh R. (2025) https://BioRender.com/j99m692. (C) Flower-like carrageenan-silver nanoparticles, morphological analysis of the AgNPs: (a and b) SEM image and the corresponding particle size distribution; (c and d) FE-SEM images at different magnifications; representative agar plates of the disk diffusion assay showing the antibacterial activity of carrageenan (2.5 mM) and c-AgNPs (0.1 mg mL^−1^) against (e) *E. coli* and (f) *S. aureus*; representative agar plates of the disk diffusion assay of c-AgNPs with the concentration of 0.5–4 mg mL^−1^ against (g) *E. coli* and (h) *S. aureus*; (i) proposed mechanism of the green synthesis of AgNPs by carrageenan as a reducing and stabilizing agent. Reproduced from ref. 202. With permission from [MDPI], copyright [2023]. (D) Antiviral performance of amino acids modified sulfated cellulose nanofibrils (Arg-SCNFs) for different surfaces: (a) the inner layer of a face mask and (e) wrapping paper. A comparison of the active phage-X174 plaques on a lawn of *E. coli* 8739 for the uncoated and coated surfaces are shown for each surface in panels (b–d) and (f–h), respectively. Reproduced from ref. 203. With permission from [Elsevier], copyright [2023].

Numerous viral infections pose a significant hazard to human health. Toxic side effects, drug resistance, and other limitations are common with current antiviral medications.^69^ The synergistic interaction between antibiotics and SPs against resistant bacteria may provide novel alternatives for the treatment of infectious diseases when antibiotics become ineffective.^116^ Consequently, the development of natural antimicrobial/virus medications that are both effective and safe is of the utmost importance.^34^ Polysaccharides exhibit a vast array of roles in organisms and represent an underutilized source of natural chemicals for therapeutic discovery, yet natural polysaccharides generally lack antiviral action or possess only little efficacy. Natural sulfated polysaccharides or modified SPs can enhance antiviral efficacy, particularly against HIV, with certain SPs demonstrating significant impact. The inclusion of the sulfate group typically enhances SPs activity by inhibiting viral entrance into the host cell at the initial stage, which fundamentally depends on the strong negative charge of sulfate clusters obstructing the viral adsorption of receptor cells [Fig. 10B].^28^ The antiviral mechanism of polysaccharides involves the inhibition of specific enzyme expression, hence reducing virus multiplication. Sulfated groups can augment this activity, mostly due to the potent negative charge from sulfate groups obstructing the virus's adsorption to receptor cells, hence slowing viral multiplication and diminishing viral toxicity.^44^ The initial stage of viral invasion into host cells is the binding of viral particles to the surface of the cell using electrostatic interactions. SPs may exert their antiviral actions *via* electrostatically interfering with the positively charged viral glycoprotein region and the negatively charged HS chains of the cell receptor. Several marine SPs have shown antiviral activity by interacting with virions directly, targeting the viral attachment step, or binding to receptors *via* imitating the virus-associated protein (VAP).^34^ The antiviral activity of polysaccharides is greatly affected by the density and distribution of sulfate groups along their chains. Increased sulfate density yields a greater number of negatively charged sulfate groups that engage with viral surface proteins. Another important factor in the affinity with viral surface proteins is the sugar content, equitable dispersion, and sulfate patterns. In addition, antiviral efficacy is highly dependent on molecular weight. They may have interacted more effectively with viral particles if they had been smaller, since this would have increased their bioavailability and improved tissue penetration. Replication of viruses in host cells is inhibited, and SPs disrupt various steps of the viral life cycle, according to some research.^10^ Marine sulfated polysaccharides (*e.g.*, carrageenan, alginate, fucan, laminarin, ulvan, dextran sulfate, heparin, and fucoidan) are known to exert strong antimicrobial and antiviral effects, particularly against bacterial and viral infections.^69,145^ Despite carrageenan's anti-HIV effectiveness, it is thought to have a deleterious impact on AIDS treatment because of its significant anticoagulant characteristics.^113^ Shulgin *et al.* conclude that the disaccharide repeating units, number and placement of sulfate groups plays a significant role in the antiviral activity. The research focused on CRGs (λ-κ-, X-CRG) and hybrid structure (ι/κ-CRG) with kappa and iota units. The hybrid structure contain a greater number of sulfate groups, which may account for their relatively high efficiency against human immunodeficiency virus-1 (HIV-1).^204^ According to Q. Niu *et al.*, the structure–activity relationship analysis demonstrates that the molecular weight, sulfated branches and the sulfation pattern, percentage content of sulfate groups are critical parameters for effective reduction of enterovirus 71 activity.^205^ Krylova *et al.* demonstrate that the structural characteristics of polysaccharides, particularly their highly sulfated fragments, are significant in combating DNA and RNA viruses. By comparing the antiviral efficacy of fucoidans derived from *Fucus evanescens* (FeF) and enzymatically modified fucoidans (FeHMP) both *in vitro* and *in vivo* against four different viruses: herpes simplex viruses (HSV-1, HSV-2), enteroviruses (ECHO-1), and human immunodeficiency virus (HIV-1). Both FeF and FeHMP markedly suppressed virus-induced cytopathic effects *in vitro* and showed greater efficacy against HSV. FeF demonstrated antiviral efficacy against HSV-2 with a selective index (SI) over 40, whereas FeHMP displayed a SI more than 20, whether administered prior to viral infection or during the first phases of the HSV-2 lifecycle. In addition, after being administered intraperitoneally at a dose of 10 mg kg^−1^, *in vivo* experiments demonstrated that FeF and FeHMP provided similar protection to mice against fatal intravaginal HSV-2 infection, with a percentage ranging from 44–56%.^206^ The influenza virus and coronavirus, classified as enveloped RNA viruses, are significant contributors to human respiratory illnesses. In a study, Jang *et al.* found that the lambda-carrageenan (λ-CGN) effectively blocked the action of influenza A and B viruses, with EC50 values ranging from 0.3 to 1.4 εg mL^−1^. It also inhibited the currently circulating SARS-CoV-2 virus, with an EC50 value of 0.9 ± 1.1 μg mL^−1^, and maintained no toxicity to host cells at concentrations as high as 300 εg mL^−1^. Western blot analysis and plaque titration confirmed that λ-CGN inhibited the formation of progeny viruses in culture supernatants and decreased the expression of viral proteins in cell lysates in a way that was dosage dependent. By inhibiting viral entrance and focusing on viral attachment to cell surface receptors, this polyanionic molecule has antiviral efficacy. In addition, when mice are infected with influenza, its intranasal administration Not only did a viral challenge prevent 60% of the mice from dying from the virus, but it also reduced the weight losses caused by the illness.^207^ Nasal iota-carrageenan may accelerate the recovery rate of cold symptoms by about 50% and diminish their duration by roughly 30%. A study of separate meta-analyses of nasal carrageenan's effects on adults and children was conducted by Hemilä *et al.* All colds showed a 54% improvement in recovery time when nasal carrageenan was used. In cases of coronavirus, influenza A, and rhinovirus infections, the improvement in recovery rate was 139%, 119%, and 70%, respectively.^208^ Duan *et al.* formulate films of κ-carrageenan, konjac glucomannan, and titanium dioxide nanoparticles. The films demonstrated thermal stability, UV light barrier qualities, mechanical properties, and hydrophobicity. The KC/KGM/TiO_2_ nanocomposite films (5 wt%) exhibited significant antibacterial efficacy against fungus (79%) due to the photocatalytic properties of TiO_2_ nanoparticles.^209^ Netanel Liberman *et al.* engineered hydrogels by combining sulfated polysaccharides from three different types of red microalgae: *Porphyridium* sp., *Dixoniella grisea*, and *Porphyridium aerugineum* with the addition of chitosan and zinc. These nanoparticles serve as a physical barrier against bacterial contamination while preserving a moist environment, so enhancing biocompatibility and mechanical qualities. To enhance the hydrogels' antibacterial properties, zinc was included. The antibacterial activity of chitosan alone is strong. Their increased mechanical qualities regulated and sustained release, and ability to maintain a moist environment highlight the promise of antimicrobial Zn-PS-Chi hydrogels as efficient wound dressings. Zone ratios surrounding Zn2500-PS-Chi hydrogels were most clearly seen when tested against the Gram-positive bacteria *B. subtilis* and the fungus *C. albicans*, where the greatest inhibition was observed.^210^ Researchers have shown that nanoparticles with innovative shapes exhibit distinct optical, electrical, and catalytic capabilities, setting them apart from traditional spherical forms. This diversity of morphologies opens new possibilities for their use in medicine and biology. Using eco-friendly process, Jaffar and his colleagues produce flower-shaped carrageenan AgNPs nanoparticles with a distinct shape. Because carrageenan's surface is negatively charged and contains carboxyl, hydroxyl, and ester sulfate groups that can readily interact with positively charged metal ions *via* electrostatic attraction which helps to form a protective layer on the surface of the AgNPs. c-AgNPs effectively eliminated *S. aureus* and *E. coli* germs. The inhibition zones for *E. coli* and *S. aureus* were found to be 8.0 ± 0.0 to 11.7 ± 0.6 mm and 7.3 ± 0.6 to 9.7 ± 0.6 mm, respectively. As the concentration of c-AgNPs increased from 0.1 mg mL^−1^ to 4 mg mL^−1^, these zones became larger in size [Fig. 10C].^202^ S. Hu *et al.* synthesized multivalent virus-blocking nanomaterials by incorporating amino acids into sulfated cellulose nanofibrils (SCNFs) by the Mannich mechanism. The resultant sulfated nanocellulose treated with amino acids showed a significant improvement in its antiviral activity. Nanofibrils modified with amino acids may attach to phage X174 and form linear clusters, blocking the virus's ability to infect hosts, according to an atomic force microscope (AFM) [Fig. 10D].^203^

##### 6.1.4.1 COVID-19

Coronaviruses are a group of respiratory viruses that may affect both people and other animals. The presence of spike proteins on their surface gives them a characteristic crown-like look when seen under a microscope. Coronaviruses come in various forms, from the ones that cause the common cold to those that cause more serious illnesses like MERS, SARS, and the new coronavirus SARS-CoV-2, which is responsible for COVID-19. Coronaviruses come in various forms, from the ones that cause the common cold to those that cause more serious illnesses like MERS, SARS, and the new coronavirus SARS-CoV-2, which is responsible for COVID-19. Coronaviruses predominantly transmit by respiratory droplets emitted when an infected individual coughs, sneezes, or speaks. The virus can also be transmitted by contacting infected surfaces and subsequently touching the face. COVID-19 treatments have advanced, incorporating antiviral drugs and supportive care for critical patients. Research into the structure, transmission, and possible treatment options of the virus is ongoing; one such area of study is the use of SPs as antiviral medicines against coronaviruses. Several distinct structural characteristics, including molecular weight (MW), sulfation levels (SL), and patterns, impact the antiviral effectiveness of SPs against coronaviruses. Increased sulfation levels can improve binding affinity to viral spike proteins, which is crucial for preventing virus attachment to host cells. Heparin is a powerful antiviral drug; however, it can cause bleeding. On the other hand, there are marine sulfated glycans (MSGs) that show promise as broad-spectrum antiviral medicines in clinical trials due to their antiviral effectiveness and decreased anticoagulant action. The mechanism of action involves MSGs obstructing spike glycoprotein connections with the ACE2 receptor on target cells, exhibiting efficient suppression at low microgram per mL concentrations without notable damage. Evidence for a competitive inhibition mechanism comes from time-of-addition tests, which show that MSGs can only suppress entrance if added either before or just after viral-like particle (VLP) addition.^211^ Glycosaminoglycans, glycans derived from marine sources (such as sulfated fucans, fucosylated chondroitin sulfates, fucoidans, and rhamnan sulfate), pentosan polysulfate, and mucopolysaccharide were all tested for inhibitory activity using surface plasmon resonance according to Yang J. *et al.* many viruses rely on the presence of heparan sulfate (HS), a cofactor with a strong negative charge, on the surface of host cells to aid in viral attachment and entrance into cells. Thus, preventing viral infection may be as simple as blocking the contact between viruses and HS. The findings demonstrate that charge interactions are crucial to antiviruses. No significant connections between the structural aspects of the sulfated glycans and their binding capabilities have been found, despite the fact that all of the tested glycans showed robust inhibitory action against both viral proteins in the SPR-based binding to surface-immobilized heparin.^212^ Yim *et al.* reported analogous findings, wherein sulfated fucoidan and crude polysaccharides, derived from six seaweed species (*Sargassum horneri*, *Undaria pinnatifida* Sporophyll, *Hizikia fusiforme*, *Laminaria japonica*, *Porphyra tenera*, *Codium fragile*), and *Haliotis discus hannai* (abalone viscera), were evaluated for their inhibitory efficacy against the entry of the SARS-CoV-2 virus. The majority of these compounds exhibited notable antiviral effects against SARS-CoV-2, with an IC50 ranging from 12 to 289 μg mL^−1^. Of particular note was *Sargassum horneri* (CPSH), the compound with the greatest carbohydrate content at 99.1%.^158^ No vaccination, effective treatment, or preventative measures exist at this time. Similar to other betacoronaviruses, SARS-CoV-2 employs its spike glycoprotein (SGP) to facilitate attachment and entrance. Evidence suggests that SGP binds to glycosaminoglycans such as heparan sulfate, in addition to the well-established interaction with its receptor, human angiotensin-converting enzyme 2 (hACE2). Using HEK293T cells, the pLV vector effectively pseudotyped SGP and generated substantial titers. The antiviral activity and affinity to SGP of several sulfated polysaccharides were determined by structural variations; these polysaccharides effectively neutralized pLV-S pseudotyped virus.^213^ In addition to these, Table 5 lists a variety of SPs that exhibit anti-microbial and anti-viral properties.

**Table 5:** List of sulfated polysaccharide and their anti-microbial/bacterial/viral activities

Sulfated polysaccharide	Modification	Anti-microbial/bacterial/viral activities	Properties	Ref.
**Unmodified SPs**				
Ulvan	Unmodified	Vesicular stomatitis virus	- Inhibiting the infection and replication of vesicular stomatitis virus	64
			- Functional agent	
			- Affected by molecular weight	
Polymannuroguluronate sulfate (alginate)	Unmodified	Human papillomavirus (HPVs)	- Inactivate HPV particles	91
			- Prevent virus capsid L1 protein binding	
			- Down- regulate the amounts of the E6 and E7 viral oncogenic proteins	
Sulfated fucoidan and crude polysaccharides (six seaweed and *Haliotis discus hannai*)	Unmodified	SARS-CoV-2	Strong, medium, and weak inhibition, the highest carbohydrate contents have the highest inhibiting activity	158
Lambda-carrageenan	Unmodified	COVID-19, influenza viruses	- Inhibition of viral entry	172
			- Protected 60% of mice from virus-induced mortality	
*Fucosylated* chondroitin sulfates (sea cucumber)	Unmodified	Anti-enterovirus 71	Mw, sulfated branches, pattern, percentage content of sulfate. Significantly influenced FCS's inhibitory activities against EV71	205
Iota-carrageenan	Unmodified	COVID-19, common cold	- Enhance recovery rates from the common cold by around 50% and reduce the length of prolonged colds by roughly 30%	208
			- Impact on COVID-19 at the clinical level	
Sulfated glycans	Unmodified	Anti-viral, anti-coronaviruses	Mw, sulfation levels (SL), and patterns	211
Sulfated glycans	Unmodified	Anti-MERS-CoV	Charge interactions	212
Heparin	Unmodified	COVID-19	- Inhibition of viral entry	213
			- Affinity to SGP	
*Alaria marginata*, *Alaria ochotensis*, *Laminaria longipes*, *Saccharina cichorioides*, *Saccharina gurianovae*, and *Tauya basicrassa*	Unmodified	Anti-HIV-1	Affecting the early stages of the virus–cell interaction, blocking the virus' attachment to and entry into the host's cell, with a selectivity index (SI) >160	214
Fucoidans (brown algae)				
Fucoidan	Unmodified	Antibacterial (*Lactobacillus rhamnosus*)	- Probiotic modulation	215
			- Inhibiting bacterial infections	
Iota-carrageenan	Unmodified	SARS-CoV-2	Even in concentrations as low as 6 μg per mL inhibits *in vitro*	216
Iota-carrageenan	Unmodified	SARS-CoV-2	- Inhibits viral replication	217
			- Neutralized with an IC50 value of 2.6 μg mL^−1^	

**Modified SPs**				
Carrageenan (λ-κ-, X-ι/κ CRG)	Copolymer	Anti-HIV-1	The degree and position of sulfation and the polymer conformation	204
Fucoidan (*Fucus evanescens*)	Modified with enzyme	HSV-1, HSV-2, ECHO-1, HIV-1	Inhibition of the early stage of virus replication, improved the survival rate, alleviated symptoms of the disease, prevented the weight loss, and reduced vaginal virus load induced by HSV-2 infection, efficacy against many DNA and RNA viruses	205
Sulfated derivatives of glucose, maltose, β-cyclodextrin	Sulfated	Anti-HCMV	High degrees of sulfation, structural, Mw	218
		Anti-HSV-1		
*Lycium barbarum*	Sulfated	Anti-HIV-1	Molecular weight, DS values	193

**Hybrid SPs**				
κ-Carrageenan	Konjac glucomannan/TiO_2_ nanocomposite	Anti-microbial anti-fungi	Exhibited effective photocatalytic anti-fungal activity (79%) for *Penicillium viridicatum*	209
Heparin	Ag NPs	Anti-microbial	Irrespective of their shape-size	141
Sulfated polysaccharides (sea, brackish or fresh water)	Zinc-chitosan-sulfated polysaccharides hydrogels	Anti-microbial	A wide range of antibacterial activity	210
		Anti-fungi		
Carrageenan	Ag NPs	Anti-bacterial	Promising antibacterial activity against *E. coli and S. aureus*	202
Sulfated cellulose	- Sulfated	Anti-viral	- Multivalent viral inhibitors	203
	- Nanofibrils		- Preventing the virus from infecting the host	
	- Amino acid			
Agar	Zn-carbonate and Zn-phosphate/agar nanocomposite	Anti-microbial	Strong antimicrobial activity against *Staphylococcus aureus*	219
Kappa-carrageenan	Ag NPs	Anti-bacterial	Good antibacterial activities against *Staphylococcus aureus*, methicilin resistant *Staphylococcus aurous*, *Peseudomonas aeruginosa* and *Escherichia coli* with maximum zones of inhibition 11 ± 2 mm	220
*Arthrospira*	Chitosan nanocomposite	Anti-bacterial, anti-viral	Gram-positive bacteria (killed ∼45%) and Gram-negative bacteria (killed ∼30%) as a drug carrier	221

#### 6.1.5 Anti-tumor/cancer activity

**Fig. 11:**
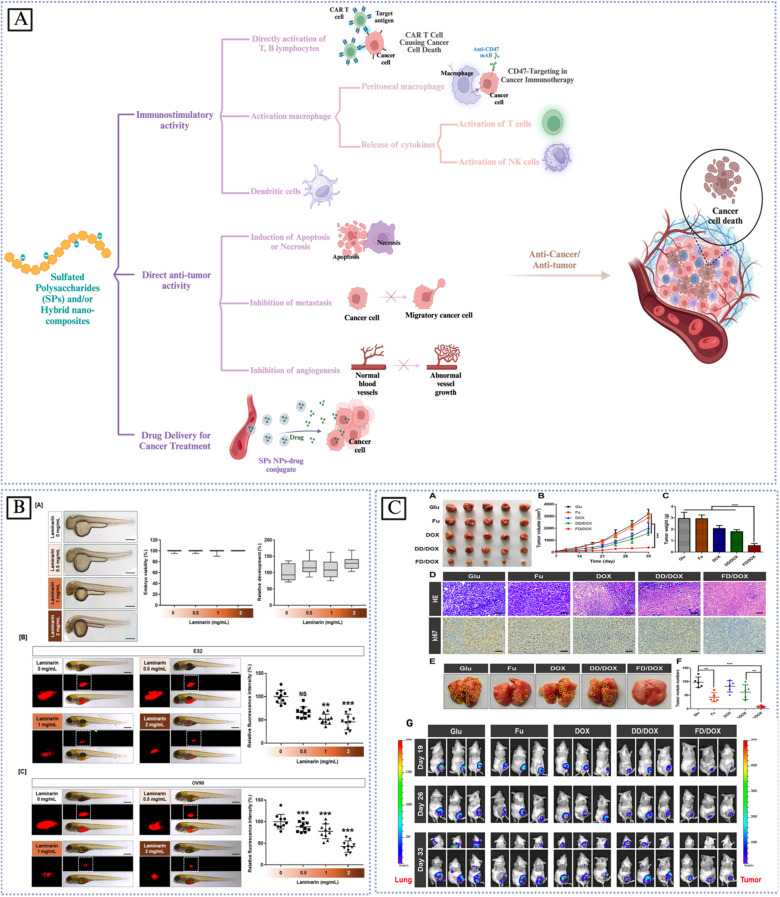
(A) Schematic diagram mechanisms of anti-cancer/anti-tumor of sulfated polysaccharides and Hybrid bio-nanocomposites. Created in BioRender. Alfinaikh R. (2025) https://BioRender.com/e71j328. (B) Effects of laminarin on cytotoxicity and tumor formation *in vivo*. (A) Zebrafish embryos, with their eggshells removed, were treated with various concentrations of laminarin (0.5, 1, and 2 mg mL^−1^) for 24 h. Under light microscopy, normal zebrafish viability and development were observed following laminarin treatment. (B and C) Laminarin-treated OC cells were injected into zebrafish yolks to form a xenograft model. Adapted from ref. 222. With permission from [MDPI], copyright [2020]. (C) Anti-tumor and anti-metastasis effect on 4T1 spontaneous metastasis model. (A) Image of 4T1 tumors treated with Glu, Fu, DOX, DD/DOX or FD/DOX (DOX-equivalent 2.5 mg kg^−1^), respectively (n Z 5). (B) The changes of tumor volume during treatment. Data are presented as mean SD (n Z 5), ***P < 0.001. (C) The weight of tumors after treatment. Data are presented as mean SD (n Z 5), ***P < 0.001. (D) H&E staining and ki67 staining for tumors. Scale bar *Z* 100 mm. (E) Images of lungs in different groups. The yellow circles represented metastatic nodules on lungs. (F) Count of metastatic nodules on lungs. Data are presented as mean SD (n Z 5), **P < 0.01, ***P < 0.001. (G) Anti-tumor and anti-metastasis effect on 4T1 spontaneous metastasis model; bioluminescence imaging of tumors and lung metastasis in tumor-bearing mice treated with Glu, Fu, DOX, DD/DOX or FD/DOX (DOX-equivalent 2.5 mg kg^−1^), respectively (n Z 3). Adapted from ref. 223. With permission from [Elsevier], copyright [2021].

Cancer is a serious global public health concern. The diversity, complexity, and partial understanding of cancers characterized by uncontrolled cell proliferation complicate their treatment.^197^ When it comes to global public health, cancer ranks high among the most pressing issues. It is challenging to find a cure for cancer since the diseases caused by uncontrolled cell proliferation are varied, complicated, and little understood. There has been a doubling of the cancer incidence rate in the last decade, and 8.2 million people die from cancer every year, making it the leading cause of death globally (and accounting for 13% of all deaths). Rising temperatures, unhealthy diets, free radicals, and altered ways of living all contribute to the spread of this terrible human disease.^119^ Radiation therapy, surgery, and systemic treatments such as chemotherapy, targeted therapy, hormone therapy, and immunotherapy are the current mainstays in cancer care. Currently, chemical therapy is the mainstay for treating tumor diseases. Most chemotherapeutic medications can enhance immunity or cause tumor cell death, according to studies. However, these drugs also have detrimental effects on normal cells in the body, which can be dangerous or even fatal. Research on marine biomaterials has been ongoing for some time, and the results show that many of these materials have strong anticancer properties with negligible hazardous side effects. Many bioactive metabolites with therapeutic efficacy and very unusual structure can be created from marine-derived organisms because of their particular environment, culture conditions, and separation methods.^69^ Consequently, numerous radical-scavenging natural substances, including sulfated polysaccharides, have been suggested for their advantageous properties as cancer prevention agents.^119^ With little or no side effects, the majority of natural anticancer substances can control the proliferation of cancer cells. With little or no side effects, the majority of natural anticancer substances can control the proliferation of cancer cells. An increasingly important global strategy in cancer prevention is the identification of innovative, effective natural therapeutic agents for cancer. Multiple investigations have shown that sulfated polysaccharides possess cytotoxic properties against cancer cell lines and inhibit tumor growth in mice.^112^ The anticancer mechanism of polysaccharide is based on a preventative method where active preparations are consumed directly. It inhibits tumor cell proliferation and induces tumor cell apoptosis. Concurrently, increasing the immune system's ability to destroy tumor cells [Fig. 11A].^31,44^ Sulfated polysaccharides outperform non-sulfated polysaccharides in terms of anti-cancer effectiveness, according to research by M.-K. Lu *et al.* They performed a battery of *in vitro* experiments to determine whether the sulfated polysaccharide (SPs) found in the edible fungus *Antrodia cinnamomea* has any anti-cancer properties, because it has recently been shown to be a new immunomodulatory drug. Of its kind, their research pinpoints SPs as the critical signaling molecule responsible for inhibiting TGFR degradation, Caspase 3 activation, and PARP-mediated cell migration and viability in lung cancer. The findings indicated that sulfated polysaccharides are essential for the stimulation of TGFR breakdown. Hence, SPs may exhibit considerable promise as a dietary supplement or treatment approach to inhibit lung cancer cell proliferation.^224^ Comparable studies have indicated that sulfated glucose-rich polysaccharides including glucosamine, galactose, and mannose (ZnF3) not only directly inhibit cancer cells but also stimulate macrophage-mediated cytotoxic effects on cancer cells.^225^ It has also been reported that SPs assess the data suggesting that sulfated galactoglucan derivatives (Sul-CDA-0.05) can disrupt Smad/Id1 signaling, which in turn blocks angiogenesis and the proliferation of lung cancer cells in both *in vitro* and *in vivo* studies by targeting BMPRIA and BMPRII and preventing the production of BMP2 and VEFG.^226^ Additionally, SPs, namely *Gracilariopsis lemaneiformis*, may enhance chemotherapy's sensitivity to cancer by acting in conjunction with it. By combining SPs with Cisplatin (CP), one of the most powerful cytotoxic drugs used to treat colorectal cancer, tumor growth was considerably reduced. Increased ferroptosis, which may have been achieved by targeting Tfrc and the SLC7A11/Gpx4 pathway, and hence amplified CP's anticancer effects. On top of that, cancer immunotherapy was enhanced when SPs and CP were administered together. This may have something to do with the NOD-like receptor, toll-like receptor, T cell receptor, PD-L1 expression, and PD-1 process.^227^ Laminarin has demonstrated efficacy as a powerful agent for cancer prevention and suppressed the growth of cancer cells. The antitumor experiment demonstrated that laminarin's anti-tumor activity was greatly amplified following sulfated modification. Moreover, at the same dose, sulfated laminarin exhibited a substantially higher inhibition rate than laminarin. Substituting the hydroxyl group of a laminarin sugar unit with a sulfate group alters the structure of the sugar chains, facilitating the development of a non-covalent bond. Furthermore, repulsions among the anionic groups extend the sugar chain, and some sulfuric acid and hydroxyl groups on the sugar chain may establish hydrogen bonds. This may lead to the chain adopting a helical shape and an active conformation, hence enhancing the polysaccharide's biological activity.^228^ Bae *et al.* prove that laminarin has anticancer properties in human ovarian cancer (OC) cells. Laminarin prompted cell death by DNA breakage, formation of reactive oxygen species, activation of apoptotic signals, induction of endoplasmic reticulum (ER) stress, modulation of calcium levels, and modification of the ER-mitochondria axis. In a zebrafish xenograft model, it inhibited tumor development without causing cytotoxicity [Fig. 11B].^222^ Tumor cells may recruit and activate platelets, which adhere to circulating tumor cells (CTCs) and facilitate the metastasis of tumor cells to distant organs. Thus, nanoparticles hitching on activated platelets may target primary tumors, CTCs, and distant organ metastases. Consequently, tumor-homing platelets that have been activated release TGF-b, a growth factor that promotes tumor progression and suppresses the immune system. An effective multi-talent approach is necessary for reducing immunosuppressive signals and ensuring accurate tumor surveillance. A micelle laden with fucoidan and DOX was produced by Guo R. *et al.* This micelle attached to activated platelets *via* P-selectin. Metastatic cancer treatment was made possible by fucoidan-coated nanomaterials that triggered platelet-targeting micelles, which successfully tracked tumor cells and changed the microenvironment in primary tumor and premetastatic organs [Fig. 11C].^223^ In a similar study, using tumor-targeted, self-assembled nanoparticles, Yang *et al.* regulated mitochondrial metabolism and BACH, a transcription factor with elevated expression in tumors of triple-negative breast cancer patients. To target tumors, BH nanoparticles (BH NPs) were surface-modified with chondroitin sulfate (CS) *via* robust electrostatic interactions after their synthesis utilizing the BACH1 inhibitor hemin and the mitochondrial function inhibitor berberine derivative (BD). The findings *in vitro* and *in vivo* demonstrated that CS/BH NPs may be efficiently transported to tumor cells and specifically target mitochondria. The tumor suppression rate of CS/BH NPs in a tumor xenograft nude mice model reached 74.5% without detectable toxicities to other organs, illustrating the superior effectiveness and safety of CS/BH NPs.^229^Table 6 lists a variety of SPs that have anti-cancer and tumorigenic properties.

**Table 6:** List of sulfated polysaccharide and their anti-tumor/cancer

Sulfated polysaccharide	Modification	Disease	Model source & cell line	Anti-tumor/cancer properties	Ref.
**Unmodified SPs**					
κ- and λ-carrageenan (*Chondrus armatus*)	Unmodified	Colon cancer	KYSE-30, FLO-1, HCT-116 and RKO cell lines	κ-Carrageenan inhibited the G1 phase (in RKO, the S phase in FLO-1 and HCT-116 cell lines), and the G2 phase (in KYSE-30)	52
				λ-Carrageenan inhibited the S phase of FLO-1 and G1 in KYSE-30 esophageal cell lines	
*Codium bernabei* (green algae)	Unmodified	Anti-tumor	Colorectal carcinoma (HTC-116) cell line, breast cancer (MCF-7) and human leukemia (HL-60) cell	The cytotoxic effect of TPs and APs	122
*Calocybe indica* (mushroom)	Unmodified	Anti-tumor	MTT assay, human cells	Inhibited the growth of HeLa, PC3, HT29, HepG2, and Jurkat cells	133
*Undaria pinnatifida* (brown seaweed)	Unmodified	Breast cancer	DMBA-induced breast cancer rats' model	Polarity and bilayer structure after high-dose SPUP therapy	150
*Antrodia cinnamomea* (mushroom)	Unmodified	Lung cancer	Human lung cancer A549 cells, mouse lung cancer LLC1 cells	Inhibits A549 and LLC1, activation of caspase 3 and PARP	224
Sulfated galactoglucan	Unmodified	Lung cancer	Human microvascular endothelial cells (HMEC-1), A549 lung cancer cells, BxPC-3 pancreatic cancer cells and LO_2_ liver cells	Targeting both BMPRIA and BMPRII	226
*Gracilariopsis lemaneiformis* (red alge)	Unmodified	Colon-26 carcinoma	BALB/c mice, C26 colorectal models	Targeting the transferrin receptor and SLC7A11/Gpx4 pathway to induce ferroptosis	227
Laminarin	Unmodified	Ovarian cancer	Human OC cells	Decreased PI3K and MAPK signaling, inducing cell death *via* DNA fragmentation, generating reactive oxygen species, triggering apoptotic signals, causing endoplasmic reticulum (ER) stress, regulating calcium levels, and modifying the ER mitochondria axis	222
*Poseidonocella Sedimentorum* KMM 9023T (marine microorganisms)	Unmodified	Anticancer activity *in vitro* (clonogenic assay)	Human HT-29, MCF-7 and SK-MEL-5 cells	Inhibition of studied cells	230
Dextran sulfate	Unmodified	Gastric cancer	- GES-1, MKN-45, HGC-27, BGC-823, AGS and SGC-7901 cells	Inhibit the proliferation and metastasis of gastric cancer cells by regulating miR-34c-5p	231
			- BALB/c mice		
λ-Carrageenan	Unmodified	Anti-tumor	B16-F10 and 4T1 bearing mice	- Increasing tumor-infiltrating M1 macrophages, DCs and activated CD4^+^ CD8^+^ T lymphocytes in spleen	232
				- Enhance the secretion of IL17A in spleen	
				- Increase the level of TNF-α in tumor	
				- Enhanced the production of anti-OVA antibody	
				- Enhance tumor immune response	
Five red seaweeds a green and two brown seaweeds	Unmodified	Anti-cancer	HeLa and MCF cell lines	- Green *C. fragile* shows L-form with negative rotation	233
				- The cytotoxicity was ascertained depending on the sulfate content	
λ-Carrageenan	Unmodified	Breast cancer	Human breast cancer cells (MDA-MB-231)	- Inhibited the growth of MDA-MB-231 cells by upregulating the pro-apoptotic genes caspase-8, caspase-9, and caspase-3, resulting in elevated amounts of active caspase-3 protein	234
		Anti-Proliferative		- Hinder mitochondrial function by modifying the bax/bcl-2 expression ratio	
λ-Carrageenan	Unmodified	Anti-tumor	TC-1 tumor mouse model	- Inhibit tumor growth	235
				- Increase the frequencies of CD4^+^ and CD8^+^ T cells in spleens of tumor mice	
				- Promoted dendritic cells maturation through TLR4 signaling pathway	
				- Strong CD8^+^ T cell responses	
Ulvophyte (green algae)	Unmodified	Anti-cancer	*In vitro*, *in vivo* studies, human clinical trials	Exhibited extensive anticancer activity against leukemia, colorectal, hepatoma, and breast cancer cell lines	236

**Modified SPs**					
Laminarin	Sulfated	Anti-tumor	MTT assay	- Potent agent for cancer prevention and inhibited	228
				LoVo human colon cancer cell proliferation	
				- Antitumor activity of laminarin was enhanced after sulfation	
*Cordyceps gunnii* mycelia	Sulfated	Anti-tumor	K562 cells	The tumor inhibition ratio of SPS50 against K562 cells was 69.92%	237
*Undaria pinnatifida*	Sulfated	Anti-tumor	Tumor-bearing mice	Exhibited anti-tumor activity *in vivo*	238
*Siraitia grosvenorii*	Sulfated	Anti-tumor	HepG2, MDA-MB-231, A549	- Inhibit the growth of human hepatoma cells (HepG2), human breast cancer cells (MDA-MB-231) and human non-small cell lung cancer cells (A549) *in vitro*	239
				- Decrease the mitochondrial membrane potential, induce apoptosis, and alter the expression of apoptosis-related mRNA and protein	

**Hybrid SPs**					
Fucoidan	DOX-loaded micelle nanomaterials	Anti-tumor	- Mouse breast cancer cells (4T1)	- Accurately monitoring and destroying tumor cells	223
			- 4T1-Luc cells	- Inhibited TGF-b expression	
			- Female BALB/c mice	- Targeting micelle effectively tracked tumor cells	
				- Remodeled the microenvironment in primary tumor and premetastatic organs	
Chondroitin sulfate (CS)	Self-assembled nanoparticles (BH NPs)	Triple negative breast cancer (TNBC)	- MDA-MB-231 murine breast cancer models	- Decreasing the amounts of tumor cell metabolites, glycolysis and metastasis-associated proteins	229
			- BALB/c nude mice	- Effectively delivered to the tumor cells and target mitochondria	
				- The tumor inhibition rate in a tumor xenograft nude mouse model was up to 74.5%	
κ-Carrageenan	Graphene oxide nanocarrier	Cervical cancer	HeLa cell line	- Targeted and synergetic therapy for cervical cancer therapy showing significant cell death of cancer cells	240
	Conjugated with biotin			- High nuclear condensation of HeLa cell line	
Sulfated xyloglucan	Magnetic nanocomposite, quercetin sulfate	Antitumor activity	Leukemia HL-60, glioblastoma SNB-19, colorectal carcinoma HCT-116, prostate PC3	HL-60 (IC50 = 1.58 ± 0.54 μg mL^−1^)	241
				SNB-19 (IC50 = 8.77 ± 0.36 μg mL^−1^)	
				HCT-116 (IC50 = 8.69 ± 0.21 μg mL^−1^)	
				PC3 (IC50 = 17.42 ± 3.57 μg mL^−1^)	

## Conclusions

In light of growing environmental consciousness and the alarming increase in the number of diagnosed diseases such as cancer, heart disease, and COVID-19, today's scientific knowledge and technological innovations are increasingly geared toward long-term sustainability. Sulfated polysaccharides, biocompatible biopolymers with numerous practical applications, are distinct from many synthetic chemical inhibitors commonly found in medicinal products. SPs possess numerous advantages, such as their biocompatibility, abundance, sustainability, and, most importantly, their biological properties. The biological activities of SPs include anticoagulant, immunomodulator, anticancer, antibacterial, anti-inflammatory, and antioxidant properties. Sulfated polysaccharide structure research and specialized biological tests are useful tools for studying how biological activities might work at the molecular level, in addition to their clear applications. So, making sulfated polysaccharides nano- and/or bio-composites is closely linked to making new medicines. These bio- and nanocomposites then help the economy grow by providing a wider range of goods and, finally, high-value goods. Several surface modifications and chemical treatment methods may overcome limitations such as hydrophilic nature and biocompatibility. This allows for the possibility of modifying biomaterial characteristics to improve their mechanical, thermal, and physiological overall performance in a wide range of contexts. This groundbreaking investigation identifies critical potential application areas for SPs that may result in innovative advancements in scientific research and therapeutic applications. Therefore, from the perspective of biomedical applications, there are significant opportunities to: (1) develop and translate naturally or chemically sulfated polysaccharides that exhibit antioxidative, anticancer, antiviral, and anti-inflammatory bioactivities; (2) create bio-composites by integrating copolymers with sulfated polysaccharides, such as PVA and chitosan; and (3). Bio-nanocomposites are created by integrating nanoparticles into naturally occurring or modified sulfated polysaccharides, including inorganic nanomaterials (ZnO nanoparticles) or organic nanomaterials (cellulose nanoparticles). Bio-nanocomposites made from SPs have the potential to produce new types of materials. Consequently, further developments in bio-nanocomposites utilizing hybrid sulfated polysaccharides are expected.

This review focuses on the progress made in the development of innovative SPs bio-nanocomposites, which hold numerous potential applications in fields such as biomedicine, biotechnology, pharmaceuticals, tissue engineering, and nanotechnology. Sulfated polysaccharides and their nano/bio-composites have a lot of potential as antioxidation, anticancer, antiviral, and anti-inflammatory drugs in medicine, and we are just getting started. Nonetheless, several critical issues persist:

• Interactions among nanoparticles across various sizes and matrix chains constitute a significant problem, necessitating deeper knowledge to customize the characteristics of composites.

• Surface modification, which increases the electrostatic forces between the nanoparticles and inhibits agglomeration, can achieve uniform dispersion of nanomaterials, effective interfacial adhesion, and homogeneity in the composite during preparation.

• The substantial expense associated with composite fabrication and nanomaterials manufacturing is a significant obstacle, prompting researchers to concentrate on creating economical substrates for the large-scale manufacture of nanomaterials at reduced costs. Improvements in manufacturing and processing techniques, along with synthetic procedures like chemical, microwave-assisted, or sonication methods for combining inorganic and organic nanocomposites, including environmentally sustainable nanoparticle synthesis using various plant extracts, could significantly reduce these costs. Recycling of the nanoparticles provides an alternative solution.

• Further development of distinctive nanocomposites with recyclable and biodegradable characteristics is necessary.

• Enhancing the attributes of SPs to match those of synthetic polymers, particularly regarding quality and performance, presents a primary challenge, necessitating a greater focus on the complete elimination of synthetic polymers.

• The biosafety of SP bio-nanocomposites remains a significant public issue owing to insufficient clinical evidence about their safety and efficacy in humans. Biosafety tests for these SP bio-nanocomposites are still mostly based on preliminary studies on animals and tests done *in vitro*. Enhancing our understanding of the interactions between SPs, bio-nanocomposites, and tissue cells, physiological fluids, and organs is essential. Overcoming regulatory barriers is crucial for the widespread clinical application of these materials.

• An advanced understanding of biological characteristics is essential for designing systems with customized attributes to enhance the significant potential of personalized medicine in the pharmaceutical business.

• Nanomedicines must undergo pharmaceutical stability testing and other quality investigations before they can be considered effective pharmaceutical medication products. A greater amount of work must go into studying nanomedicines in order to establish their effectiveness and safety.

• Enhancing the effectiveness of SPs bio-nanocomposites as antiviral medications is necessary to combat a wide range of viral diseases, including emerging epidemics like COVID-19. Understanding how these sulfated polysaccharides inhibit viral entrance, replication, and assembly is crucial for the development of new antiviral drugs.

With any luck, this review will lay out a general outline for future work on functional SP bio-nanocomposites and encourage both academics, scientific researchers, biotechnologists, biomedical engineers, and companies to invest in multi-faceted studies, which should speed up the clinical transformation process.

## Abbreviation

ι-CRG: Iota-carrageenan
κ-CRG: Kappa-carrageenan
λ-CRG: Lambda-carrageenan
ABTS: (2,2-Azino-bis(3-ethylbenzothiazoline-6-sulfonic acid))
APTT: Partial thromboplastin time
CL: Cellulose
ClSO_3_H: Chlorosulfonic acid
ClSO_3_-Py: Chloro-sulfate pyridine
CRG: Carrageenan
ChS: Chitosan sulfate
CS: Chondroitin sulfate
DPPH: (2,2-Diphenyl-1-picrylhydrazyl) scavenging assays
DS: Degree of sulfate groups connecting to hydroxyl groups on SPs
EAE: Enzymatic-assisted extraction
EtOH: Ethanol
FCA: Ferrous ion chelating ability
FRAP: Ferric ion reducing antioxidant power
Fuc: Fucose
GAG: Nonsulfated glycosaminoglycan
GAGs: Sulfated glycosaminoglycans
GPC: Gel permeation chromatography
HA: Hyaluronic acid
HS: Heparan sulfate
H_2_SO_4_: Sulfuric acid
H_3_NSO_3_: Sulfamic acid
HWE: Hot water extraction
IEC: Ion-exchange chromatography
MAE: Microwave-assisted extraction
Mw: Molecular weight
NCs: Nanocomposite
NK cells: Natural killer cells
NPs: Nanoparticles
ORAC: Oxygen radical absorbance capacity
PGS: Polyguluronate sulfate
PLE: Pressurized liquid extraction
PMGS: Polymannuroguluronate sulfate
PMS: Polymannuronic acid
PT: Prothrombin time
ROS: Reactive oxygen species scavenging assays
SALG: Alginate sulfate
SAR: Structure–activity relationship
sCel: Cellulose sulfate
SCMC: Carboxymethyl cellulose sulfate
SEC: Size-exclusion chromatography
SO_3_-Py: Sulfur trioxide pyridine
SPs: Sulfated polysaccharides
TT: Thrombin time
UAE: Ultrasonic-assisted extraction

## Data availability

No primary research results, software or code have been included and no new data were generated or analyzed as part of this review. Additionally, the authors obtained copyright agreement documents before including all previously published data in this review.

## Conflicts of interest

There are no conflicts to declare.
